# Evaluation of Benzo[*cd*]indol‐2(1*H*)‐ones as Downstream Hedgehog Pathway Inhibitors

**DOI:** 10.1002/open.202500119

**Published:** 2025-04-14

**Authors:** Ioannis A. Tsakoumagkos, Quentin T. L. Pasquer, Christian‐Louis Guillod, Charlotte Rossion, Meropi Bagka, Sonya Torche, Tomoyo Sakata‐Kato, James K. Chen, Sascha Hoogendoorn

**Affiliations:** ^1^ Department of Organic Chemistry University of Geneva 30 quai Ernest-Ansermet Geneva Switzerland; ^2^ Department of Chemical and Systems Biology Stanford University 269 Campus Dr., CCSR 3155 Stanford CA 94305 USA; ^3^ Department of Developmental Biology Stanford University 269 Campus Dr., CCSR 3155 Stanford CA 94305 USA; ^4^ Department of Chemistry Stanford University 269 Campus Dr., CCSR 3155 Stanford CA 94305 USA; ^5^ Present address: Department of Protozoology Institute of Tropical Medicine Nagasaki University 1-12-4 Sakamoto Nagasaki 852-8523 Japan

**Keywords:** Hedgehog pathway inhibitor, BET bromodomains, photoaffinity labeling, target identification, mechanism-of-action studies

## Abstract

Epigenetic targeting of the Hedgehog (HH) signaling pathway has emerged as a possible strategy to combat HH pathway‐driven cancers. In this study, we report on benzo[*cd*]indol‐2(1*H*)‐ones as downstream Hedgehog pathway inhibitors. We find that benzo[*cd*]indol‐2(1*H*)‐one **1** has sub‐micromolar potency in a variety of Hedgehog pathway cell models, including those with constitutive activity through loss of Suppressor of Fused. Compound **1** furthermore reduces cellular and ciliary GLI levels, and, like the BET bromodomain inhibitor HPI‐1, increases the cellular levels of BRD2. To directly assess the ability of compound **1** to bind to BET bromodomains in cells without the need of synthetic modifications, we develop a competition assay against degrader HPP‐9, the action of which was dose‐dependently outcompeted by compound **1**. Indeed, compound **1** reduces the viability of GLI‐driven lung cancer cells and medulloblastoma spheroids, with a potency similar to its inhibitory effect on the HH pathway. Taken together, our studies highlight the potential of the benzo[*cd*]indol‐2(1*H*)‐one scaffold for epigenetic targeting of the HH pathway.

## Graphical abstract

Benzo[*cd*]indol‐2(1*H*)‐one **1** inhibits the Hedgehog signaling pathway downstream of Suppressor of Fused, lowering cellular and ciliary GLI2 levels, and preventing GLI1 expression. A PROTAC‐based competition assay validated the cellular engagement of BET bromodomains, adding compound **1** to the growing set of epigenetic modulators of the Hedgehog signaling pathway.

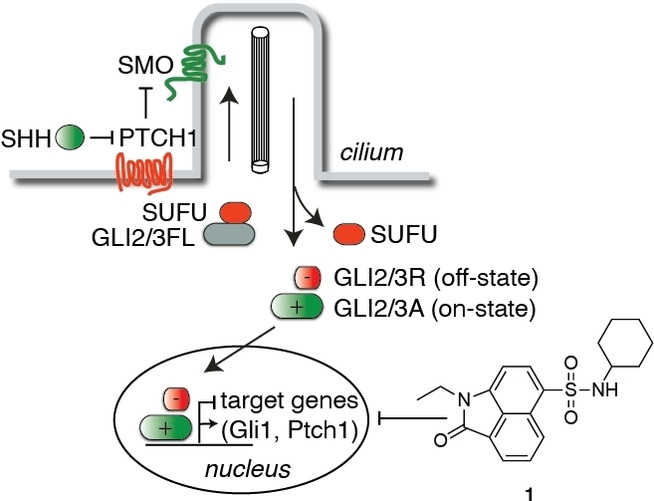

## Introduction

The mammalian Hedgehog signaling pathway plays essential roles in embryonic development, as well as tissue homeostasis and stem cell maintenance later in life.^[1]^ Signal transduction takes place in a specialized organelle, the primary cilium, which dynamically organizes key players of the Hedgehog pathway.^[2]^ In the off‐state of the pathway, the receptor for Hedgehog (HH) ligand (Sonic: SHH, Indian: IHH, or Desert: DHH), Patched1 (PTCH1), localizes to the ciliary membrane and thereby restricts the entry of the upstream activator Smoothened (SMO). Upon pathway activation through binding of HH to PTCH1,[^3^, ^4^] the inhibitory action of PTCH1 on SMO is relieved, PTCH1 translocates out of the cilium, allowing SMO to enter.^[5]^ This in turn sets in motion a cascade of trafficking events of the Glioma‐associated oncogene (GLI) transcription factors, the accumulation of which at the ciliary tip is essential for their release from the negative regulator Suppressor of Fused (SUFU), their processing into transcriptional activators, and subsequent trans‐

location to the nucleus.[^6^, ^7^, ^8^] In mammals, out of the three GLI proteins, GLI1 and GLI2 function mainly as transcriptional activators, whereas GLI3 in its repressor form ensures repression of target genes when the pathway is in the off‐state.^[9]^

**Figure 1:**
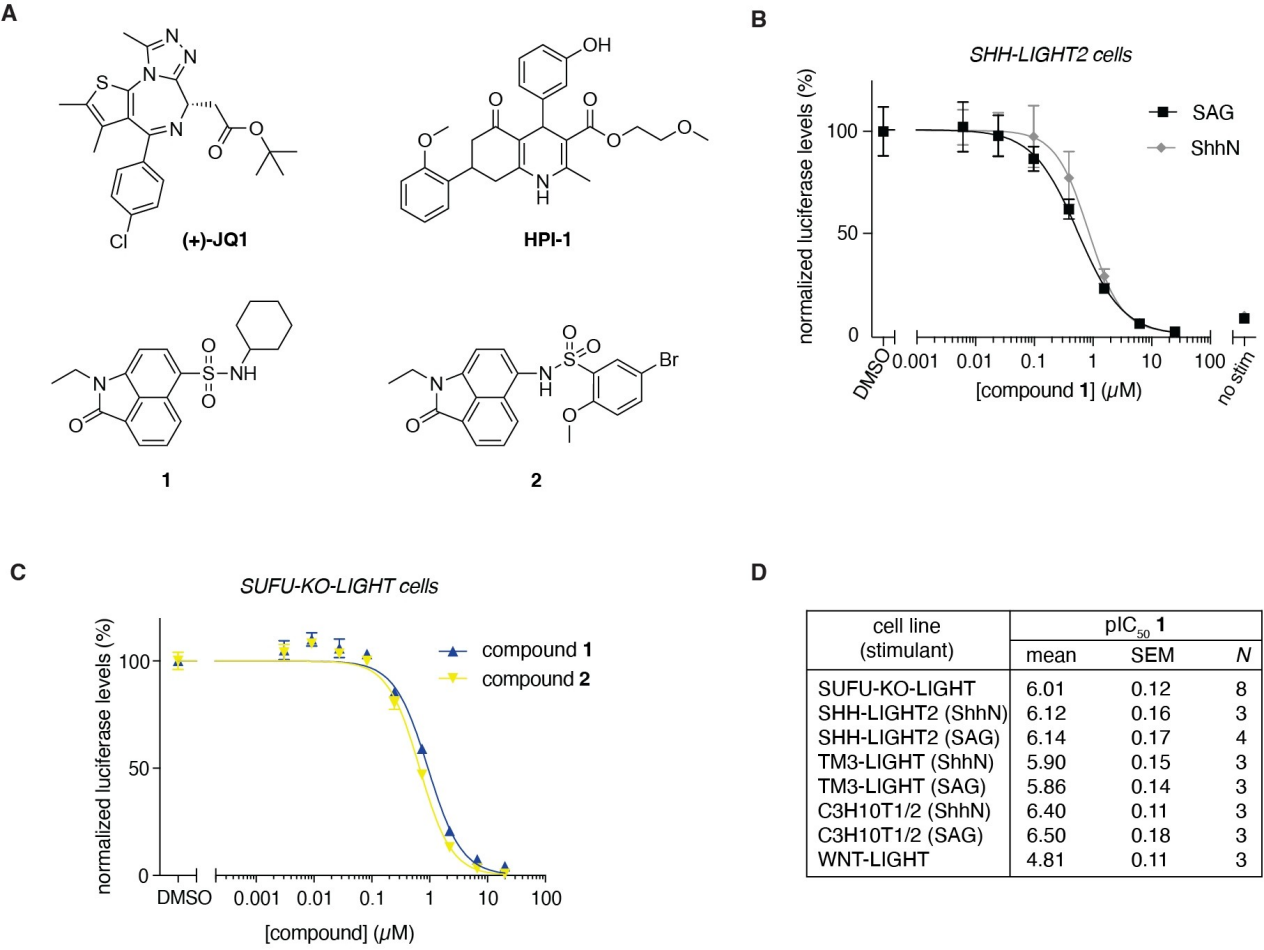
Benzo[*cd*]indol‐2(1*H*)‐ones are low micromolar HH pathway antagonists. A) Chemical structures of BET bromodomain inhibitors JQ1 and HPI‐1 and of hit compound **1** and its close analog **2**. B) Full dose‐response curves for SHH‐LIGHT2 cells treated with ShhN‐conditioned medium (ShhN) or SMO agonist (SAG) in the presence of compound **1**. C) Compounds **1** and **2** dose‐dependently inhibit constitutive HH pathway activation in SUFU‐KO‐LIGHT cells. B, C) Mean +/− SEM of three technical replicates plotted. Representative curves of three independent biological replicates. D) pIC50 values for compound **1** in a variety of different HH‐responsive cell lines and WNT‐LIGHT cells as a control. Mean ± SEM is given of N independent biological experiments.

Aberrant Hedgehog signaling underlies a variety of cancers, with notable examples being basal cell carcinoma and medulloblastoma.[^10^, ^11^] Cancers that are caused by loss‐of‐function mutations in *PTCH1* are amenable to treatment with SMO inhibitors, such as the FDA‐approved drugs vismodegib and sonidegib. Unfortunately, SMO‐inhibitor resistant tumors frequently emerge after treatment. Moreover, cancers driven by mutations in downstream pathway components do not respond to SMO inhibition, and thus there is a strong need for the identification of compounds that act downstream of SMO.[^12^, ^13^, ^14^] Epigenetic targeting of the Hedgehog pathway has emerged as an interesting strategy to combat (SMO‐inhibitor resistant) tumors. It has been shown that inhibition of the bromo and extra C‐terminal (BET) bromodomain proteins, BRD2‐4 and BRDT, by the small‐molecule inhibitors JQ1 (Figure 1A) and iBET151 prevents the recruitment of BRD4 to the *Gli1* and *Gli2* promoters.[^15^, ^16^] Recently, we have found that Hedgehog Pathway Inhibitor‐1 (HPI‐1, Figure 1A) also inhibits HH pathway activity through binding of BRD2‐4, which prevents the HH‐dependent recruitment of BRD2 and BRD4 to the *Gli1/2* loci and inhibits target gene expression. Importantly, we discovered that JQ1 and HPI‐1, while sharing the same cellular targets, are distinctly different in their behavior towards BRD2, with HPI‐1, but not JQ1, enhancing its expression levels in NIH‐3T3 mouse fibroblast cells.^[17]^ In search of novel chemical entities that can function as downstream HH pathway inhibitors, we conducted a phenotypic screen in *Sufu*‐null murine embryonic fibroblasts that were stably transfected with a GLI‐dependent luciferase reporter (SUFU‐KO‐LIGHT cells).^[18]^ From this screen, the benzo[*cd*]indol‐2(1*H*)‐one **1** (Figure 1A) emerged, a compound that has been previously identified in a structure‐based virtual screening for BET bromodomain inhibitors.^[19]^ It was characterized as a potent and selective binder of BRD2‐4 *in vitro*, with good cellular uptake and moderate anti‐proliferative effects against a variety of cell lines (MV4‐11, HL‐60, HT‐29). X‐ray crystallography confirmed that this compound binds to the same pocket as acetylated lysine, the natural binding partner of BET proteins, with the ethyl substituent mimicking the acetyl group.^[19]^ Here, we report the evaluation of compound **1** as a Hedgehog pathway inhibitor. We discover that it is a sub‐micromolar downstream HH pathway inhibitor, that, like HPI‐1, enhances the levels of cellular BRD2 in mouse fibroblasts.^[17]^ Through optimization of synthetic procedures of all‐in‐one linkers for photoaffinity labeling (PAL) experiments, we synthesized a set of PAL probes based on compound **1**. As PAL experiments proved inefficient, likely due to the loss in potency of the probes, we instead developed a label‐free competition assay to directly detect BET bromodomain engagement of benzo[*cd*]indol‐2(1*H*)‐ones in cells. We demonstrate the ability of compound **1** to outcompete the PROTAC‐mediated degradation of BRD2‐4, and its effectiveness in killing GLI‐driven cancer cells.

## Results and Discussion

### Benzo[cd]indol‐2(1H)‐ones are Downstream HH Pathway Inhibitors

As previously reported in Hom *et al*.,^[18]^ we conducted a screen of 325.120 compounds from a structurally diverse library in constitutively active SUFU‐KO‐LIGHT mouse fibroblasts. In brief, compounds were first screened at a concentration of 8 μM, and hits that showed >50 % inhibition were confirmed at 5 μM. As benzo[*cd*]indol‐2(1*H*)‐one **1** (Figure 1A) was confirmed as a hit at the 5 μM concentration, the compound was tested at 4 concentrations in SUFU‐KO‐LIGHT and WNT‐LIGHT cells (cells stably transfected with a TCF/LEF‐dependent firefly luciferase reporter)^[18]^, to exclude an effect on the related WNT pathway (Supplementary Figure 1A). No effect on WNT signaling was detected until 5 μM and so the compound was subsequently subjected to additional counterscreens including a full dose‐response for cytotoxicity (Supplementary Figure 1B), and constitutive firefly luciferase inhibitory activity (Supplementary Figure 1C). No inhibition was found for compound **1** in any of the counterscreens (Supplementary Figure 1) and thus it was selected as a lead compound for detailed activity determination studies. The compound was synthesized according to the protocol of Xue *et al*.^[19]^ To determine its potency as a Hedgehog pathway inhibitor, we first measured full dose‐response curves in both SHH‐LIGHT2 cells (NIH‐3T3 cells stably expressing a HH pathway‐dependent firefly luciferase reporter, as well as constitutive *Renilla* luciferase),^[20]^ stimulated with ShhN‐conditioned medium (ShhN) or the small molecule SMO agonist (SAG),^[21]^ and SUFU‐KO‐LIGHT cells (Figure 1B–D).^[18]^ The data was normalized against cellular toxicity measured using a CellTiter 96 colorimetric assay (Promega) (Supplementary Figure 1B, 2A), as we found that compound **1** increased *Renilla* luciferase levels, making it unsuitable as an internal control (Supplementary Figure 2B,C). As shown in Figure 1B–D, the compound exhibited similar potencies when fibroblasts were stimulated with ShhN or SAG (pIC_50_: 6.1 ± 0.2), in line with its downstream activity, and retained potency when the pathway was activated through loss of SUFU (pIC_50_: 6.0 ± 0.1). From extensive SAR studies performed by Xue *et al*.^[19]^ the inversed sulfonamide benzo[*cd*]indol‐2(1*H*)‐ one derivative **2** (Figure 1A) was found to be a slightly more potent BET bromodomain inhibitor *in vitro*, and thus we additionally synthesized and evaluated this compound in SUFU‐KO‐LIGHT cells. As shown in Figure 1C, there was no difference in the HH pathway inhibitory potency found between the two derivatives, and thus we focused the remainder of our studies on compound **1**.

**Figure 2:**
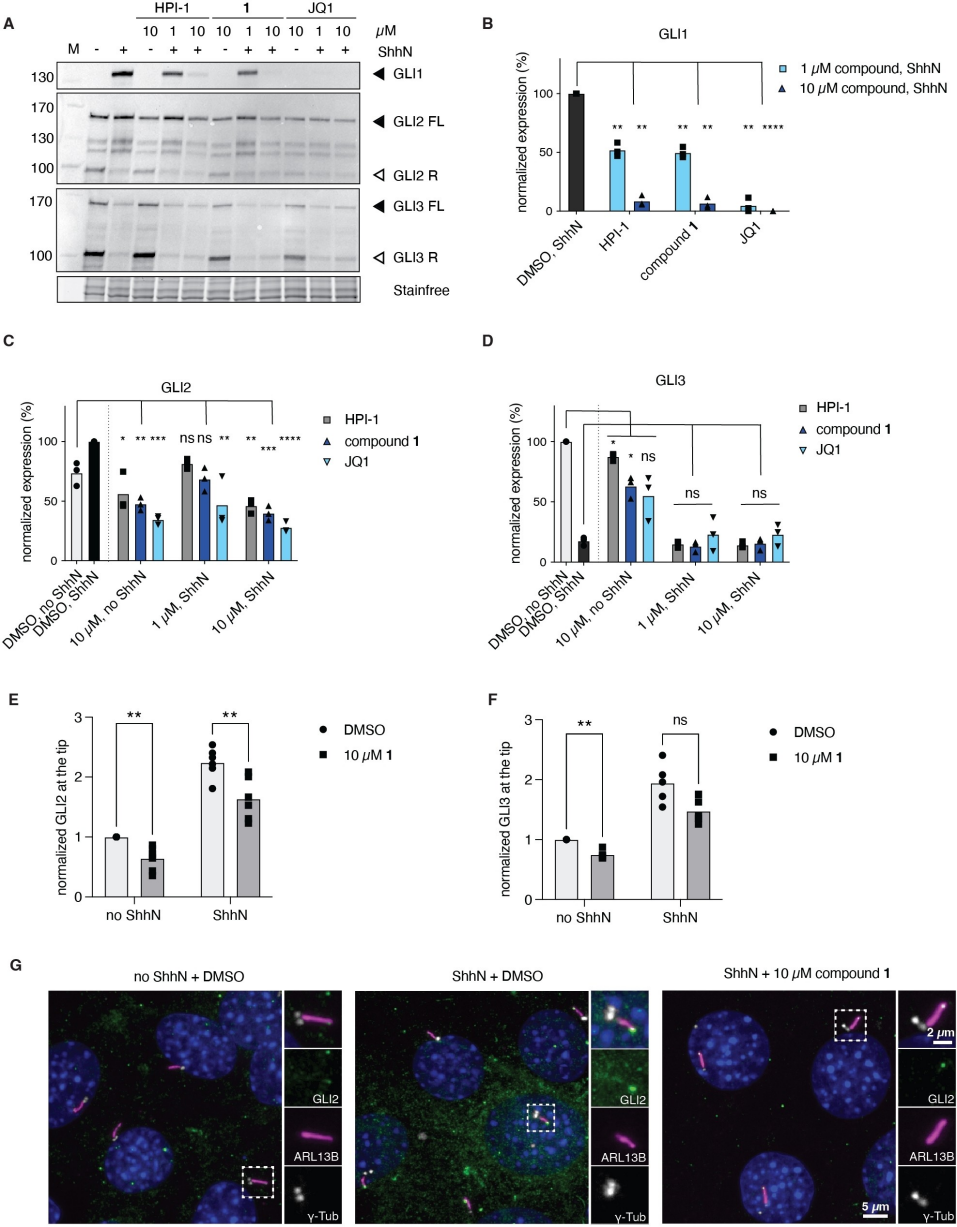
Effects of compound **1** on GLI protein expression, processing and ciliary trafficking. A) NIH‐3T3 cells were treated with varying concentrations of HPI‐1, compound **1** or JQ1 in the presence or absence of ShhN and the lysates probed for GLI proteins. Representative western blots are shown of 3 independent biological experiments, and the data is quantified for B) GLI1, C) GLI2, and D) GLI3. All bands were first corrected for protein loaded using Stainfree, before being normalized to DMSO, ShhN (B, C) or DMSO control (D) (100 %). 2‐way ANOVA with Geisser‐Greenhouse correction, Holm‐Šidák's multiple comparisons test. * p<0.0332, ** p<0.0021, *** p<0.0002, **** p<0.0001, ns: not significant. E, F) NIH‐3T3 cells were treated with DMSO or 10 μM of compound **1** +/− ShhN for 6 h and the amount of E) GLI2 or F) GLI3 at the ciliary tip was quantified. Each dot represents the mean of a biological replicate with >200 cilia quantified/experiment. Paired T‐test, ns: not significant, ** p<0.0021. G) Representative microscopy images of GLI2 trafficking.

To exclude a cell‐type specific effect, we treated the testicular epithelial TM3 cells expressing the HH pathway‐dependent luciferase reporter (TM3‐LIGHT)^[22]^ with ShhN or SAG, confirming the activity of compound **1** (Figure 1D; pIC_50_: 5.9 ± 0.1). We next evaluated the ability of the compound to inhibit the HH pathway‐dependent differentiation of C3H10T1/2 pluripotent mesenchymal cells into alkaline phosphatase‐expressing osteoblasts. Stimulation with ShhN or SAG was similarly inhibited by compound **1**, and the compound was found to be slightly more potent in this assay (Figure 1D; pIC_50_: 6.5 ± 0.1). Altogether, these results indicated that benzo[*cd*]indol‐2(1*H*)‐one **1** indeed robustly inhibited the Hedgehog pathway with submicromolar potency in a variety of cell lines. To assess a more general mechanism of inhibition, we investigated the potency of compound **1** against the related WNT signaling pathway. For this, WNT‐LIGHT cells were treated with Wnt3a‐conditioned medium in the presence of compound.^[18]^ While the compound was not completely inactive, it was 10‐fold less active against WNT signaling compared to HH signaling (Figure 1D; pIC_50_: 4.8 ± 0.1).

#### Compound 1 Prevents GLI1 Expression and Reduces GLI Accumulation at the Ciliary Tip

HH signaling strongly relies on the primary cilium, and a key step in signal transduction is the accumulation of GLI2 and GLI3 transcription factors at the ciliary tip and their subsequent processing into transcriptional activator or repressor forms.^[6]^ To investigate the effect of compound **1** on GLI protein expression and processing we turned to western blot analyses, where we included known BET bromodomain inhibitors HPI‐1 and JQ1 for comparison (Figure 2A–D). In agreement with the reporter assays, compound **1** dose‐dependently inhibited the expression of GLI1 and prevented the increase in GLI2 activator levels upon addition of ShhN, with complete inhibition at 10 μM (Figure 2A–C). Moreover, at the 10 μM concentration global GLI2 levels were reduced beyond basal levels, both in the presence and absence of ShhN (Figure 2A,C). GLI3 is proteolytically processed into an N‐terminal repressor form in the inactive state of the pathway, and total GLI3 protein levels drastically decrease upon pathway activation.[^23^, ^24^] Similarly to HPI‐1 and JQ1, compound **1** was found to have no effect on GLI3 processing upon ShhN addition, in line with a downstream target. We did, however, observe a decrease in the amount of GLI3 protein under basal conditions upon compound treatment, with a similar effect found for JQ1 and compound **1**, and a milder effect for HPI‐1 (Figure 2A,D). Next, to assess the effect of compound **1** on GLI ciliary trafficking, we treated NIH‐3T3 cells for 6 h with ShhN‐conditioned medium in the presence or absence of 10 μM compound **1** and visualized GLI2 or GLI3 by immunostaining.

As shown in Figure 2E,G, treatment of cells with 10 μM compound **1** resulted in a small reduction in GLI2 ciliary levels (−30 %, DMSO vs compound **1**) under both unstimulated and stimulated conditions. For GLI3 a similarly downward trend upon compound treatment was found (−25 %, DMSO vs compound **1**, Figure 2F), but due to the higher overall variability in the +ShhN data, this was only found to be significant under basal conditions. Generally, the fold induction upon pathway stimulation was not altered (about 2‐fold), and as such this result indicates that the available pool of GLI2 and GLI3 that can traffic to the cilium is affected by compound treatment, rather than the translocation event itself. This is in agreement with the overall lower cellular levels of GLI2 and GLI3 protein we observed by western blot upon BET bromodomain inhibition (Figure 2A, C, D).

#### Compound 1 Enhances Cellular BRD2 Protein Levels

**Figure 3:**
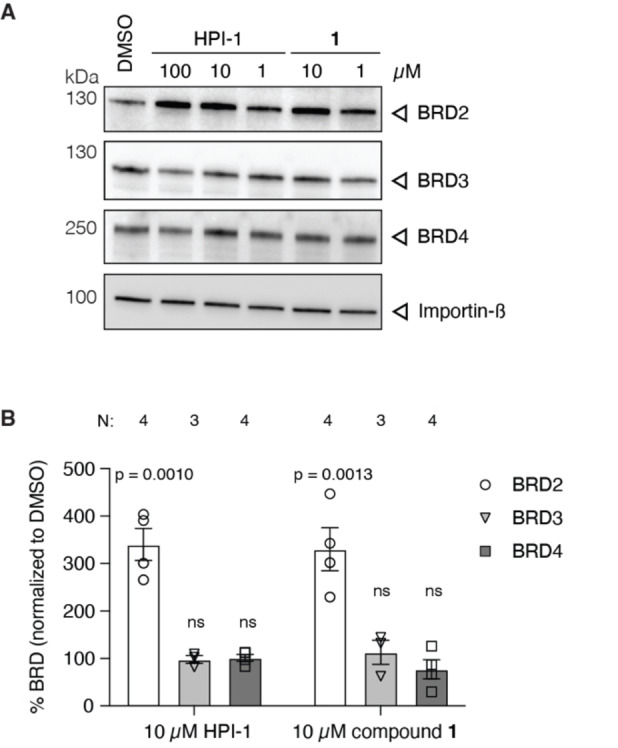
Compound **1** dose‐dependently increases BRD2 protein similar to HPI‐1. NIH‐3T3 cells were treated with HPI‐1 or compound **1** for 28 h and probed for BRD2/3/4 by western blot. A) Representative western blot and B) quantification. All bands were first corrected for protein loaded using Importin‐β, before being normalized to DMSO control (100 %). Each datapoint represents a replicate of N biological replicates total, indicated at the top. Bar represents the mean, error bars the SEM, one‐way ANOVA comparison to DMSO control (defined as 100 %, bar not shown), ns: not significant.

Previously, we found that HPI‐1 stabilized BRD2 in NIH‐3T3 cells, whereas JQ1 did not.^[17]^ We asked whether compound **1** would similarly affect BRD levels and thus NIH‐3T3 cells were treated with varying concentrations of compound **1** and lysates immunoblotted for BRD2‐4. As shown in Figure 3, we detected a striking 3‐ to 4‐fold increase in BRD2 protein when NIH‐3T3 cells were treated with 10 μM of compound, consistent with what we previously found for HPI‐1.^[17]^ No effect on the protein levels of BRD3 and 4 was detected (Figure 3B).

#### Design of Compound 1‐Based PAL Probes

**Figure 4:**
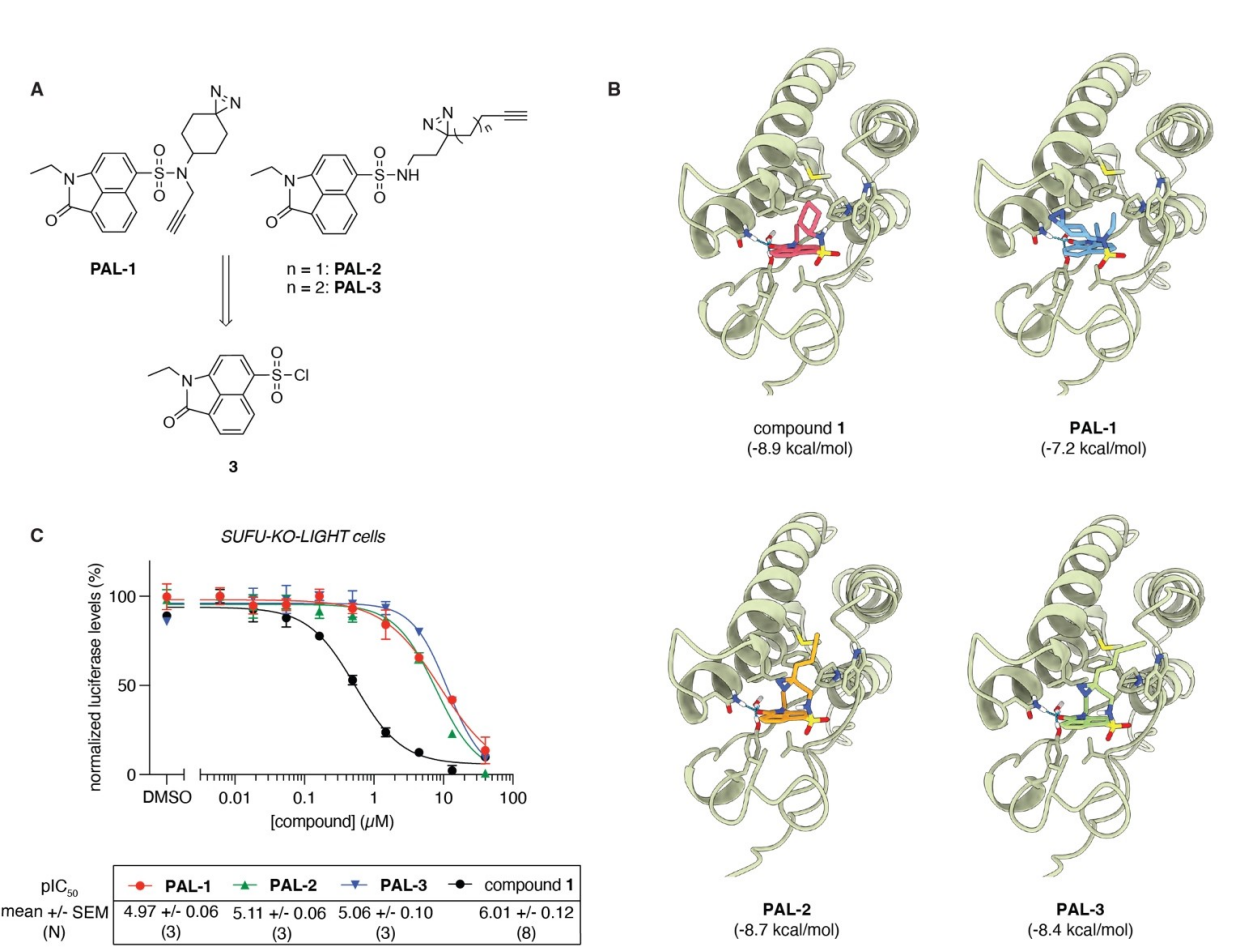
Compound **1**‐based photoaffinity probes. A) Design of **PAL1‐3** from common precursor **3**. B) Docking studies predicting a similar binding mode for **PAL1‐3** based on the reported crystal structure of compound **1** in BRD4 (PDB:5CQT). C) Biological evaluation of **PAL1‐3** in SUFU‐KO‐LIGHT cells shows a 10‐fold shift in potency for the PAL probes. The data for compound **1** is replicated from Figure 1D to allow a direct comparison. Representative graphs of N independent biological experiments are shown, performed in technical triplicates (mean ± SEM).

From these assays we could conclude that compound **1** is a legitimate downstream Hedgehog pathway inhibitor, upregulating cellular BRD2 levels. While compound **1** is known to be an *in vitro* binder of BRD2‐4, its target engagement *in cellulo* has not been demonstrated.^[19]^ To do so, we designed different photoaffinity probes based on compound **1**, employing a diazirine moiety as the photoreactive group and a propargyl group for further derivatisation and coupling with a reporter tag, as such minimizing the added bulk to the parent molecule (Figure 4A).

Previous SAR studies^[19]^ revealed higher freedom of modification on the sulfonamide group and more specifically on the N‐substituents. This led to the design of two PAL probe classes. Probe **PAL‐1**, with the two key groups separately grafted on the conserved parental structure, and the second class consisting of probes **PAL‐2** and **PAL‐3**, where the cyclohexyl group was replaced by an aliphatic chain including both the diazirine and alkyne group (Figure 4A). All three molecules could be obtained from common intermediate **3**,^[19]^ through coupling of the appropriate amines.

We carried out a series of docking calculations using the co‐crystal structure of compound **1** bound to BRD4 as a template (PDB ID: 5CQT).^[19]^ All the tested analogues are predicted to share the binding mode with the co‐crystalized ligand, in which the planar benzo[*cd*]indol‐2(1*H*)‐one core binds to the deep acetyl‐lysine binding pocket and is held there by extensive hydrophobic interactions with V87, L92, L94, and I146 (Figure 4B, Supplementary Figure 3). The carbonyl oxygen atom of the indolone interacts with the conserved N140 and forms a water‐mediated hydrogen bond with Y97. The ethyl group occupies a sub‐pocket defined by residues P82, F83, V87, and I146, mimicking the terminal methyl group of the natural substrate acetyl‐lysine. The docking scores for the PAL probes were not identical, which can be explained by the differential interactions formed by the N‐substituents of the sulfonamide group. In particular, the propargyl group of **PAL‐1** engages in hydrophobic interactions with the residues of the ^81^WPF^83^ cleft, however, its cyclohexyl‐diazirine group is unable to form specific contacts as it is not long enough to interact with the side chains of D144 or D145, which explains the lower docking score of this analogue (−7.2 kcal/mol) compared to the others. On the other hand, the diazirine linker of **PAL‐2** and the extended form present in **PAL‐3** are well accommodated in the groove defined by ^81^WPF^83^, I146 and M149, resulting in almost identical docking scores (−8.7 and −8.4 kcal/mol for **PAL‐2** and **PAL‐3**, respectively) to the parent cyclohexyl‐ containing compound (−8.9 kcal/mol). Although we cannot fully exclude the possibility of steric hindrance that could prevent the binding of our probes in a dynamic scenario, the docking calculations suggest that all the derivatives we designed could be accommodated in the binding site of BRD4 (and by extension of BRD2 and BRD3) in a similar manner to the parent compound **1**.

#### Synthesis and Evaluation of PAL1‐3 Probes

**Scheme 1:**
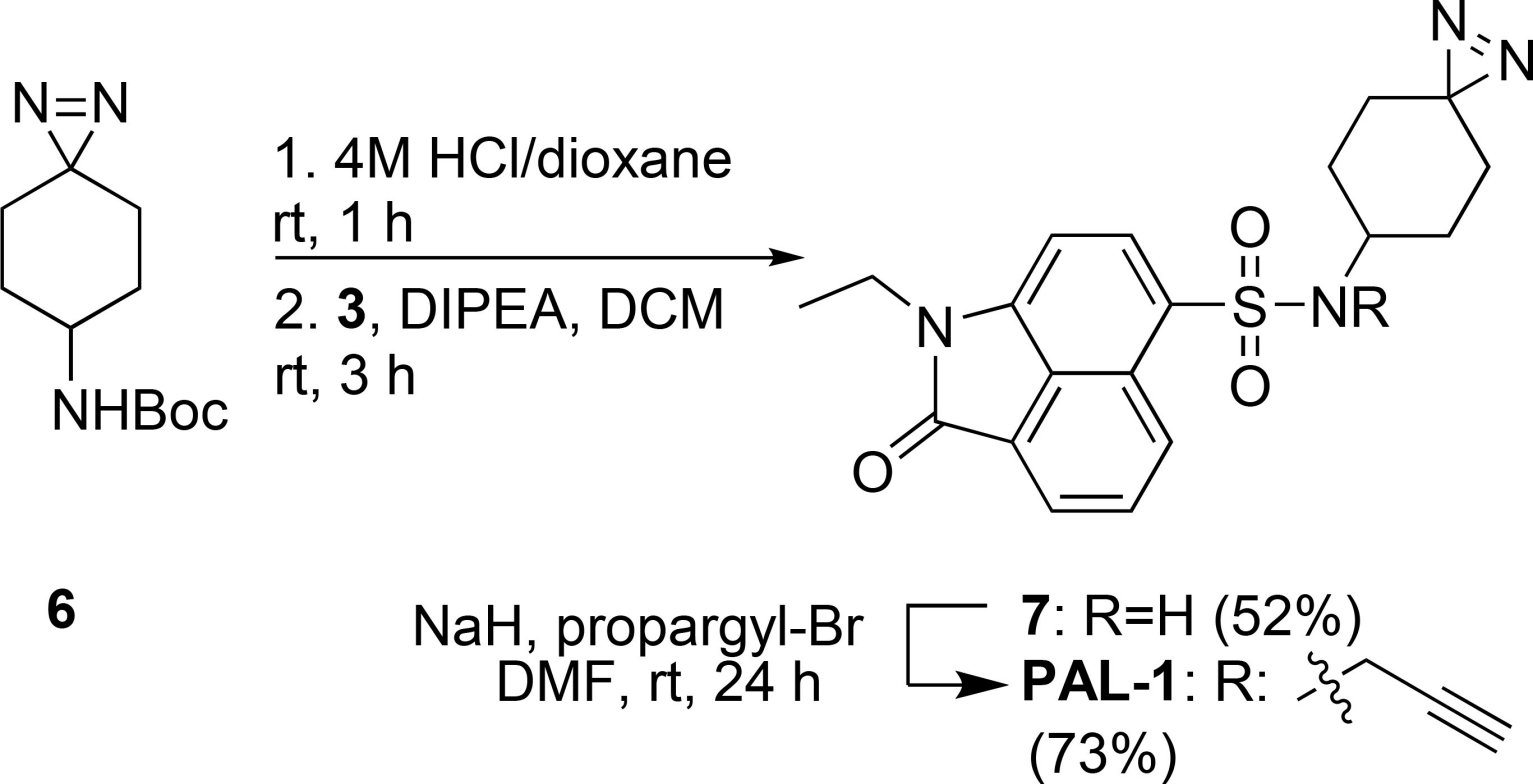
Synthesis of **PAL‐1**.

**Scheme 2:**
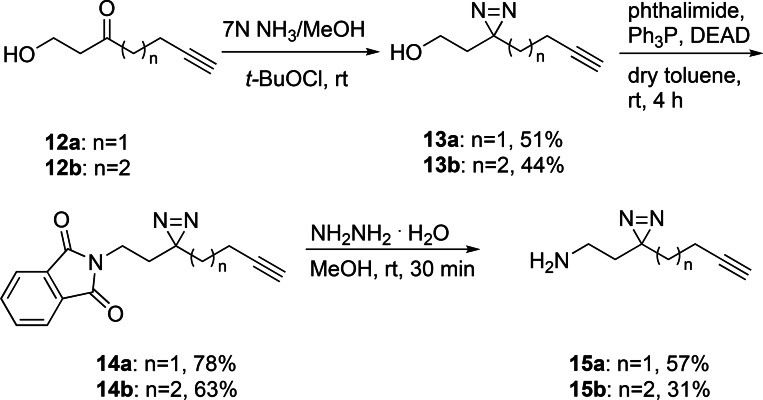
Synthesis of all‐in‐one linkers for incorporation in **PAL‐2** and **PAL‐3**.

Confident in our design, we synthesized the three different PAL probes. For **PAL‐1**, cyclohexyl‐diazirine derivative **6** was synthesized according to Wang *et al*.^[25]^ (Supplementary Scheme 1). After Boc removal and coupling with sulfonyl chloride **3**, the resulting sulfonamide **7** was N‐alkylated using propargyl bromide to introduce the bio‐orthogonal ligation handle and yield **PAL‐1** (Scheme 1). To obtain probes **PAL‐2** and **PAL‐3**, two different all‐in‐one linkers were synthesized based on the work of Shao *et al*.,^[26]^ optimizing critical steps (Scheme 2, Supplementary Scheme 2). The S_N_2 reaction between ethyl 3‐oxobutanoate and propargyl bromide resulted in the desired product **8 a**, through alkylation on the γ‐carbon of the keto‐ester (Supplementary Scheme 2A). However, the reaction did not proceed when using 4‐bromo‐1‐butyne, possibly because the stabilization of the transition state through conjugation with the neighbouring π‐system, as for the propargyl, was lost. Instead, to obtain the alkyne‐keto ester derivative **8 b**, we adapted the procedure of Kamijo *et al*.^[27]^ using vinylogous acyl triflate **9** (Supplementary Scheme 2B). The reaction proceeds through 1,2‐nucleophilic addition of the lithium enolate, generated from ethyl acetate, to the carbonyl group of **9**, followed by a Grob‐type fragmentation to cleave the C−C bond and expulsion of LiOTf to give the terminal alkyne. Subsequent ketone protection, ester hydrolysis, and deprotection gave the hydroxy‐ketones **12 a,b** (Supplementary Scheme 2C).

Synthesis of the diazirine analogue **13 a** proceeded as described (method A, Supplementary Information), in good yield (78 %). Additionally, a liquid ammonia‐free strategy was explored to introduce the diazirine in both linkers (method B, Supplementary Information), facilitating the reaction set up but decreasing the yield (44–51 %) (Scheme 2).^[28]^ To obtain final amines **15 a,b**, we envisioned a Gabriel‐like synthesis, applying Mitsunobu conditions for the generation of the phthalimide, followed by mild hydrazine‐mediated deprotection (Scheme 2).^[29]^ Amines **15 a,b** were then coupled to common intermediate **3** giving probes **PAL‐2** and **PAL‐3** (Figure 4A).

We first evaluated these probes for their activity on the Hedgehog pathway in SUFU‐KO‐LIGHT cells (Figure 4C). While the introduction of the necessary photogroup and bio‐orthogonal ligation handle did result in a ~10‐fold decrease in potency, the PAL probes were still capable to inhibit constitutive pathway activation. However, when live cells or cell lysates were treated with the probes, no distinctly labeled bands corresponding to the molecular weight of the BET bromodomain proteins were observed (Supplementary Figure 4). This could reflect a decrease in binding affinity of the probes compared to the parent compound because of the chemical modifications, a malpositioning of the photo‐reactive groups towards the protein targets, and lower cell permeability.

#### A PROTAC‐based Competition Assay Reveals BET Bromodomain Engagement in Cells

**Figure 5:**
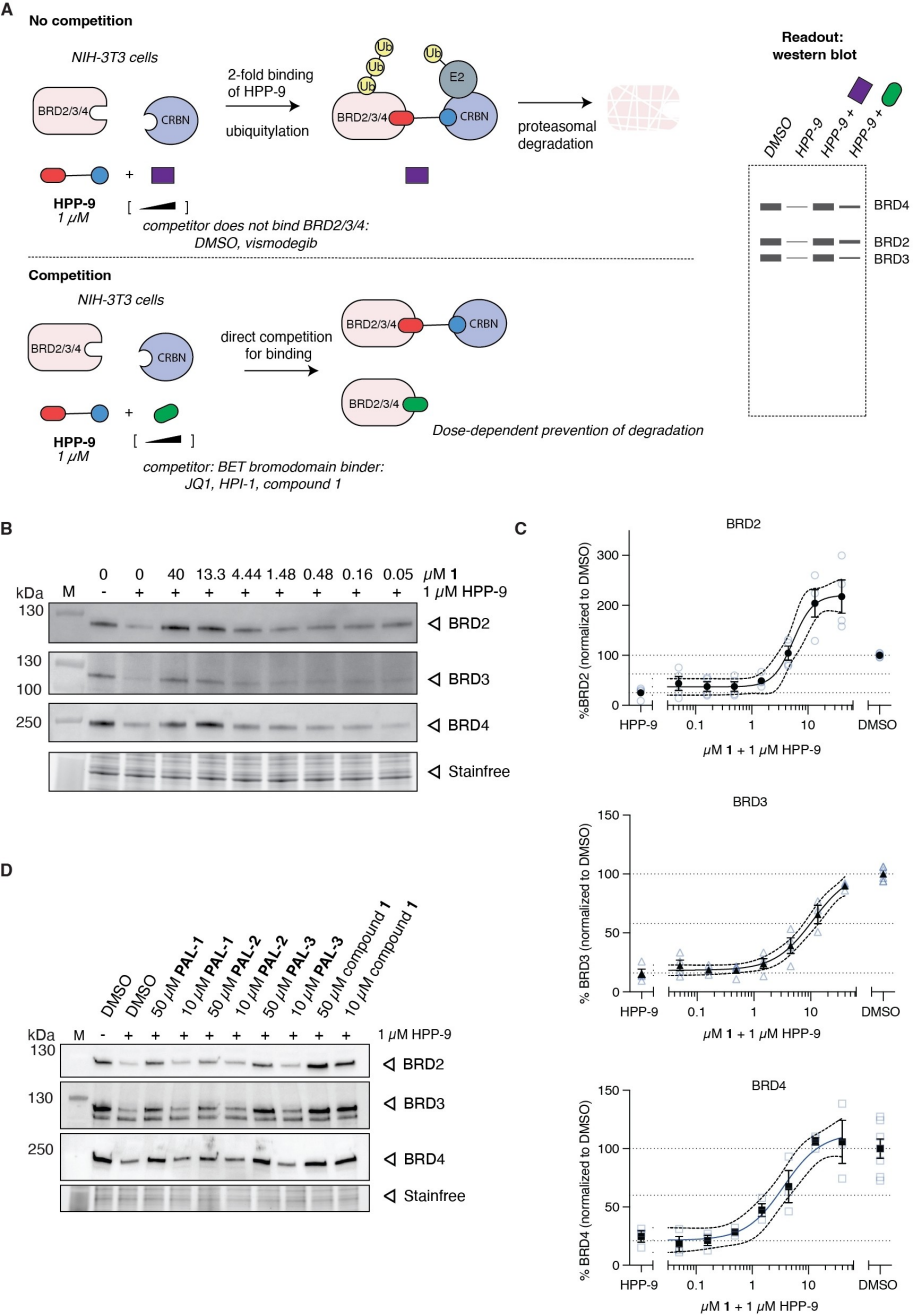
A competition assay against HPP‐9 reveals BET bromodomain engagement in cells. A) A graphical representation of the assay. B,C) NIH‐3T3 cells were co‐treated with 1 μM HPP‐9 and increasing concentration of compound **1** for 8 h. B) Representative western blots showing the dose‐dependent competition for BRD2/3/4 which is quantified in C). All bands were first corrected for protein loaded using Stainfree, before being normalized to DMSO control (100 %). Independent replicates are shown as clear blue symbols (N=4 for BRD2/3 and N=3 for BRD4; N=7 for DMSO, including additional technical replicates), whereas solid symbols with error bars present the mean of those replicates ± SEM. Curves were fitted using GraphPad Prism using the mean values, and 95 % confident intervals are shown with the dashed lines. Dotted lines present 100 % (mean of ‘DMSO’; upper line), maximum degradation by HPP‐9 (mean of ‘HPP‐9’, lower line) and the middle between these two values (50 % rescue, middle line) D) Evaluation of the PAL probes shows a competitive effect at 50 μM, but not at 10 μM. Representative blot of two independent biological experiments

To overcome the observed detrimental effects of synthetic modification on compound **1**, we envisioned a different experimental approach to directly address BET bromodomain engagement in cells. Assays of this kind are limited for BET bromodomains, as these proteins are generally important for cellular transcriptional output but lack enzymatic activity that would be amenable to direct probing. Existing biophysical approaches to assess compound binding include NanoBRET,^[30]^ which requires cell line engineering and a tracer molecule, and cellular thermal shift (CETSA) assays,^[31]^ which evaluate protein thermal stability upon compound treatment. However, since compound **1** enhanced BRD2 protein beyond baseline (Figure 3), a CETSA assay would not provide a reliable readout of compound‐protein interaction. We hypothesized that instead we could develop a competition assay against the BET bromodomain PROTAC HPP‐9 (Figure 5A).^[17]^ Previously, we have shown that HPP‐9 degrades BRD2‐4 with slow kinetics and suffers from a strong Hook effect, such that 1 μM is the most effective concentration.^[17]^ We therefore reasoned that it would be possible to outcompete this action more easily compared to competition against other, fast‐acting and more potent, BET bromodomain PROTACs such as dBet6.^[32]^

Indeed, when we co‐incubated NIH‐3T3 cells with 1 μM of HPP‐9 in the presence of varying concentrations of compound **1** or HPI‐1 as a control for 8 h, the PROTAC‐mediated degradation of all BET bromodomains was dose‐dependently inhibited (Figure 5 and Supplementary Figure 5A). As shown in Figure 5B,C and consistent with the specific effect found for BRD2 levels (Figure 3), compound **1** more potently prevented the degradation of BRD2 compared to BRD3/4 (DMSO baseline reached at 4.5 μM, compared to 13 μM for BRD4 and 40 μM for BRD3; upper dotted line), while also inducing BRD2 levels above DMSO baseline at the highest concentrations tested. For all BET proteins half of the HPP‐9 mediated degradation (Figure 5C, middle dotted line), defined as the middle between DMSO (100 %) and the maximum HPP‐9‐mediated degradation (lower dotted line), was prevented with a compound **1** concentration between 1 and 10 μM, which is in the same order of magnitude as its effect on the HH pathway. The SMO inhibitor vismodegib had no effect, as expected, whereas the BET bromodomain inhibitors HPI‐1 and JQ1 more efficiently competed with HPP‐9 (Supplementary Figure 5A,B). The differences in apparent efficiency possibly reflect the selectivity profile of these compounds for the two bromodomains of BET proteins, as compound **2** has been reported to be BD1‐selective, whereas JQ1 has high affinities for both, and HPI‐1 binds with higher affinity to BD2.[^17^, ^33^] When we repeated the competition experiment with the various PAL probes, competition was much less effective, in line with their reduced potency as HH pathway inhibitors and inability to label the BET bromodomains (Figure 5D). Interestingly, in contrast to work by Jiang *et al*. on a PROTAC based on reversed sulfonamide benzo[*cd*]indol‐2(1*H*)‐one **2**, we did not find pronounced selectivity of compound **2** for BRD2/4 over BRD3 in this competition assay (Supplementary Figure 5C).^[33]^ This could reflect insufficient resolution in our assay, or it could suggest that the selectivity arises not from the core, but from the position of the exit vector in the PROTAC, or it reflects a cell‐type specific effect. Taken together, this competition assay presents a low cost and easy to implement alternative to CETSA or NanoBRET assays. While readout by western blot is low throughput and has limited quantitative accuracy, we envision that this assay could be developed into a microscopy‐based multiplexed assay, allowing high‐content screening of potential BET bromodomain binders. A limitation is the event‐driven irreversible nature of the readout, similar to when using covalent activity‐based probes in competition with non‐covalent inhibitors. The amount of degradation and the level of competition thus strongly depend on the time of the assay.

#### Compound 1 Reduces Cell Viability of HH Pathway‐Driven Cancer Cell Lines

**Figure 6:**
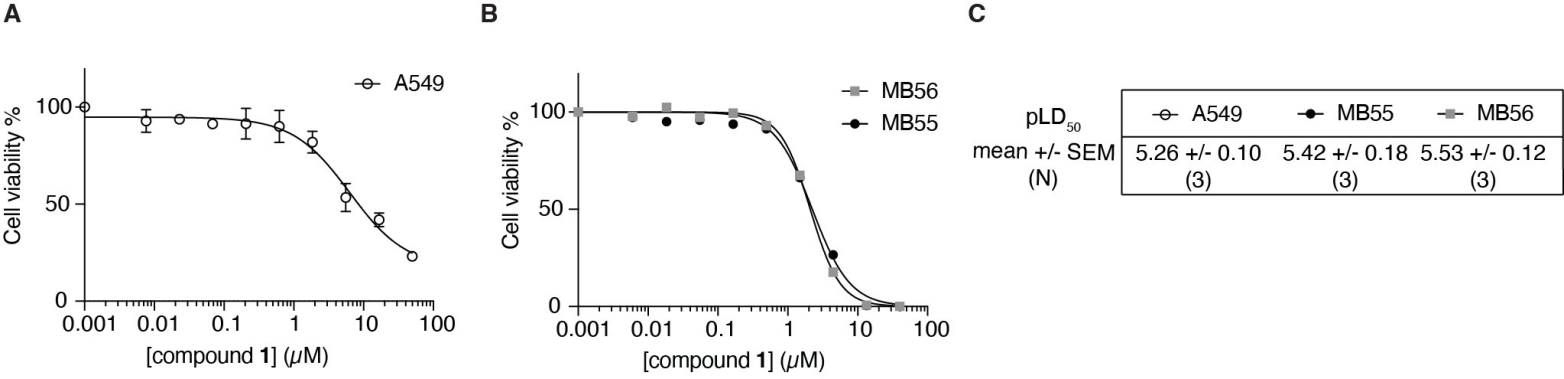
Compound **1** dose‐dependently kills HH pathway‐dependent cancer cell lines. A) Cell toxicity of compound **1** in A549 cells, and B) in medulloblastoma spheroid cells MB55 and MB56. C) Quantification of 3 independent biological experiments.

Having established the in‐cell engagement of BET bromodomains by compound **1**, and its subsequent inhibitory effects on Hedgehog signal transduction, we wanted to investigate whether this compound could be beneficial against HH pathway‐driven cancers. For this, we assessed the cytotoxicity of compound **1** against GLI‐driven A549 lung carcinoma cells and medulloblastoma MB55 and MB56 spheroids (Figure 6).^[34]^ In both cases, we found that this compound was able to kill these cells with LD_50_ values ranging between 1–10 μM, consistent with its inhibitory potency of the Hedgehog pathway. These potencies are also very similar to what has been previously reported by Xue *et al*., for the effect of compound **1** against other types of cancers (MV4;11: 3.5 μM, HL‐60: 2 μM, HT‐29: 8.5 μM).^[19]^ Overall, this illustrates the potential pharmacological utility of the benzo[*cd*]indol‐2(1*H*)‐one scaffold against a broad range of cancer cell lines that depend on BET proteins for their proliferation, including those initiated by HH pathway activation.

## Conclusions

Epigenetic targeting of the HH pathway through modulation of BET bromodomains is an attractive strategy towards combating HH pathway‐driven cancers. Here, we validate the inhibitory activity of benzo[*cd*]indol‐2(1*H*)‐one **1**, a hit from a phenotypic screen geared towards the discovery of downstream HH pathway inhibitors. Compound **1** inhibits the HH pathway in a variety of cell types, and under varying conditions of pathway activation, with submicromolar activity. We designed and synthesized three PAL probes based on compound **1**, using an optimized synthetic methodology towards the efficient synthesis of all‐in‐one linkers for photoaffinity labeling experiments. While labeling of BET bromodomains with these probes did not succeed, we could show that this compound engages BET bromodomain proteins in cells through a competition assay with a BET bromodomain‐targeting PROTAC. Finally, we demonstrate the cytotoxicity of this compound against different cancer cell lines that are dependent on HH signaling for their survival, adding benzo[*cd*]indol‐2(1*H*)‐ones to the growing set of BET bromodomain inhibitors that are effective downstream HH pathway inhibitors.

## Supporting Information

Full experimental procedures and analytical data can be found in the Supporting Information. The authors have cited additional references within the Supporting Information.[^35^, ^36^, ^37^, ^38^, ^39^]

## Conflict of Interests

The authors declare no conflict of interest.

1

## Supporting information

As a service to our authors and readers, this journal provides supporting information supplied by the authors. Such materials are peer reviewed and may be re‐organized for online delivery, but are not copy‐edited or typeset. Technical support issues arising from supporting information (other than missing files) should be addressed to the authors.

Supporting Information

## Data Availability

The data that support the findings of this study are openly available in Zenodo at 10.5281/zenodo.14383588.
