# Supplementary material for: Evaluation of Benzo[cd]indol‐2(1H)‐ones as Downstream Hedgehog Pathway Inhibitors
Source: ChemistryOpen. 2025 Apr 14;14(5):e202500119. doi: 10.1002/open.202500119 (PMC12075100; doi:10.1002/open.202500119)

ChemistryOpen

Supporting Information

Evaluation of Benzo[*cd*]indol-2(1*H*)-ones as Downstream Hedgehog Pathway Inhibitors

Ioannis A. Tsakoumagkos, Quentin T. L. Pasquer, Christian-Louis Guillod, Charlotte Rossion, Meropi Bagka, Sonya Torche, Tomoyo Sakata-Kato, James K. Chen, and Sascha Hoogendoorn*

Evaluation of Benzo[*cd*]indol-2(1*H*)-ones as Downstream Hedgehog Pathway Inhibitors

Ioannis A. Tsakoumagkos,[a] Quentin T. L. Pasquer,[a] Christian-Louis Guillod,[a] Charlotte Rossion,[a] Meropi Bagka,[a] Sonya Torche,[a] Tomoyo Sakata-Kato,[b],[e] James K. Chen,[b],[c],[d] Sascha Hoogendoorn*[a]

Supporting Information

Table of Contents

1. SUPPLEMENTARY FIGURES AND SCHEMES 2

2. METHODS 8

2.1 Biology 8
2.1.1 High-throughput screen for small-molecule antagonists of GLI function 8
2.1.2 Cell lines and maintenance. 8
2.1.3 ShhN and Wnt production and titering. 9
2.1.4 Luciferase reporter assays. 9
2.1.5 Alkaline phosphatase assay. 11
2.1.6 Cell viability assays. 11
2.1.7 Docking calculations. 12
2.1.8 Immunofluorescence imaging. 12
2.1.9 Quantification of fluorescence microscopy images. 13
2.1.10 Photo-affinity labeling. 13
2.1.11 Western blotting. 14

2.2 Synthesis 15
2.2.1 General. 15
2.2.2 Experimental procedures. 16

3. REFERENCES 28

4. NMR SPECTRA 29

1. Supplementary Figures and Schemes

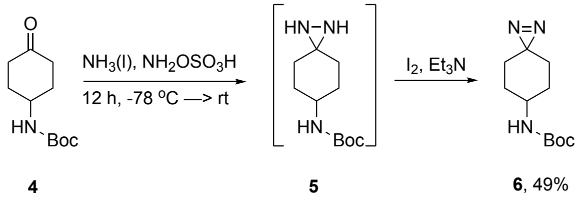

Supplementary Scheme 1. Synthesis of cyclohexyl diazirine **6**.

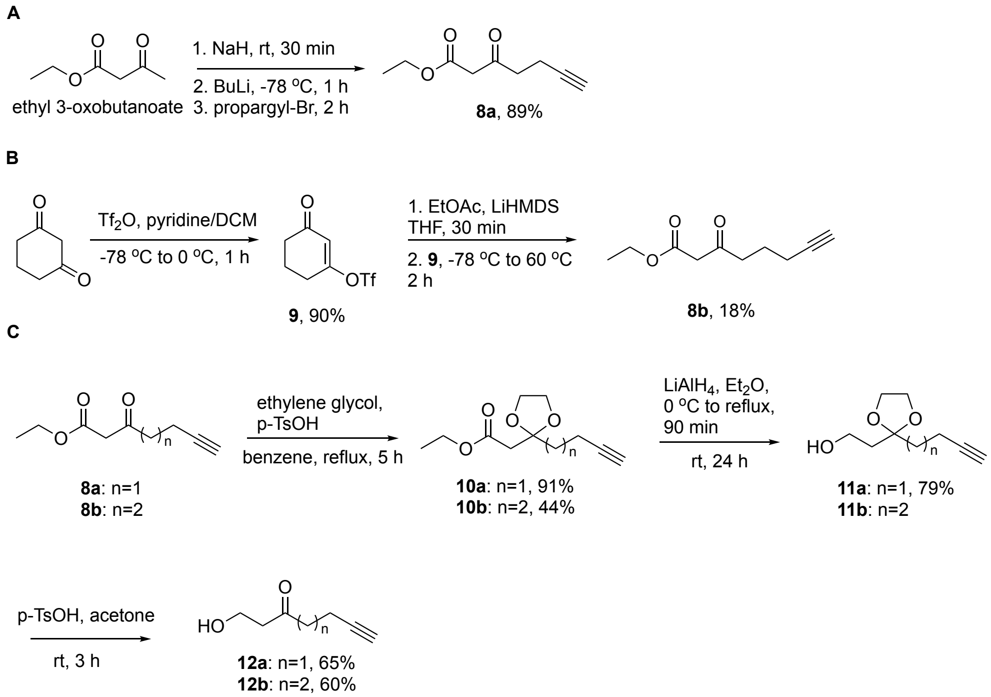

Supplementary Scheme 2. Synthesis of hydroxyketone linkers.

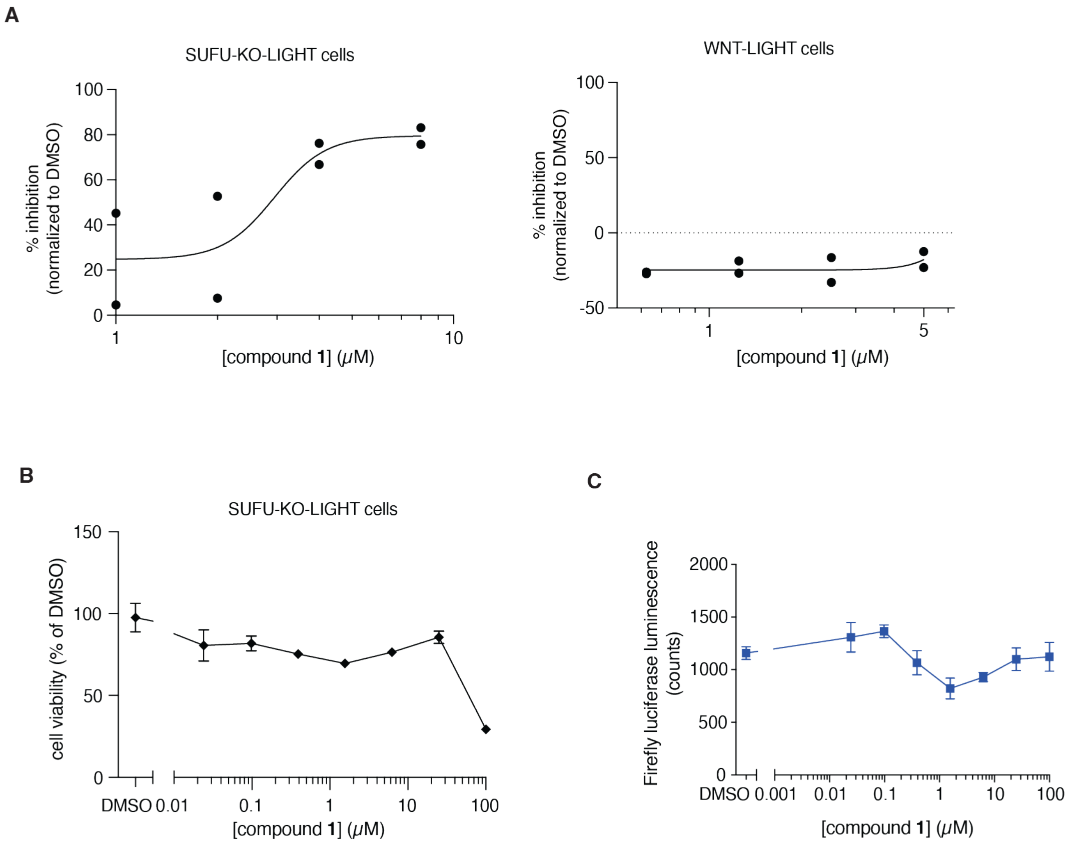

Supplementary Figure 1. A) Primary screen data showing the inhibitory potency of compound **1** in SUFU-KO-LIGHT cells, but not WNT-LIGHT cells. Four doses were tested in duplicate. Secondary screens showed that compound **1** B) does not affect cell viability in SUFO-KO MEFs up until 20 μM, but shows toxicity at higher concentrations, and C) does not inhibit constitutively expressed firefly luciferase. Mean +/- SEM of technical triplicates is shown.

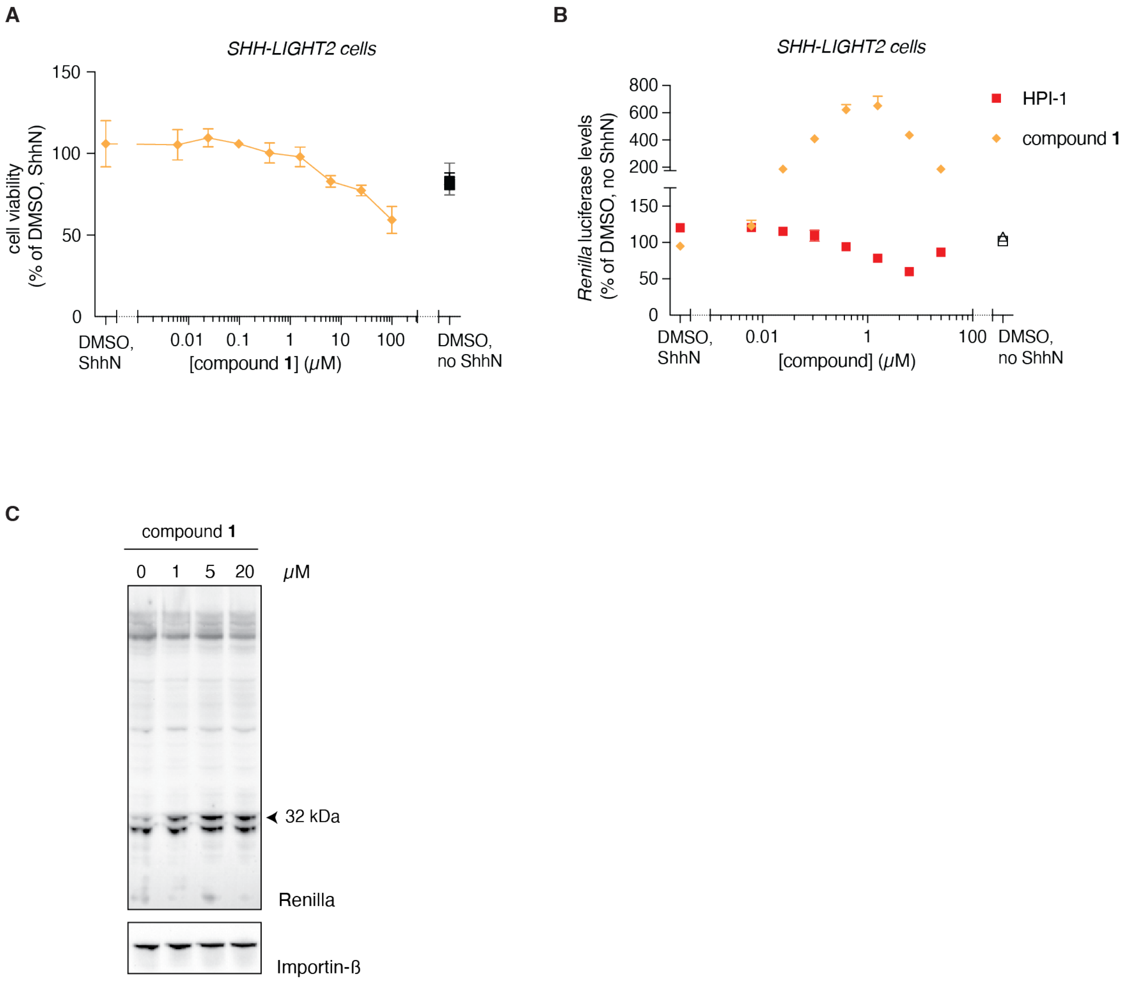

Supplementary Figure 2. A) Compound **1** does not affect cell viability of SHH-LIGHT2 cells at relevant concentrations, as measured using CellTiter 96. Representative curve of N=7 independent biological experiments is shown, mean of 3 technical triplicates +/- SEM. B) Compound **1**, but not HPI-1, dose-dependently increases *Renilla* luciferase luminescence in SHH-LIGHT2 cells. Representative curves of N=7 independent biological experiments performed in technical triplicates are shown. C) Representative immunoblot shows that the levels of *Renilla* protein are increased by compound **1** treatment.

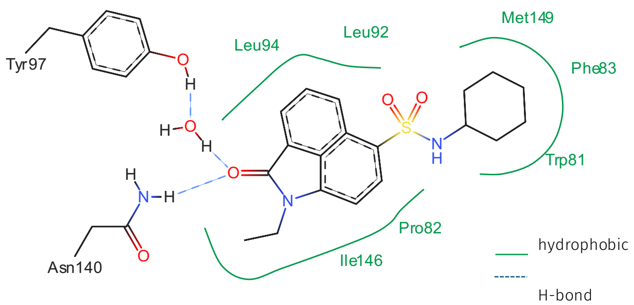

Compound 1

(-8.9 kcal/mol)

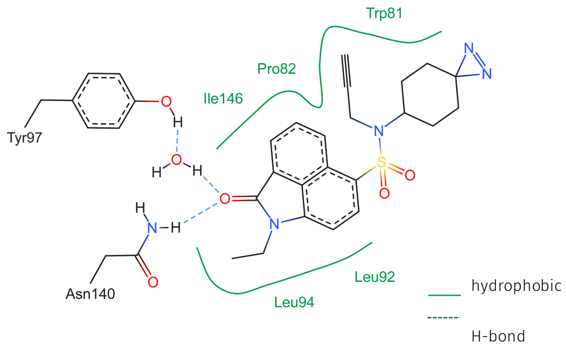

PAL-1

(-7.2 kcal/mol)

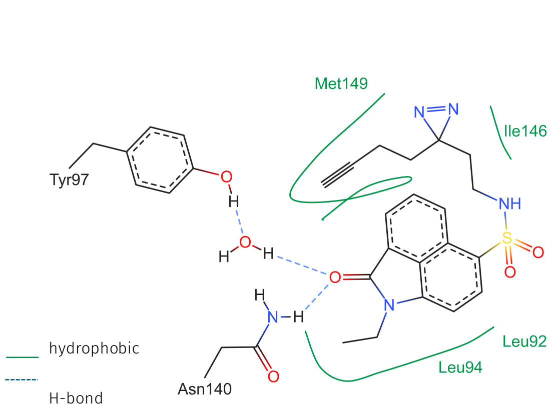

PAL-2

(-8.7 kcal/mol)

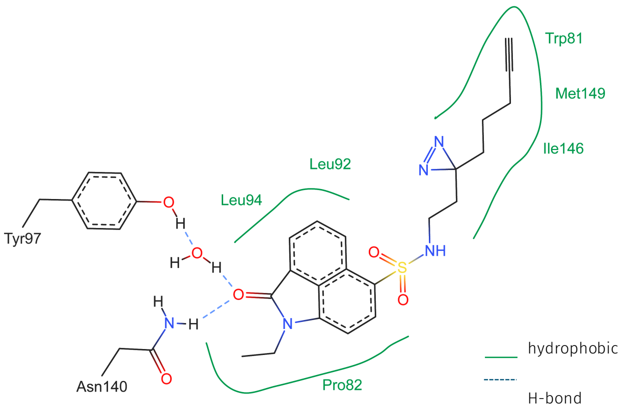

PAL-3

(-8.4 kcal/mol)

Supplementary Figure 3. 2D representation of the binding mode of compound **1** and **PAL1-3** as shown in Figure 4B.

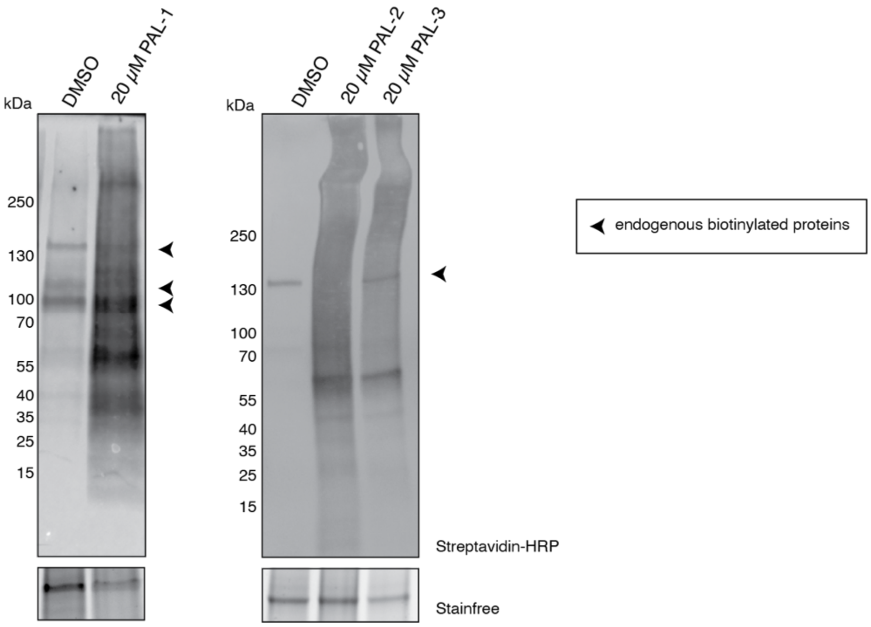

Supplementary Figure 4. Representative immunoblots of photo-affinity labeling experiments in NIH-3T3 lysates. Lysates were treated with 20 μM of PAL probes, irradiated and subjected to click chemistry to attach biotin. Labeled proteins were detected using Streptavidin-HRP and total protein using Stainfree.

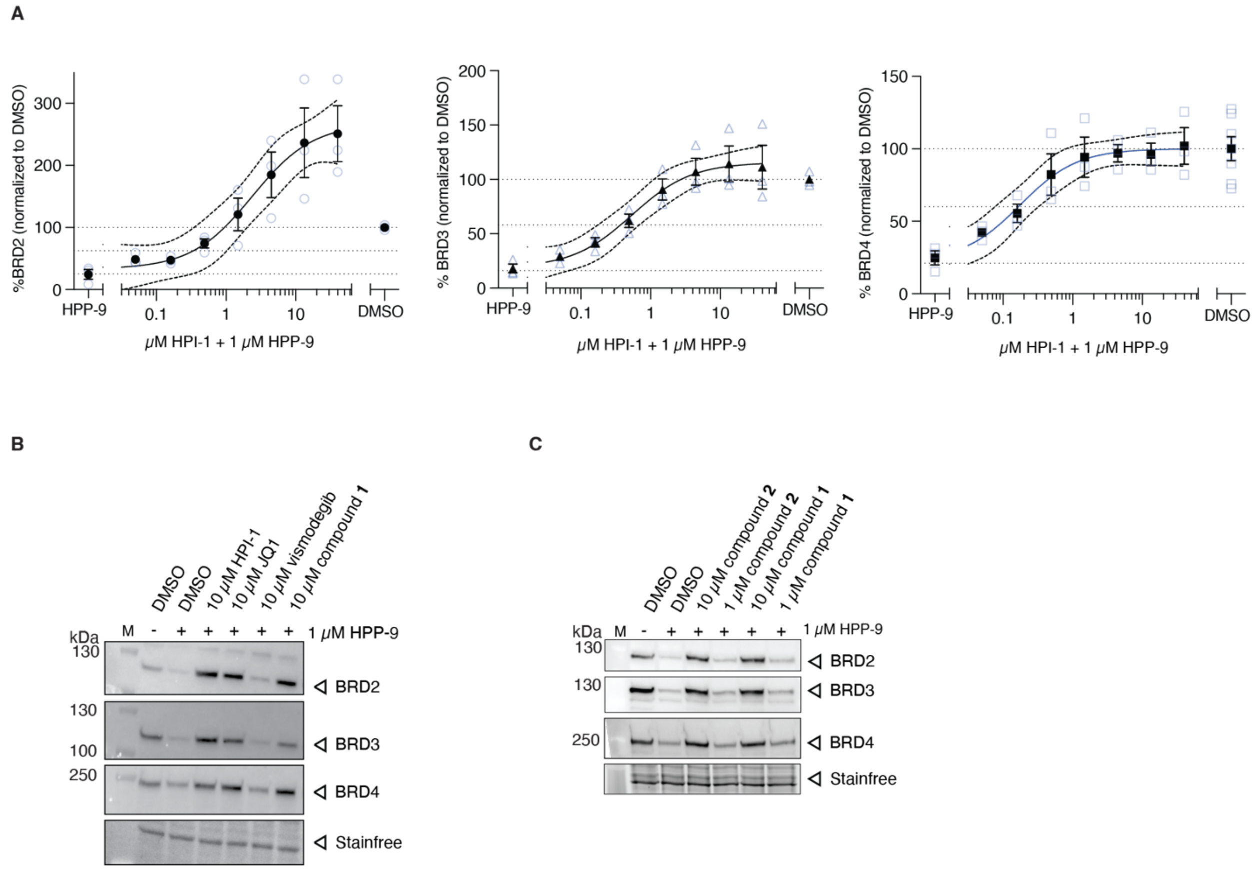

Supplementary Figure 5. A) HPI-1 dose-dependently outcompetes the degradation of BET bromodomain proteins by HPP-9. Western blot quantification of three independent experiments is shown. All bands were first corrected for protein loaded using Stainfree, before being normalized to DMSO control (100%). Independent replicates (N=3) are shown as clear blue symbols, whereas solid symbols with error bars present the mean +/- SEM. DMSO (N=7) and HPP-9 values are the same as those shown in Figure 5C. Curves were fitted using GraphPad Prism using the mean values, and 95% confident intervals are shown with the grey dashed lines. Dotted lines present 100% (DMSO; upper line), maximum degradation by HPP-9 (HPP-9, lower line) and the middle between these two values (50% rescue, middle line). B) Representative immunoblot of duplicate experiments showing that JQ1, but not vismodegib can outcompete the action of HPP-9. C) Representative immunoblot of duplicate experiments showing that compound **2** outcompetes HPP-9 action on all three BRD proteins, without apparent selectivity.

2. Methods

2.1 Biology

2.1.1 High-throughput screen for small-molecule antagonists of GLI function[1]

The high-throughput chemical screen for inhibitor of GLI function was contracted to the Conrad Prebys Center for Chemical Genomics at the Sanford Burnham Prebys Medical Discovery Institute. The screen conditions were equivalent to those described in PubChem AID 588413. In brief, 5 x 10^3 SUFU-KO-LIGHT cells were seeded into white 1536-well plates. 16 to 18 hours after seeding, the cells were treated with individual compounds at the final concentration of 8 or 5 µM (confirmation screen; relevant description, protocol and scoring rules in PubChem AID 602464) and cultured for another 16 to 18 hours. The cells were then treated with Bright-Glo luciferase substrate (3 µL/well; Promega) for 10 minutes at room temperature before luminescence was read on a Viewlux microplate imager. Hits with at least 50% luciferase inhibition were expanded for a dose-response assay, and those with IC_{50}s lower than 20 µM were subsequently counter-screened in cytotoxicity (relevant description, protocol and scoring rules in PubChem AID 602464), constitutive firefly luciferase, and WNT assays (relevant description, protocol and scoring rules in PubChem AID 651570).

2.1.2 Cell lines and maintenance. SHH-LIGHT2[2] cells were maintained in DMEM containing 10% calf serum (CS), 1% sodium pyruvate, 100 U/mL penicillin, 100 µg/mL streptomycin, 150 µg/mL zeocin and 400 µg/mL G418. SHH-GFP cells[3] were maintained in DMEM containing 10% CS, 1% sodium pyruvate, 100 U/mL penicillin, 100 µg/mL streptomycin and 150 µg/mL zeocin. SUFU-KO-LIGHT[1] and WNT-LIGHT[1] were maintained in DMEM containing 10% fetal bovine serum (FBS), 1% sodium pyruvate, 100 U/mL penicillin, 100 µg/mL streptomycin and 150 µg/mL zeocin. A549 (ATCC, CCL-185), Wnt3a-producing L cells (ATCC, CRL-2647), C3H10T1/2 (ATCC, CCL-226), and HEK239T-EcR-ShhN cells (provided by Phil Beachy, Stanford University) were maintained in DMEM containing 10% FBS, 100 U/mL penicillin and 100 µg/mL streptomycin. TM3-LIGHT[4] cells were cultured in F12 Ham's/DMEM (1:1) containing 5% horse serum, 2.5% FBS and 15 mM HEPES, pH 7.3. MB55 and MB56[5] were a gift from Rosalind Segal, Harvard Medical School and were cultured as spheroids in

1:1 DMEM:F12, B27 supplement (Gibco), 100 U/mL penicillin and 100 µg/mL streptomycin. All cells were cultured at 37 °C with 5% CO_2.

2.1.3 ShhN and Wnt production and titering. HEK239T-EcR-ShhN cells were grown to 80% confluence. Subsequently, the medium was changed to 2% FBS DMEM, followed by collection of the conditioned medium after 48 h. The medium was filtered through a 0.22 µm filter device (Corning) and stored at -20°C. To produce Wnt-conditioned medium, Wnt3A expressing L cells were grown to 70% confluence, and the medium changed to 10% FBS DMEM. The conditioned medium was collected after 48 h, filtered, and stored for a maximum of 4 weeks at 4°C. Titers were determined using SHH-LIGHT2 or WNT-LIGHT luciferase reporter cells (see *Luciferase reporter assays*). A concentration approximately two-fold over the minimum dilution needed for full luciferase induction was used for further experiments (typically between 1:5 and 1:15 depending on the batch).

2.1.4 Luciferase reporter assays.

CMV-luciferase assay. NIH-3T3 cells were seeded into a 24-well plate (35.000 cells/well). After 24 hours, the cells were approximately 50% confluent and co-transfected with pRLSV40 (5 ng/well), a CMV-driven firefly luciferase expression vector (15 ng/well), using TransIT-LT1 reagent (Mirus Bio). The medium was changed the following day, and the cells were cultured for an additional 24 hours until they reached 100% confluency. The cells were then cultured in serum starvation medium (DMEM w/o phenol red, 0.5% FBS) containing the indicated compound concentration for another 24 hours. Cells were then lysed using the Dual Luciferase kit (Promega), and firefly and *Renilla* luciferase activities were measured on a Veritas luminometer.

SUFO-KO-LIGHT assay. SUFU-KO-LIGHT cells were seeded in a 96 well plate (30.000 cells/well). The next day, the medium was removed and a serial dilution of compound in starvation medium (DMEM w/o phenol red, 0.5% FBS) or an equivalent amount of DMSO vehicle was added and the cells incubated for 16-18 h. CellTiter AQueous One (Promega) was then added to the cells and incubated for 1 h at 37 °C. Cell viability was measured as absorbance at 490 nm of the culture media on a Glomax multi-mode microplate reader (Promega). Next, the medium was removed and the cells were lysed for 30 min at room temperature (12.2 mM Tris pH 7.4, 4% glycerol, 0.5% Triton X-100, 0.5 mg/mL BSA, 1 mM EGTA, 1 mM DTT). Homemade firefly luciferase reagent (0.025 M di-glycine, 0.015 M $KxPO_4$ pH=8, 4 mM EGTA pH=8, 0.5 mM DTT, 0.015 M $MgSO_4$, 2 mM ATP, 25 mM Coenzyme A, and

0.9 µM luciferin) was added, and the luminescent signal measured on a Glomax multi-mode microplate reader (Promega). The data was normalized to DMSO control and curves were fitted and analyzed to determine the IC_{50} values using GraphPad (Prism 9, GraphPad Software, La Jolla, CA).

SHH-LIGHT2 assay. SHH-LIGHT2 cells were seeded in a 96 well plate (35.000 cells/well). The next day, the medium was removed and a serial dilution of compound in ShhN-containing or SAG (200 nM)-containing starvation medium (DMEM w/o phenol red, 0.5% CS) or an equivalent amount of DMSO vehicle was added and the cells incubated for 28 h. 20 µL/well CellTiter AQueous One (Promega) was then added to the cells and incubated for 1 h at 37 °C. Cell viability was measured as absorbance at 490 nm of the culture media on a Tecan M1000Pro. Cells were then washed with PBS and lysed in 50 µL passive lysis buffer from the Dual Luciferase kit (Promega). Gli-dependent firefly luciferase activity was measured on a Veritas luminometer and normalized to the CellTiter signal. Data was normalized to the DMSO with or without ShhN/SAG controls and curves were fitted and analyzed to determine the IC_{50} values using GraphPad (Prism 9, GraphPad Software, La Jolla, CA).

TM3-LIGHT assay. TM3-LIGHT cells were seeded in a 96 well plate (35.000 cells/well). The next day, the medium was removed and a serial dilution of compound in ShhN-containing or SAG (200 nM)-containing starvation medium (1:1 DMEM:F12 w/o phenol red, 0.4% horse serum, 0.1% FBS, 1.2 g/L $NaHCO_3$, 1% sodium pyruvate, 100 U/mL penicillin, 100 µg/mL streptomycin) or an equivalent amount of DMSO vehicle was added and the cells incubated for 30 h. 5 µL/well CellTiter AQueous One (Promega) was then added to the cells and incubated for 20 minutes at 37 °C. Cell viability was measured as absorbance at 490 nm of the culture media on a Tecan M1000Pro. Cells were then washed with PBS and 50 µL PBS followed by 25 µL BrightGlo reagent (Promega) was added, and cells incubated for 5 minutes at room temperature. Gli-dependent firefly luciferase activity was measured on a Veritas luminometer and normalized to the CellTiter signal. Data was normalized to the DMSO with or without ShhN/SAG controls and curves were fitted and analyzed to determine the IC_{50} values using GraphPad (Prism 9, GraphPad Software, La Jolla, CA).

WNT-LIGHT assay. WNT-LIGHT cells were seeded in a 96 well plate (10.000 cells/well). The next day at 40% confluency, the medium was removed and Wnt-containing starvation medium (DMEM w/o phenol red, 0.5% FBS) with compounds in the indicated concentrations or an equivalent amount of DMSO vehicle was added and the cells incubated for 28 h. 20 µL/well CellTiter AQueous One

(Promega) was then added to the cells and incubated for 1.5 h at 37 °C. Cell viability was measured as absorbance at 490 nm of the culture media on a Tecan M1000Pro. Cells were then washed with PBS and lysed in 50 µL passive lysis buffer from the Dual Luciferase kit (Promega), and TCF/LEF-dependent firefly luciferase activity was measured on a Veritas luminometer and normalized to CellTiter levels.

2.1.5 Alkaline phosphatase assay. C3H10T1/2 cells were seeded into a 96-well plate (20.000 cells/well). The next day, the medium was removed and a serial dilution of compound in ShhN-containing or SAG (200 nM)-containing starvation medium (DMEM w/o phenol red, 0.5% CS) or an equivalent amount of DMSO vehicle was added and the cells incubated for 48 h. 20 µL/well CellTiter AQueous One (Promega) was then added to the cells and incubated for 30 minutes at 37 °C. Cell viability was measured as absorbance at 490 nm of the culture media on a Tecan M1000Pro. Cells were then washed with PBS and lysed in 50 µL lysis buffer (100 mM Tris- HCl, pH 9.5, 250 mM NaCl, 25 mM MgCl2, and 1% Triton X-100) for 45 minutes, while rocking. 10 µL of lysate was transferred to a white assay plate and 50 µL Tropix CDP-Star Chemiluminescence reagent (Applied Biosciences) was added and incubated for 30 minutes in the dark. Luminescence was measured on a Veritas luminometer and normalized to the CellTiter signal. Data was normalized to the DMSO with or without ShhN/SAG controls and curves were fitted and analyzed to determine the IC_{50} values using GraphPad (Prism 9, GraphPad Software, La Jolla, CA).

2.1.6 Cell viability assays. A549 cells were seeded in a 96 well plate (3000 cells/well). The next day, the cells were treated with the indicated compound concentrations or DMSO vehicle and incubated for 48 h. MB55 and MB56 cells were seeded in a low adherent 96 well plate (10.000 cells/well). After 24 h, the cells were treated with the indicated compound concentrations or DMSO vehicle and incubated for 168 h. Cell viability was determined using Celltiter-Blue (Promega) and fluorescence measured using a multi-mode microplate reader GloMax (Promega) using excitation and emission wavelengths of 570 nm and 600 nm, respectively, or using Celltiter-Glo 3D (Promega) and measuring luminescence (for spheroids). The results were analyzed using a non-linear regression (Prism 9, GraphPad Software, La Jolla, CA).

2.1.7 Docking calculations.

Prior to any docking calculation, we prepared the crystal structure of the human bromodomain BRD4 in complex with benzo[*cd*]indol-2(1*H*)-one ligand (PDB ID 5CQT[6]) using the Protein Preparation Wizard of the Schrödinger suite to add bond orders and formal charges to the starting protein structure.[7] The protonation state of each residue was determined using the PROPKA algorithm at pH 7.4, as implemented in Maestro. All crystallographic water molecules were removed except for the one that mediates an interaction between Y97 and the carbonyl group of benzo[*cd*]indol-2(1*H*)-one. The structure of each protein was relaxed prior to docking using the OPLS4 force field.[8] To dock the benzo[*cd*]indol-2(1*H*)-one and its derivatives to BRD4, we used the single precision (SP) scoring function and docking protocol of Glide 7.6 (as implemented in the Schrödinger suite).[9] The ability of our docking protocol to reproduce the crystallographic pose of benzo[*cd*]indol-2(1*H*)-one, as seen in the crystal structure (RMSD<1 Å), gives us confidence in the docking results of the derivatives. From the different docked poses we obtained for each ligand, the choice of the final pose we presented here was based solely on the docking score of each pose.

2.1.8 Immunofluorescence imaging.

For GLI2/GLI3 trafficking studies, SHH-GFP cells were grown to confluency, serum-starved (DMEM w/o phenol red, 0.5% CS) for 20 h to induce ciliogenesis, followed by 6 h incubation with 10 μM compound **1** or DMSO control in the presence or absence of ShhN-conditioned as indicated. Cells were sequentially fixed with 4% PFA for 10 minutes and ice-cold methanol for 5 minutes, before being washed with PBS (3x), and blocked in blocking solution (1% BSA in PBS-T) for 1 h at room temperature. Cells were incubated with primary antibody in blocking solution (1:500 mouse anti-gamma tubulin (Sigma Aldrich, GTU-88), 1:3000 mouse anti-ARL13B (Biolegend, clone N295B/66), 1:500 Goat anti-mouse GLI2 (R&D systems, AF3635), 1:500 Goat anti-mouse GLI3 (R&D systems, AF3690)) overnight at 4 °C, washed 5 x 5 minutes with PBS-T, and incubated with appropriate secondary antibodies in blocking solution (1:500 Jackson ImmunoResearch) for 1 h at room temperature. Cells were washed 5 x 5 minutes with PBS-T and once with PBS before being imaged using a 40x water immersion objective on a high content confocal microscope (Molecular Devices™ ImageXpress Micro XL).

2.1.9 Quantification of fluorescence microscopy images.

Image analysis was performed using a custom Matlab (Mathworks) script.[10] Local background subtraction was performed on all images before analysis. To determine GLI levels at the tip of the primary cilium, the ARL13B channel was used to create a ciliary mask. The ciliary mask was then used to identify and measure ciliary signal in the other channels. The γ-tubulin signal (as a centriole marker) was used to orient all cilia from base to tip. Each cilium was divided in 10 bins, and the tip fluorescence for GLI2 and GLI3 was defined as the summed fluorescence in the final four bins of each cilium, regardless of length.

2.1.10 Photo-affinity labeling.

SHH-GFP cells were seeded to reach confluency by the next day. To generate protein lysates, cells were washed twice with PBS, harvested with 1X trypsin or by use of a cell scraper, and collected by centrifugation. Cell pellets were then washed with PBS and resuspended in PBS buffer (with cOmplete™, EDTA-free protease inhibitor cocktail and Benzonase® nuclease (Millipore #70746) 25kU/mL)) and incubated for 10 minutes at 20°C–25°C with shaking until viscous samples became clear. The supernatant was isolated by centrifugation at 5000 x g for 10 minutes at 4°C for subsequent steps. Cell pellets were lysed at 4°C with probe sonication using a Fisherbrand Model 120 Sonic Dismembrator with the following settings: 1 second on, 1 second off pulses for 20 seconds at 75% amplitude. Supernatant was isolated after centrifugation. Protein concentration was determined by Pierce™ BCA Protein Assay Kit (Thermo Scientific™, #23252). Cell lysates were treated with probes in different concentrations or with an equivalent amount of DMSO and incubated for 2 h at room temperature. Samples were irradiated for 20 minutes on ice (CL-1000 Ultraviolet Crosslinker 368nm). Next, a click chemistry master mix was added to each sample (final concentrations: 0.1 mM TBTA, 1 mM $CuSO_4$, 1 mM TCEP, 25 μM biotin-azide) and incubated for 1 h at room temperature. Proteins were precipitated in cold acetone for 24 h at 4°C, collected by centrifugation and re-solubilized in urea buffer (8 M urea, 50 mM Tris HCl pH 8). SDS sample buffer (50 mM Tris HCl pH 6.8, 8% v/v glycerol, 2% w/v SDS, 100 mM DTT, 0.1mg/mL bromophenol blue) was added and lysates were boiled at 95°C for 10 minutes and sonicated for a few seconds. Proteins were resolved as described in the next section.

2.1.11 Western blotting.

SHH-GFP cells were seeded in a 24-well plate and grown until confluency. Growth medium was then replaced with serum starvation medium (DMEM w/o phenol red, 0.5% CS) with or without ShhN conditioned medium and compounds at the appropriate dilution. After 28 h, cells were lysed in SDS sample buffer (50 mM Tris HCl pH 6.8, 8% v/v glycerol, 2% w/v SDS, 100 mM DTT, 0.1 mg/mL bromophenol blue), boiled and sonicated. Lysates were either probed for signaling components (GLI1/GLI2/GLI3) or BET bromodomains (BRD2/BRD3/BRD4). Total protein loading was either determined using Stainfree signal or by probing the housekeeping protein Importin beta-1 (KPNB1).

For competition assays, SHH-GFP cells were seeded in a 24-well plate (130.000 cells/well). The next day, medium was changed to starvation medium (DMEM w/o phenol red, 0.5% CS) with 1 µM HPP-9 or DMSO in competition with compounds at the indicated concentrations. After 8 hours of treatment, cells were lysed in SDS sample buffer, boiled, and sonicated.

For Renilla expression levels, SHH-LIGHT2 cells were seeded in a 24-well plate and grown until confluency. Growth medium was then replaced with compound at the appropriate dilution or DMSO control in serum starvation medium (DMEM w/o phenol red, 0.5% CS). After 28 h, cells were lysed in SDS sample buffer, boiled and sonicated.

Samples were loaded onto a 4-15% Criterion TGX Stainfree gel (Bio-Rad), and run for 45 min, 200V in Tris/Glycine/SDS buffer (Bio-Rad). Gels were irradiated (1 min) and the stain free imaged, before being transferred onto a PVDF membrane using an Iblot 2 transfer apparatus (Thermo Scientific). Membranes were blocked in 5% milk in 0.1% Tween-20 in Tris-buffered saline (TBST) for 1 h at room temperature, and subsequently incubated with the indicated primary antibody in blocking buffer for 16 h at 4 °C. For detection of biotin, membranes were blocked in 1:1 PBS: Seablock, and HRP-Streptavidin was diluted in 10% seablock:TBST.

Membranes were washed (3 x 10 min, TBST), incubated with HRP-conjugated secondary antibody, washed again, developed using Supersignal West Atto Maximum Sensitivity Substrate (Thermo Fisher) and imaged on a Fusion FX geldoc (Vilber). Membranes were stripped using BlueClear SB (Serva) and re-probed as described above.

Primary antibodies used: Goat anti-mouse Gli3 (R&D systems, AF3690), 1:200; Mouse anti-Gli1 (Cell signaling, 2643S), 1:1000; Goat anti-mouse Gli2 (R&D systems, AF3635), 1:1000; Mouse anti-BRD3 (Santa Cruz, sc-81202), 1:200; Rabbit anti-BRD4 (Bethyl laboratories, A301-985A-M), 1:1000; Rabbit anti-BRD2 (Bethyl laboratories, A302-583A-T), 1:1000; Rabbit anti-karyopherin β1 (H-300)) (Santa Cruz, sc-11367), 1:500; Mouse anti-Renilla luciferase clone 1D5.2 (Millipore, MAB4410), 1:1000; Streptavidin-HRP (Biolegend, cat. 405210, 1:1000)

Band intensities were determined using Fiji Image J (National Insitute of Health) on background subtracted images and normalized to total protein loaded using an appropriate housekeeping protein or stainfree total protein. Data were further normalized to the relevant controls (100%) as specified in the Figure legends. For the competition assays shown in Figure 5 and Supplementary Figure 2, two DMSO samples were included in each experiment, and the values of these were averaged to 100%. As HPI-1 and compound **1** were included in the same experiment, the DMSO and HPP-9 values in these graphs are the same and include the additional technical replicates.

2.2 Synthesis

2.2.1 General.

Anhydrous reactions were set up under inert atmosphere (argon) utilizing glassware that was oven dried and cooled under argon purging or under N_2 using standard Schlenk line technology. Silica Gel Flash Column Chromatography was performed on Silica gel Merck 60 (particle size 40-63 μm). Starting materials were purchased directly from commercial suppliers (Sigma Aldrich, Acros, Alfa Aesar, Fluorochem) and used without further purification unless otherwise stated. All solvents were dried according to standard procedures or bought from commercial suppliers. Reactions were monitored using thin-layer chromatography (TLC) on Merck silica gel aluminium plates with F254 indicator. Visualization of the developed plates was performed under UV light (254 and 365 nm).
NMR characterization data (^{1}H NMR, ^{13}C NMR and 2D spectra) were collected at 300 K on a Bruker DRX300 (300 MHz), Bruker DRX400 (400 MHz) and Bruker DRX500 (500 MHz). Chemical shifts δ are reported in ppm downfield from tetramethylsilane using the residual deuterated solvent signals as an internal reference ($CDCl_3$: δ H = 7.26 ppm, δ C = 77.16 ppm; CD_3OD: δ H = 3.31 ppm, δ C = 49.00 ppm). For ^{1}H, ^{13}C and NMR, coupling constants *J* are given in Hz and the resonance multiplicity is described as s (singlet), d (doublet), t (triplet), q (quartet), m (multiplet) and br. (broad).

LC-MS spectra were recorded on a DIONEX Ultimate 3000 UHPLC with a Thermo LCQ Fleet Mass Spectrometer System using a PINNACLE DB C18 column (1.9 µm, 50 x 2.1 mm) operated in positive mode. All the LC-MS spectra were measured by electrospray ionization (ESI). Buffer A: 0.05% TFA in H_2O, Buffer B: 0.05% TFA in ACN. Analytical gradient was from 30% to 90% B within 4 min with 0.75 mL/min flow unless otherwise stated. HR-MS was obtained in the Chemical Biology Mass Spectrometry core facility (ChemBioMS, University of Geneva) by chip-based nanospray infusion using a TriVersa NanoMate (Advion Interchim Scientific, Harlow, UK) hyphenated to a high resolution Q Exactive Plus hybrid quadrupole-orbitrap mass spectrometer (ThermoFisher Scientific, Reinach, Switzerland). For LC-MS or HR-MS analysis, samples were prepared in water:methanol:acetonitrile 1:1:1.

2.2.2 Experimental procedures.

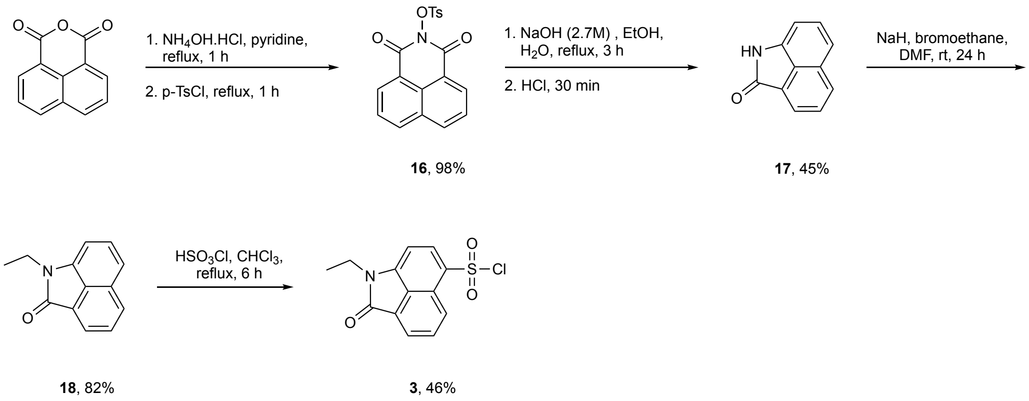

Supplementary Scheme 3. Synthesis of intermediate **3**, according to Xue et al.[6]

1,3-dioxo-1*H*-benzo[*de*]isoquinolin-2(3*H*)-yl 4-methylbenzenesulfonate (16)

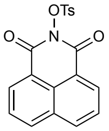

Naphthalic anhydride (991 mg, 5 mmol, 1 eq.) and hydroxylamine hydrochloride (347 mg, 5 mmol, 1 eq.) were combined as a solution in pyridine (40 mL). The reaction was conducted under reflux for 1 hour followed by cooling to 80 °C. To the reaction mixture was added powdered p-toluenesulfonyl chloride (1.90 g, 10 mmol, 2 eq.). After the addition, the reaction was performed under reflux for 1 hour. After cooling to room temperature, the reaction mixture was poured into ice water (6.25 mL) and stirred to precipitate crystals. The precipitate was filtered and rinsed with additional cool water (2 mL) and $NaHCO_3$ (aq. sat. 2 mL) to give compound **16** (1.8 g, 4.9 mmol, 98%) as a yellow solid. R_f = 0.9 (1:1, pentane:EtOAc).^{1}H NMR (300 MHz, $CDCl_3$) δ 8.63 (d, *J* = 7.3, 2H), 8.28 (d, *J* = 8.3, 2H), 8.05 (d, *J* = 8.1 Hz, 2H), 7.79 (t, *J* = 7.7 Hz, 2H), 7.43 (d, *J* =

8.1 Hz, 2H), 2.51 (s, 3H). ^{13}C NMR (75 MHz, $CDCl_3$) δ 160.04, 146.59, 135.33, 132.51, 132.35, 132.03, 129.99, 129.63, 127.67, 127.35, 122.45, 22.06. LC-MS analysis t_R: 2.03 min, ESI-MS (m/z): $[M+H]^+$: 367.94.

benzo[*cd*]indol-2(1*H*)-one (17)

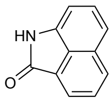

To a solution of compound **16** (753 mg, 2 mmol, 1 eq.) in EtOH (4.1 mL) and water (3.4 mL) was added an aqueous solution of NaOH (2.7 mol/L, 0.2 mL) at room temperature. The mixture was heated to reflux for 3 hours while distilling the EtOH. After the reaction was completed, the reaction mixture was cooled to 75°C, concentrated HCl was added dropwise, and a yellow precipitate was formed. Then, the mixture was further cooled. The precipitate was collected by filtration and washed with water. The resulting crude product was purified by silica gel chromatography (10% EtOAc in DCM) to yield compound **17** (153 mg, 0.9 mmol, 45%) as a yellow solid. R_f = 0.53 (1:1, pentane:EtOAc). ^{1}H NMR (400 MHz, $CDCl_3$) δ 8.09 (d, *J* = 8.5 Hz, 1H), 8.06 (d, *J* = 8.5 Hz, 1H), 8.02 (s, 1H), 7.75 (d, *J* = 8.1 Hz, 1H), 7.57 (d, *J* = 8.5 Hz, 1H), 7.47 (d, *J* = 8.5 Hz, 1H), 6.98 (d, *J* = 7.0, 1H). ^{13}C NMR (101 MHz, $CDCl_3$) δ 169.70, 137.08, 131.33, 129.63, 128.88, 128.74, 126.74, 126.60, 124.59, 120.49, 106.41. LC-MS analysis t_R: 0.66 min, ESI-MS (m/z): $[M+H]^+$: 170.00.

1-ethylbenzo[*cd*]indol-2(1*H*)-one (18)

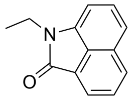

Compound **17** (178 mg, 1.05 mmol, 1 eq.) and NaH (126 mg, 5.25 mmol, 5 eq.) were dissolved in DMF (3.2 mL). Bromoethane (229.0 mg, 156 µL, 2.1 mmol, 2 eq.) was added dropwise into the solution and the reaction mixture was stirred at room temperature for 24 hours. Then, the reaction mixture was washed with water and extracted with EtOAc. The combined organic layers were washed with brine, dried over $MgSO_4$, filtered, and concentrated under reduced pressure. The crude mixture was purified by silica column chromatography (10→50% EtOAc in pentane) resulting in compound **18** (170 mg, 0.86 mmol, 82%) as a yellow solid. R_f = 0.71 (1:1, pentane:EtOAc). ^{1}H NMR (300 MHz, $CDCl_3$) δ 8.10 – 8.03 (m, 1H), 8.01 (dd, *J* = 8.2, 0.6 Hz, 1H), 7.71 (dd, *J* = 8.1, 7.0 Hz, 1H), 7.54 (dd, *J* = 8.4, 0.8 Hz, 1H), 7.47 (dd, *J* = 8.5, 6.8 Hz, 1H), 6.92 (dd, *J* = 6.8, 0.8 Hz, 1H), 3.99 (q, *J* = 7.2 Hz, 2H), 1.38 (t, *J* = 7.2 Hz, 3H). ^{13}C NMR (75 MHz, $CDCl_3$) δ 167.91, 139.35, 130.84, 129.28, 128.78, 128.63, 127.05, 125.38, 124.32, 120.30, 104.95, 35.11, 14.18. LC-MS analysis t_R: 1.45 min, ESI-MS (m/z): $[M+H]^+$: 196.03.

1-ethyl-2-oxo-1,2-dihydrobenzo[*cd*]indole-6-sulfonyl chloride (3)

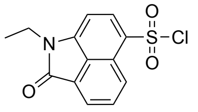

To a solution of compound **18** (45 mg, 0.23 mmol, 1 eq.) in chloroform (1.4 mL) was added to batches of chlorosulfonic acid (80 mg, 46 µL, 0.69 mmol, 3 eq.) at 0°C for 10 minutes. The reaction mixture was heated at 50°C for 6 hours. The mixture was then poured into ice water and extracted with DCM. The combined organic layers were washed with brine, dried over $MgSO_4$, filtered, and concentrated under reduced pressure. The resulting crude product was purified by silica column chromatography (20→50% EtOAc in pentane) resulting in compound **3** (32 mg, 0.11 mmol, 46%). R_f = 0.6 (4:1, pentane:EtOAc). ^1H NMR (300 MHz, $CDCl_3$) δ 8.72 (d, *J* = 8.4 Hz, 1H), 8.32 (d, *J* = 7.8 Hz, 1H), 8.16 (d, *J* = 7.1 Hz, 1H), 7.95 (dd, *J* = 8.4, 7.0 Hz, 1H), 6.96 (d, *J* = 7.8 Hz, 1H), 4.00 (q, *J* = 7.3 Hz, 2H), 1.39 (t, *J* = 7.2 Hz, 3H). ^{13}C NMR (75 MHz, $CDCl_3$) δ 167.44, 146.52, 133.81, 133.06, 131.83, 129.24, 126.63, 126.33, 126.02, 124.10, 102.64, 35.47, 14.01.LC-MS analysis t_R: 2.03 min, ESI-MS (m/z): $[M+H]^+$: 296.07.

tert-butyl (1,2-diazaspiro[2.5]oct-1-en-6-yl)carbamate (6)

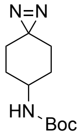

N-4-Boc-aminocyclohexanone **4** (213 mg, 1 mmol) was dissolved in liquid NH_3 (6 mL) and DCM (3 mL) in a sealed tube at -78°C. Hydroxylamine-O-sulfonic acid (124 mg, 1.1 mmol) was carefully added at the same temperature. After closing the tube, the reaction mixture was stirred at room temperature for 24 hours. The reaction mixture was dissolved in MeOH (1.2 mL) and Et_3N (0.25 mL) was added. Then I_2 was added portionwise until no decolorization took place. The reaction mixture was stirred for an additional 2 hours and then concentrated under vacuum. The residue was dissolved in EtOAc and washed with $Na_2S_2O_3$ (aq.) until the disappearance of the red color. The organic layer was dried over anhydrous $MgSO_4$ and concentrated under reduced pressure. The crude was purified by silica gel chromatography (20% EtOAc in pentane) resulting in compound **6** (111 mg, 0.49 mmol, 49%) as a white solid. R_f = 0.84 (4:1, pentane:EtOAc). ^1H NMR (400 MHz, $CDCl_3$) δ 4.53 (s, 1H), 3.58 (d, *J* = 9.3 Hz, 1H), 2.04 – 1.92 (m, 2H), 1.83 – 1.70 (m, 2H), 1.59 – 1.45 (m, 2H), 1.43 (s, 9H), 0.74 – 0.63 (m, 2H). ^{13}C NMR (101 MHz, $CDCl_3$) δ 134.99, 78.92, 55.90, 42.96, 41.94, 41.15, 40.40. LC-MS analysis t_R: 1.59 min, ESI-MS (m/z): $[M+H]^+$: 225.95.

1-ethyl-2-oxo-*N*-(1,2-diazaspiro[2.5]oct-1-en-6-yl)-1,2-dihydrobenzo[*cd*]indole-6-sulfonamide (7)

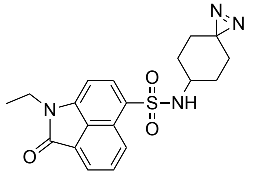

Compound **6** (45 mg, 0.2 mmol) was dissolved in a solution of 4M HCl in dioxane (0.2 mL). The reaction mixture was stirred for 1 hour and concentrated under reduced pressure. Then, it was washed with $NaHCO_3$ (aq. sat.) and extracted with EtOAc. The residue was dried under reduced pressured. To a reaction mixture of crude amine (32 mg, 0.1 mmol, 1 eq.) and compound **3** (25 mg, 0.2 mmol, 2 eq.) in DCM (4.6 mL) DIPEA (263 mg, 0.35 mL, 2 mmol, 20 eq.) was added, and the reaction mixture stirred at room temperature for 3 hours. Then, the reaction mixture was washed with water, extracted with DCM, and the combined organic layers were washed with water and brine, dried over $MgSO_4$, filtered, and concentrated under reduced pressure. The residue was purified by silica gel chromatography (20→60% EtOAc in pentane) resulting in compound **7** (28 mg, 73 μmol, 68%) as a yellow oil. R_f = 0.68 (2:1, pentane:EtOAc). ^{1}H NMR (300 MHz, $CDCl_3$) δ 8.63 (dd, *J* = 8.4, 0.6 Hz, 1H), 8.23 (d, *J* = 7.6 Hz, 1H), 8.12 (d, *J* = 6.9 Hz, 1H), 7.85 (dd, *J* = 8.4, 7.0 Hz, 1H), 6.93 (d, *J* = 7.6 Hz, 1H), 4.81 (d, *J* = 7.6 Hz, 1H), 3.99 (q, *J* = 7.2 Hz, 2H), 3.41 – 3.29 (m, 1H), 1.88 – 1.73 (m, 2H), 1.61 – 1.47 (m, 4H), 1.39 (t, *J* = 7.2 Hz, 3H), 0.80 – 0.63 (m, 2H). ^{13}C NMR (75 MHz, $CDCl_3$) δ 167.75, 144.20, 132.92, 130.83, 129.29, 127.03, 126.17, 125.58, 124.79, 103.04, 51.43, 35.33, 31.52, 29.14, 27.05, 14.05. LC-MS analysis t_R: 1.78 min, ESI-MS (m/z): $[M+H]^+$: 384.92.

1-ethyl-2-oxo-*N*-(prop-2-yn-1-yl)-*N*-(1,2-diazaspiro[2.5]oct-1-en-6-yl)-1,2-dihydrobenzo [*cd*]indole-6-sulfonamide (PAL-1)

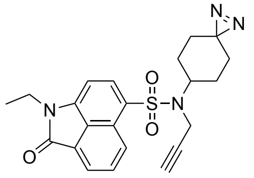

Compound **7** (30 mg, 0.07 mmol) and NaH (10 mg, 0.41 mmol, 6 eq.) were dissolved in DMF (0.22 mL). Propargyl bromide (26 mg, 17 μL, 0.18 mmol, 2.5 eq.) was added dropwise into the solution and the reaction mixture was stirred at room temperature for 48 hours. Then, the reaction mixture was washed with water and extracted with EtOAc. The combined organic layers were washed with brine, dried over $MgSO_4$, filtered, and concentrated under reduced pressure. The crude mixture was purified by silica column chromatography (10% EtOAc in pentane) resulting in compound **II-3** (11 mg, 26 μmol, 36%). R_f = 0.62 (2:1, pentane:EtOAc). ^{1}H NMR (300 MHz, $CDCl_3$) δ 8.67 (d, *J* = 8.5 Hz, 1H), 8.26 (d, *J* = 7.6 Hz, 1H), 8.10 (d, *J* = 7.0 Hz, 1H), 7.84 (dd, *J* = 8.5, 7.0 Hz, 1H), 6.93 (d, *J* = 7.6 Hz, 1H), 4.21 (d, *J* = 2.5 Hz, 2H), 4.00 (p, *J* = 6.9 Hz, 3H), 2.06 (q, *J* = 3.5, 3.0 Hz, 1H), 2.01 – 1.91 (m, 3H), 1.79 – 1.67 (m, 2H), 1.39 (t, *J* = 7.2 Hz, 3H), 1.25 (s, 1H), 0.61 – 0.46 (m, 2H). ^{13}C NMR (75 MHz, $CDCl_3$) δ 167.75, 144.19, 133.67,

130.51, 129.90, 129.09, 126.85, 126.06, 125.51, 125.09, 103.08, 80.12, 72.84, 56.64, 35.31, 32.14, 30.62, 29.13, 27.07, 14.04. LC-MS analysis t_R: 2.09 min, ESI-MS (m/z): $[M+H]^+$: 422.96. HR-MS (ESI): m/z calc. for $C_{22}H_{22}N_4O_3S$ $[M+H]^+$ 423.1485, found 423.1489.

ethyl 3-oxohept-6-ynoate (8a)

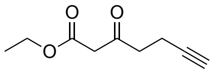

Ethyl acetoacetate (997 mg, 0.98 mL, 7.66 mmol, 1 eq.) was slowly added in a heterogeneous mixture of NaH/mineral oil (643 mg, 16.1 mmol, 2.1 eq.) in dry THF (10.2 mL) at 0°C. After 30 minutes 1.45 M n-BuLi in hexane (764 mg, 5.75 mL, 11.9 mmol, 1.2 eq.) was added dropwise at -78°C and the reaction mixture stirred for 1 hour. Propargyl bromide (1.48 g, 1.1 mL, 9.9 mmol, 1.3 mmol) was then added portion wise and the reaction mixture stirred for 2 hours. Then the reaction was quenched with water. The mixture was extracted with EtOAc, washed with 1M HCl and brine, and the combined organic phases were dried over $MgSO_4$. The solvent was removed under reduced pressure and the crude product was purified by silica column chromatography (20→50% EtOAc in pentane) resulting in compound **8a** (1.15 g, 6.85 mmol) in 89% yield. R_f = 0.71 (3:1, pentane:EtOAc). ^1H NMR (300 MHz, $CDCl_3$) δ 4.20 (q, *J* = 7.1 Hz, 2H), 3.46 (s, 2H), 2.81 (dd, *J* = 7.8, 6.6 Hz, 2H), 2.53 – 2.37 (m, 2H), 1.96 (t, *J* = 2.7 Hz, 1H), 1.28 (t, *J* = 7.1 Hz, 3H). ^{13}C NMR (75 MHz, $CDCl_3$) δ 200.55, 166.91, 82.52, 68.99, 61.52, 49.23, 41.62, 14.10, 12.84.

ethyl 2-(2-(but-3-yn-1-yl)-1,3-dioxolan-2-yl)acetate (10a)

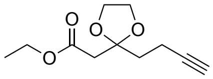

Ethyleneglycol (307 mg, 0.28 mL, 4.95 mmol, 1.5 eq.) and p-toluenesulfonic acid (13 mg, 0.07 mmol, 0.02 eq.) were added to a solution of compound **8a** (555 mg, 3.3 mmol, 1 eq.) in benzene (2.6 mL) and the mixture was heated with a Dean-Stark apparatus for 5 hours. After cooling to room temperature, the solvent was evaporated and the residue was dissolved in EtOAc. The organic phase was washed with 5% $NaHCO_3$ and brine, dried over $MgSO_4$ and the solvent was evaporated under reduced pressure. The product **10a** (501 mg, 2.36 mmol) was collected as a colorless oil in 71% yield, and it was used for the next step without further purification. R_f = 0.68 (3:1, pentane:EtOAc). ^1H NMR (300 MHz, $CDCl_3$) δ 4.04 (q, *J* = 7.2 Hz, 2H), 3.94 – 3.80 (m, 4H), 2.54 (s, 2H), 2.25 – 2.13 (m, 2H), 2.06 – 1.90 (m, 2H), 1.85 (t, *J* = 2.6 Hz, 1H), 1.16 (t, *J* = 7.2 Hz, 3H). ^{13}C NMR (75 MHz, $CDCl_3$) δ 169.00, 108.15, 83.78, 68.10, 65.07, 60.43, 42.54, 36.29, 14.04, 12.65.

2-(2-(but-3-yn-1-yl)-1,3-dioxolan-2-yl)ethan-1-ol (11a)

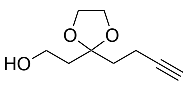

$LiAlH_4$ (135 mg, 3.56 mmol, 1.5 eq.) was added slowly to a solution of compound **10a** (501 mg, 2.36 mmol, 1 eq.) in dry Et_2O (10.3 mL) at 0°C. The reaction was refluxed for 90 minutes and subsequently stirred for 24 hours at room temperature. After cooling to 0°C, water (2.1 mL) and 1N KOH (2.1 mL) were added slowly to the reaction mixture. The solution was filtered over silica gel and rinsed with Et_2O. The organic layer was separated and dried over $MgSO_4$. The solvent was evaporated under reduced pressure resulting in compound **11a** (317 mg, 1.86 mmol) as a yellow oil in 79% yield and was used for the next step without further purification. R_f = 0.60 (3:1, pentane:EtOAc). ^{1}H NMR (400 MHz, $CDCl_3$) δ 4.06 – 3.93 (m, 4H), 3.76 (q, *J* = 5.6 Hz, 2H), 2.61 (t, *J* = 5.7 Hz, 1H), 2.32 – 2.23 (m, 2H), 1.96 – 1.91 (m, 5H). ^{13}C NMR (101 MHz, $CDCl_3$) δ 111.24, 84.08, 68.38, 65.09, 58.87, 38.41, 36.07, 13.29.

1-hydroxyhept-6-yn-3-one (12a)

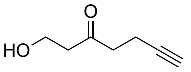

Compound **11a** (289 mg, 1.7 mmol, 1 eq.) was treated with p-TsOH (85 mg, 0.49 mmol, 0.3 eq.) in acetone (5.2 mL). The mixture was stirred at room temperature for 3 hours. Then, the reaction mixture was washed with water and extracted with Et_2O. The combined organic layers were washed with $NaHCO_3$ (aq. sat.) and brine, dried over $MgSO_4$, filtered, and concentrated under reduced pressure. The product **12a** was collected (140 mg, 1.1 mmol) as a light orange oil in 65% yield, and it was used for the next step without further purification. R_f = 0.2 (3:1, pentane:EtOAc). ^{1}H NMR (300 MHz, $CDCl_3$) δ 3.87 (q, *J* = 4.6 Hz, 2H), 2.76 – 2.65 (m, 4H), 2.53 – 2.41 (m, 2H), 2.34 (d, *J* = 6.4 Hz, 1H), 1.96 (t, *J* = 2.7 Hz, 1H). ^{13}C NMR (75 MHz, $CDCl_3$) δ 209.23, 82.90, 69.05, 57.89, 44.69, 41.94, 12.95.

2-(3-(but-3-yn-1-yl)-3*H*-diazirin-3-yl)ethan-1-ol (13a)

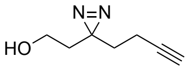

Method A:

Compound **12a** (219 mg, 1.74 mmol, 1 eq.) and hydroxylamine-O-sulfonic acid (217 mg, 1.91 mmol, 1.1 eq.) were dissolved in liquid ammonia (7 mL) and dry DCM (2 mL) in a sealed tube at -78°C. The reaction mixture was stirred at room temperature for 24 hours. After evaporation of the solvent under reduced pressure, the reaction mixture was re-dissolved in dry MeOH (2 mL) and Et_3N (264 mg, 0.36 mL, 2.61 mmol, 1.5 eq.) was added. Then iodine was added portionwise, until no decolorization took place and the reaction mixture was stirred for an additional 2 hours at room temperature. The residue was dissolved in EtOAc and washed with $Na_2S_2O_3$ (aq. sat.) until

disappearance of the red color. The organic layer was dried over $MgSO_4$, filtered and concentrated under reduced pressure. Crude was purified by silica column chromatography (50% EtOAc in pentane) resulting in compound **13a** (187 mg, 1.35 mmol) as a light orange oil, in 78% yield.

Method B:

Compound **12a** (260 mg, 2 mmol, 1 eq.) was added to a solution of 7N NH_3 (5.2 mL) in methanol. Then *t*-butyl hypochlorite solution (5.2 mL, 10.3 mmol, 6 eq.) was added to the reaction mixture and left to stir overnight at room temperature. After degassing for 20 minutes, another t-butyl hypochlorite batch (1.6 mL, 0.35 mmol, 1.5 eq.) was added and the reaction mixture stirred for 1 hour at room temperature. The solvent was then removed under reduced pressure and the residue was redissolved in sodium thiosulfate (sat.) and extracted with Et_2O. The organic layers were combined, dried over $MgSO_4$, filtered and concentrated under reduced pressure. The crude was purified by silica column chromatography (3→1 EtOAc in pentane) resulting in compound **13a** (145 mg, 1.05 mmol) in 51% yield. R_f = 0.73 (4:1, pentane:EtOAc). ^1H NMR (300 MHz, $CDCl_3$) δ 3.49 (t, *J* = 6.2 Hz, 2H), 2.10 – 1.97 (m, 3H), 1.76 – 1.64 (m, 4H), 1.58 (s, 1H). ^{13}C NMR (75 MHz, $CDCl_3$) δ 82.98, 69.36, 57.52, 35.65, 32.78, 26.73, 13.37.

2-(2-(3-(but-3-yn-1-yl)-3*H*-diazirin-3-yl)ethyl)isoindoline-1,3-dione (14a)

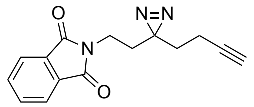

In a solution of compound **13a** (55 mg, 0.4 mmol, 1 eq.) in dry toluene (4 mL) phthalimide (117 mg, 0.8 mmol, 2 eq.) was added. Triphenylphosphine (209 mg, 0.8 mmol, 2 eq.) was added and the reaction mixture was stirred at room temperature before it was cooled down to 0°C and DEAD (365 µL, 0.80 mmol, 2 eq.) was added dropwise. The reaction was stirred for 1 hour at room temperature. The solvent was then removed under reduced pressure and the reaction mixture was re-dissolved in EtOAc. Saturated Na_2CO_3 (sat.) was added and the aqueous phase was extracted with additional EtOAc. The combined organic phases were then washed with brine, dried with $MgSO_4$ and concentrated under vacuum. The crude was purified by flash column chromatography (20:1→7:1 pentane:EtOAc) resulting in compound **14a** (83 mg, 0.31 mmol) in 78% yield. R_f = 0.65 (3:1 pentane:EtOAc). ^1H NMR (300 MHz, $CDCl_3$) δ 7.86 (dd, *J* = 5.4, 3.1 Hz, 2H), 7.73 (dd, *J* = 5.5, 3.0 Hz, 2H), 3.69 (t, *J* = 7.3 Hz, 2H), 2.06 – 1.97 (m, 3H), 1.83 – 1.70 (m, 4H). ^{13}C NMR (75 MHz, $CDCl_3$) δ 168.22, 134.22, 132.19, 123.51, 82.68, 69.50, 33.25, 32.04, 31.63, 26.64, 13.41.

2-(3-(but-3-yn-1-yl)-3*H*-diazirin-3-yl)ethan-1-amine (15a)

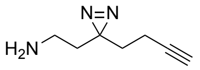

Hydrazine monohydrate (0.1 mL, 1.39 mmol, 3 eq.) was added to solution of compound **14a** (124 mg, 0.46 mmol, 1 eq.) in 5 mL of MeOH and was stirred at room temperature for 30 minutes. Then, 2 mL of 5% of aqueous HCl (aq.) was added to the reaction mixture and was left to stir. The pH was then adjusted to 2 with 1 M HCl and a white precipitate appeared. After filtration, the aqueous phase was extracted with Et_2O. The aqueous phase was then alkalized to pH=10 with solid KOH before it was extracted with Et_2O. All organic phases were combined, washed with brine, dried with Na_2SO_4 and concentrated under vacuum. The crude was purified by flash column chromatography (36:1→9:1 DCM:MeOH) resulting in compound **15a** (36 mg, 0.26 mmol) in 57% yield. R_f = 0.1 (DCM:MeOH 9:1). ^1H NMR (300 MHz, $CDCl_3$) δ 2.51 (t, *J* = 6.9 Hz, 2H), 2.09 – 1.92 (m, 3H), 1.64 (q, *J* = 7.2 Hz, 4H). ^{13}C NMR (75 MHz, $CDCl_3$) δ 82.78, 69.53, 36.26, 34.71, 32.45, 26.38, 13.41.

***N*-(2-(3-(but-3-yn-1-yl)-3*H*-diazirin-3-yl)ethyl)-1-ethyl-2-oxo-1,2-dihydrobenzo[*cd*]indole-6-sulfonamide (PAL-2)**

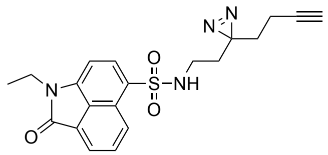

To a solution of compound **3** (41 mg, 0.14 mmol, 1 eq.) and **15a** (36 mg, 0.26 mmol, 1.9 eq.) in DCM (1.1 mL) DIPEA (120 µL, 0.7 mmol, 5 eq.) was added and stirred for 3 hours at room temperature. Then, the reaction mixture was washed with water and extracted with DCM. The combined organic layers were washed with water and brine, dried with $MgSO_4$, and concentrated under vacuum. The crude was purified by flash column chromatography (10:1→4:1 pentane:EtOAc) resulting in **PAL-2** (73 mg, 0.18 mmol) in 97% yield. R_f = 0.25 (3:1 pentane:EtOAc). ^1H NMR (400 MHz, CD_3OD) δ 8.72 (d, *J* = 8.4, 0.6 Hz, 1H), 8.18 (d, *J* = 7.6 Hz, 1H), 8.12 (d, *J* = 7.1, 0.6 Hz, 1H), 7.97 – 7.86 (m, 1H), 7.21 (d, *J* = 7.6 Hz, 1H), 4.01 (q, *J* = 7.2 Hz, 2H), 2.75 (t, *J* = 7.2 Hz, 2H), 2.17 (t, *J* = 2.7 Hz, 1H), 1.81 (td, *J* = 7.5, 2.6 Hz, 2H), 1.51 – 1.31 (m, 7H). ^{13}C NMR (101 MHz, CD_3OD) δ 169.48, 144.74, 133.86, 131.57, 131.22, 130.75, 127.80, 127.07, 126.36, 126.04, 105.11, 83.44, 70.27, 38.62, 36.15, 34.11, 33.15, 27.46, 14.10, 13.70. LC-MS analysis t_R: 1.80 min, ESI-MS (m/z): $[M+H]^+$: 396.92. HR-MS (ESI): m/z calc. for $C_{20}H_{20}N_4O_3S$ $[M+H]^+$ 397.1329, found 397.1299

3-oxocyclohex-1-en-1-yl trifluoromethanesulfonate (9)

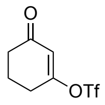

A nitrogen-purged flask was charged with cyclohexane-1,3-dione (1.66 g, 14.8 mmol, 1 eq.) in dry DCM (88.5 mL). Pyridine (2.38 mL, 29.5 mmol, 2 eq.) was added and the reaction mixture was cooled to -78°C. Trifluoromethanesulfonic anhydride (2.98 mL, 17.7 mmol, 1.2 eq.) was added slowly and the reaction mixture was stirred for 10 minutes at -78°C before it was warmed to 0°C and left to stir for 1 hour. The crude was quenched with 1 M HCl (29.5 mL) and was extracted with Et_2O. The organic layers were combined, washed with Na_2CO_3, water, dried over $MgSO_4$ and concentrated under vacuum. The crude was purified by flash column chromatography (15:1→10:1→8:1 pentane:EtOAc) resulting in compound **9** (3.24 g, 13.3 mmol) in 90% yield. R_f = 0.5 (6:1 pentane:EtOAc). ^{1}H NMR (300 MHz, $CDCl_3$) δ 6.06 (d, *J* = 1.3 Hz, 1H), 2.69 (td, *J* = 6.2, 1.4 Hz, 2H), 2.45 (dd, *J* = 7.6, 5.9 Hz, 2H), 2.18 – 2.08 (m, 2H). ^{13}C NMR (75 MHz, $CDCl_3$) δ 197.48, 167.49, 119.41, 36.48, 28.63, 20.92.

ethyl 3-oxooct-7-ynoate (8b)

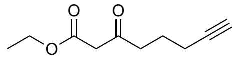

To a solution of dry EtOAc (0.39 mL, 4 mmol, 1.2 eq.) in dry THF (6.7 mL) 1M LiHMDS (3.69 mL, 3.7 mmol, 1.1 eq.) was added at -78°C under inert atmosphere and stirred for 30 minutes. Then **9** (410 mg, 1.68 mmol, 0.5 eq.) was added and the reaction mixture left at -78°C for 10 minutes before it warmed up to 0°C for 10 minutes, at room temperature for 30 minutes, and at 60°C for 30 minutes. Saturated aqueous NH_4Cl solution was added to quench the reaction. The mixture was extracted with Et_2O and the organic phases combined, washed with water, brine, dried over $MgSO_4$, filtered, and concentrated under reduced pressure. The crude was purified by flash column chromatography (20:1→15:1 pentane:EtOAc) resulting in compound **8b** (56 mg, 0.31 mmol) in 18% yield. ^{1}H NMR (300 MHz, $CDCl_3$) δ 4.26 – 4.14 (m, 2H), 3.44 (d, *J* = 2.2 Hz, 2H), 2.70 (t, *J* = 7.1 Hz, 2H), 2.29 – 2.18 (m, 2H), 1.96 (t, *J* = 2.6 Hz, 1H), 1.82 (p, *J* = 7.0 Hz, 2H), 1.28 (t, *J* = 7.1 Hz, 3H). ^{13}C NMR (75 MHz, $CDCl_3$) δ 202.81, 168.42, 80.17, 69.35, 61.56, 49.58, 41.49, 22.12, 17.73, 14.24.

ethyl 2-(2-(pent-4-yn-1-yl)-1,3-dioxolan-2-yl)acetate (10b)

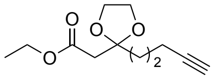

Ethyleneglycol (0.35 mL, 6.3 mmol, 1.5 eq.) and p-toluenesulfonic acid (14 mg, 0.07 mmol, 0.02 eq.) were added to a solution of compound **8b** (673 mg, 3.4 mmol, 1 eq.) in benzene (3 mL) and the mixture was heated with a Dean-Stark apparatus for 5 hours. After cooling to room temperature, the solvent was evaporated, and the residue was dissolved in EtOAc. The organic phase was washed with 5% $NaHCO_3$ and brine, dried over $MgSO_4$ and the solvent was evaporated under reduced pressure. The product **10b** (254 mg, 1.1 mmol) was collected in 44% yield, and was used for the next step without further purification. R_f = 0.38 (DCM/MeOH 1%). ^{1}H NMR (300 MHz, $CDCl_3$) δ 4.15 (q, *J* = 7.1 Hz, 2H), 4.05 – 3.93 (m, 4H), 2.64 (s, 2H), 2.22 (td, *J* = 7.2, 2.6 Hz, 2H), 1.98 – 1.91 (m, 3H), 1.71 – 1.60 (m, 2H), 1.27 (t, *J* = 7.1 Hz, 3H). ^{13}C NMR (75 MHz, $CDCl_3$) δ 169.58, 109.26, 84.29, 68.65, 65.29, 60.72, 42.93, 36.81, 22.70, 18.58, 14.33.

1-hydroxyoct-7-yn-3-one (12b)

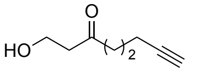

$LiAlH_4$ (72 mg, 1.9 mmol, 1.5 eq.) was added slowly to a solution of compound **10b** (283 mg, 1.2 mmol, 1 eq.) in dry Et_2O (5 mL) at 0°C. The reaction was refluxed for 90 minutes and stirred for 24 hours at room temperature. After cooling to 0°C, water (1 mL) and 1N KOH (1 mL) were added slowly to the reaction mixture. The solution was filtered over silica gel and rinsed with Et_2O. The organic layer was separated and dried over $MgSO_4$. The solvent was evaporated under reduced pressure resulting in compound **11b**, which was used for the next step without further purification. Compound **11b** (153 mg, 0.83 mmol, 1 eq.) was treated with p-TsOH (43 mg, 0.25 mmol, 0.3 eq.) in acetone (2.5 mL). The mixture was stirred at room temperature for 3 hours. Then, the reaction mixture was washed with water and was extracted with Et_2O. The combined organic layers were washed with $NaHCO_3$ (aq. sat.) and brine, dried over $MgSO_4$, filtered, and concentrated under reduced pressure. The product **12b** (105 mg, 0.75 mmol) was collected as a yellow oil in 60% yield over two steps and was used for the next step without further purification. R_f = 0.26 (DCM/MeOH 3%).^{1}H NMR (300 MHz, $CDCl_3$) δ 3.86 (t, *J* = 5.4 Hz, 2H), 2.69 (t, *J* = 5.4 Hz, 2H), 2.61 (t, *J* = 7.2 Hz, 2H), 2.24 (td, *J* = 6.8, 2.7 Hz, 2H), 1.97 (t, *J* = 2.7 Hz, 2H), 1.81 (p, *J* = 7.0 Hz, 2H). ^{13}C NMR (75 MHz, $CDCl_3$) δ 211.06, 83.53, 69.36, 58.02, 44.67, 41.78, 22.14, 17.86.

2-(3-(pent-4-yn-1-yl)-3*H*-diazirin-3-yl)ethan-1-ol (13b)

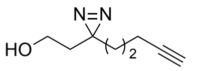

<u>Method B:</u>

Compound **12b** (94 mg, 0.7 mmol, 1 eq.) was added to a solution of 7N NH_3 (1.2 mL) in methanol. Then *t*-butyl hypochlorite solution (2.1 mL, 4 mmol, 6 eq.) was added to the reaction mixture and left to stir overnight at room temperature. After degassing for 20 minutes, another t-butyl hypochlorite batch (0.5 mL, 1 mmol, 1.5 eq.) was added and stirred for 30 minutes at room temperature. The reaction mixture was left to stir for an additional 30 minutes at room temperature. The solvent was then removed under reduced pressure and the residue was redissolved in $Na_2S_2O_3$ (aq. sat.) and extracted with Et_2O. The organic layers were combined, dried over $MgSO_4$, filtered and concentrated under reduced pressure. Crude was purified by silica column chromatography (10% EtOAc in pentane) resulting in compound **13b** (44 mg, 0.29 mmol) in 44% yield. R_f = 0.26 (4:1 pentane:EtOAc). ^{1}H NMR (400 MHz, $CDCl_3$) δ 3.48 (t, *J* = 6.2 Hz, 2H), 2.20 – 2.14 (m, 3H), 1.95 (t, *J* = 2.6 Hz, 1H), 1.68 (t, *J* = 6.2 Hz, 2H), 1.60 – 1.54 (m, 2H), 1.39 – 1.31 (m, 2H). ^{13}C NMR (101 MHz, $CDCl_3$) δ 83.49, 69.14, 57.63, 35.72, 32.22, 26.91, 22.80, 18.02.

2-(2-(3-(pent-4-yn-1-yl)-3*H*-diazirin-3-yl)ethyl)isoindoline-1,3-dione (14b)

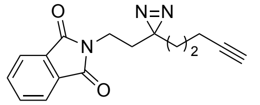

To a solution of compound **13b** (38 mg, 0.25 mmol, 1 eq.) in dry toluene (2.5 mL), phthalimide (73 mg, 0.5 mmol, 2 eq.) was added. Triphenylphosphine (164 mg, 0.62 mmol, 2 eq.) was added and reaction mixture was stirred at room temperature before it was cooled down to 0°C and DEAD (365 µL, 0.80 mmol, 2 eq.) was added dropwise. The reaction was stirred for 4 hours at room temperature. The solvent was then removed under reduced pressure and the reaction mixture was re-dissolved in EtOAc. Saturated Na_2CO_3 (sat.) was added and the aqueous phase was extracted with additional EtOAc. The combined organic phases were then washed with brine, dried with $MgSO_4$ and concentrated under vacuum. The crude was purified by flash column chromatography (20:1→7:1 pentane:EtOAc) resulting in compound **14b** (42 mg, 0.15 mmol) in 63% yield. R_f = 0.74 (3:1 pentane:EtOAc). ^{1}H NMR (400 MHz, $CDCl_3$) δ 7.85 (dd, *J* = 5.5, 3.1 Hz, 2H), 7.72 (dd, *J* = 5.5, 3.0 Hz, 2H), 3.70 – 3.63 (m, 2H), 2.16 (td, *J* = 6.9, 2.7 Hz, 2H), 1.93 (t, *J* = 2.7 Hz, 1H), 1.75 – 1.70 (m, 2H), 1.65 – 1.59 (m, 2H), 1.37 – 1.28 (m, 2H). ^{13}C NMR (101 MHz, $CDCl_3$) δ 167.58, 134.20, 132.19, 123.49, 83.35, 69.22, 33.32, 32.16, 31.08, 26.82, 22.80, 18.02.

2-(3-(pent-4-yn-1-yl)-3*H*-diazirin-3-yl)ethan-1-amine (15b)

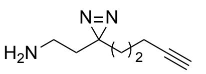

Hydrazine monohydrate (30 µL, 0.45 mmol, 3 eq.) was added to a solution of compound **14b** (42 mg, 0.15 mmol, 1 eq.) in 1.6 mL of MeOH and was stirred at room temperature for 30 minutes. Then, 0.7 mL of 5% aqueous HCl was added to the reaction mixture and was left to stir overnight. The pH was then adjusted to 2 with 1 M HCl and a white precipitate appeared. After filtration, the aqueous phase was extracted with Et_2O. The aqueous phase was then alkalized to a pH 10 with solid KOH before it was extracted with Et_2O. All organic phases were combined, washed with brine, dried with Na_2SO_4 and concentrated under vacuum. The crude was purified by flash column chromatography (36:1→9:1 DCM:MeOH) resulting in compound **15b** (7 mg, 0.05 mmol) in 31% yield. R_f = 0.1 (DCM/MeOH 10%). ^{1}H NMR (400 MHz, $CDCl_3$) δ 2.56 (t, *J* = 6.9 Hz, 2H), 2.32 (s, 2H), 2.19 – 2.14 (m, 2H), 1.95 (t, *J* = 2.6 Hz, 1H), 1.62 (t, *J* = 6.9 Hz, 2H), 1.57 – 1.51 (m, 2H), 1.38 – 1.30 (m, 2H). ^{13}C NMR (101 MHz, $CDCl_3$) δ 83.43, 69.19, 36.64, 35.76, 32.04, 27.16, 22.82, 18.03.

1-ethyl-2-oxo-*N*-(2-(3-(pent-4-yn-1-yl)-3*H*-diazirin-3-yl)ethyl)-1,2-dihydrobenzo[*cd*]indole-6-sulfonamide (PAL-3)

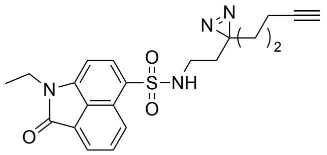

To a solution of compound **3** (7 mg, 0.024 mmol, 1 eq.) and **15b** (7 mg, 0.05 mmol, 1.9 eq.) in DCM (1 mL) DIPEA (21 µL, 0.12 mmol, 5 eq.) was added and the resulting mixture was stirred for 3 hours at room temperature. Then, the reaction mixture was washed with water and extracted with DCM. The combined organic layers were washed with water and brine, dried with $MgSO_4$, and concentrated under vacuum. Crude was purified by flash column chromatography (10:1→4:1 pentane:EtOAc) resulting in **PAL-3** (1.2 mg, 3 µmol) in 13% yield. R_f = 0.27 (2:1 pentane:EtOAc). ^{1}H NMR (400 MHz, CD_3OD) δ 8.74 (d, *J* = 8.4 Hz, 1H), 8.19 (d, *J* = 7.6 Hz, 1H), 8.14 (d, *J* = 7.0 Hz, 1H), 7.93 (dd, *J* = 8.4, 7.0 Hz, 1H), 7.22 (d, *J* = 7.6 Hz, 1H), 4.02 (q, *J* = 7.2 Hz, 2H), 2.76 (t, *J* = 7.2 Hz, 2H), 2.17 (t, *J* = 2.7 Hz, 1H), 2.00 (td, *J* = 7.0, 2.7 Hz, 2H), 1.40 (dt, *J* = 15.6, 7.2 Hz, 6H), 1.34 – 1.28 (m, 2H), 1.14 – 1.06 (m, 2H). ^{13}C NMR (101 MHz, CD_3OD) δ 169.47, 144.73, 133.85, 131.57, 131.26, 130.80, 127.79, 127.07, 126.36, 126.06, 105.11, 83.96, 70.09, 38.68, 36.15, 34.19, 32.36, 27.57, 23.84, 18.41, 14.10. LC-MS analysis t_R: 2.03 min, ESI-MS (m/z): $[M+H]^+$: 410.90. HR-MS (ESI): m/z calc. for $C_{21}H_{22}N_4O_3S$ $[M+Na]^+$ 433.1305, found 433.1317.

3. References

[1] M. E. Hom, A. E. Ondrus, T. Sakata-Kato, P. G. Rack, J. K. Chen, *ChemMedChem* **2020**, *15*, 1044–1049.

[2] J. Taipale, J. K. Chen, M. K. Cooper, B. Wang, R. K. Mann, L. Milenkovic, M. P. Scott, P. A. Beachy, *Nature* **2000**, *406*, 1005–1009.

[3] P. G. Rack, J. Ni, A. Y. Payumo, V. Nguyen, J. A. Crapster, V. Hovestadt, M. Kool, D. T. W. Jones, J. K. Mich, A. J. Firestone, S. M. Pfister, Y.-J. Cho, J. K. Chen, *Proc Natl Acad Sci U A* **2014**, *111*, 11061–11066.

[4] F. Bassilana, A. Carlson, J. A. DaSilva, B. Grosshans, S. Vidal, V. Beck, B. Wilmeringwetter, L. A. Llamas, T. B. Showalter, P. Rigollier, A. Bourret, A. Ramamurthy, X. Wu, F. Harbinski, S. Plonsky, L. Lee, H. Ruffner, P. Grandi, M. Schirle, J. Jenkins, A. W. Sailer, T. Bouwmeester, J. A. Porter, V. Myer, P. M. Finan, J. A. Tallarico, J. F. Kelleher Iii, K. Seuwen, R. K. Jain, S. J. Luchansky, *Nat. Chem. Biol.* **2014**, *10*, 343.

[5] X. Zhao, T. Ponomaryov, K. J. Ornell, P. Zhou, S. K. Dabral, E. Pak, W. Li, S. X. Atwood, R. J. Whitson, A. L. S. Chang, J. Li, A. E. Oro, J. A. Chan, J. F. Kelleher, R. A. Segal, *Cancer Res.* **2015**, *75*, 3623–3635.

[6] X. Xue, Y. Zhang, Z. Liu, M. Song, Y. Xing, Q. Xiang, Z. Wang, Z. Tu, Y. Zhou, K. Ding, Y. Xu, *J. Med. Chem.* **2016**, *59*, 1565–1579.

[7] G. Madhavi Sastry, M. Adzhigirey, T. Day, R. Annabhimoju, W. Sherman, *J. Comput. Aided Mol. Des.* **2013**, *27*, 221–234.

[8] C. Lu, C. Wu, D. Ghoreishi, W. Chen, L. Wang, W. Damm, G. A. Ross, M. K. Dahlgren, E. Russell, C. D. Von Bargen, R. Abel, R. A. Friesner, E. D. Harder, *J. Chem. Theory Comput.* **2021**, *17*, 4291–4300.

[9] R. A. Friesner, J. L. Banks, R. B. Murphy, T. A. Halgren, J. J. Klicic, D. T. Mainz, M. P. Repasky, E. H. Knoll, M. Shelley, J. K. Perry, D. E. Shaw, P. Francis, P. S. Shenkin, *J. Med. Chem.* **2004**, *47*, 1739–1749.

[10] D. K. Breslow, S. Hoogendoorn, A. R. Kopp, D. W. Morgens, B. K. Vu, M. C. Kennedy, K. Han, A. Li, G. T. Hess, M. C. Bassik, J. K. Chen, M. V. Nachury, *Nat. Genet.* **2018**, *50*, 460–471.

4. NMR spectra

^1H NMR (300 MHz) ^{13}C NMR (75 MHz) spectra of **16** ($CDCl_3$)

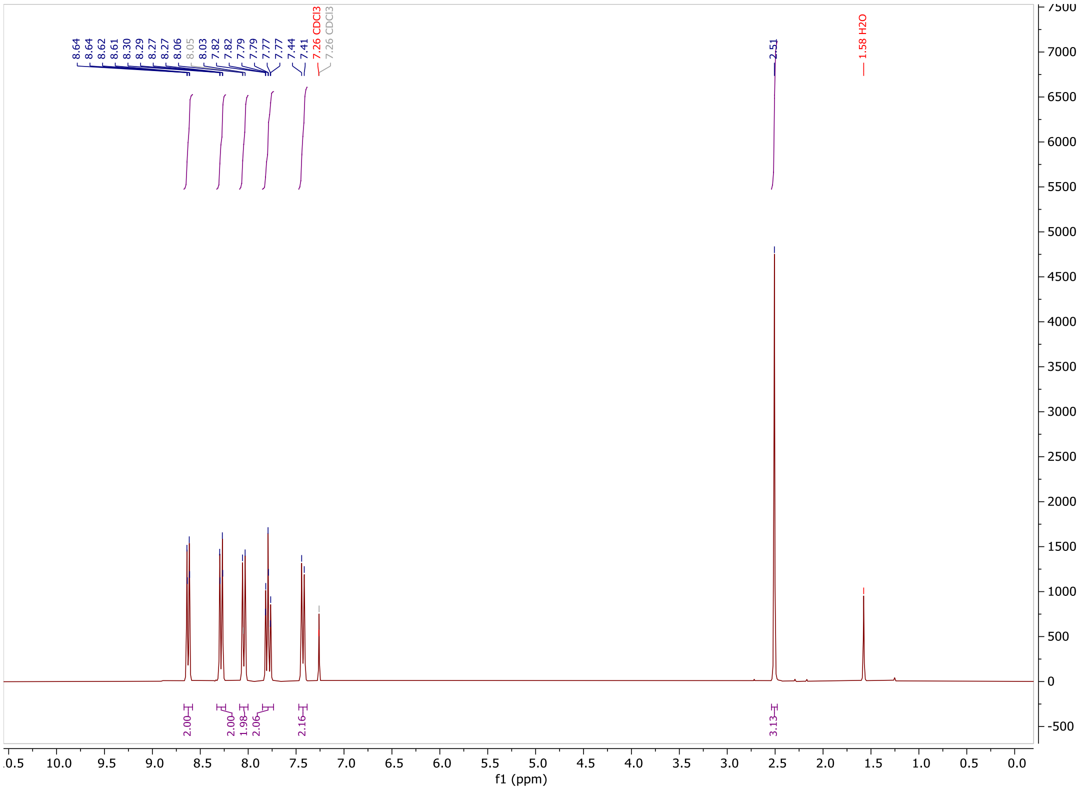

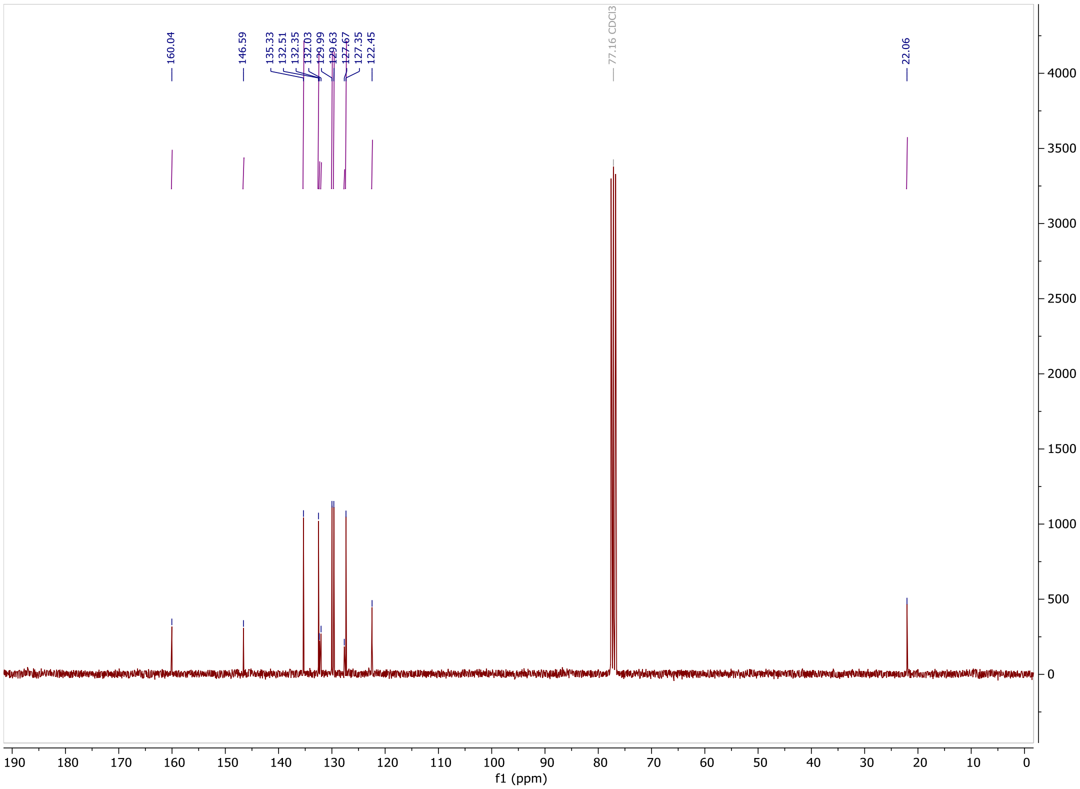

^{1}H NMR (400 MHz) ^{13}C NMR (101 MHz) spectra of **17** ($CDCl_3$)

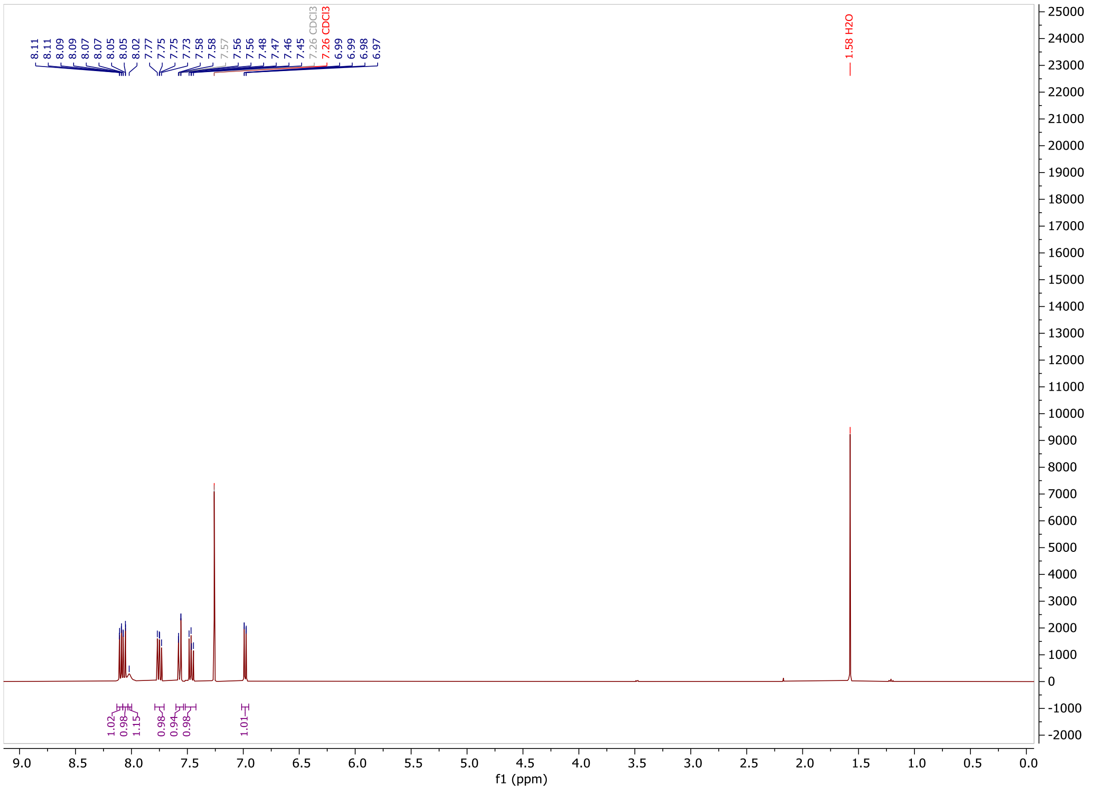

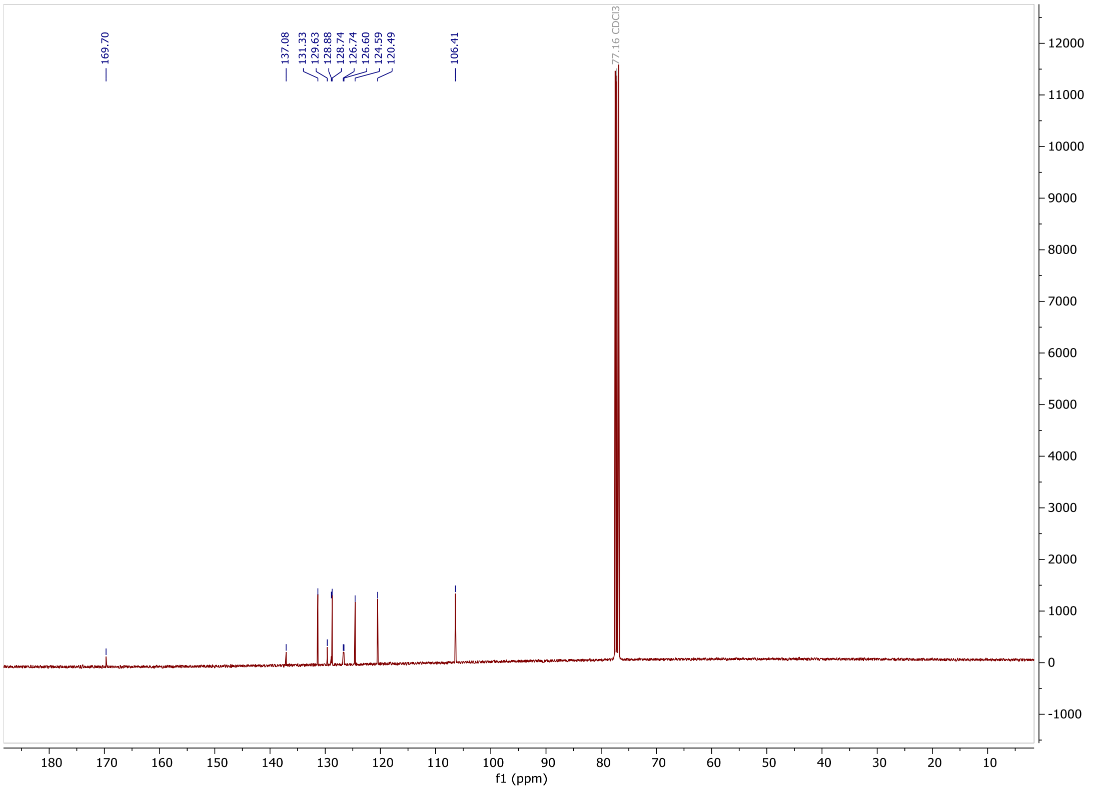

^{1}H NMR (300 MHz) ^{13}C NMR (75 MHz) spectra of **18** ($CDCl_3$)

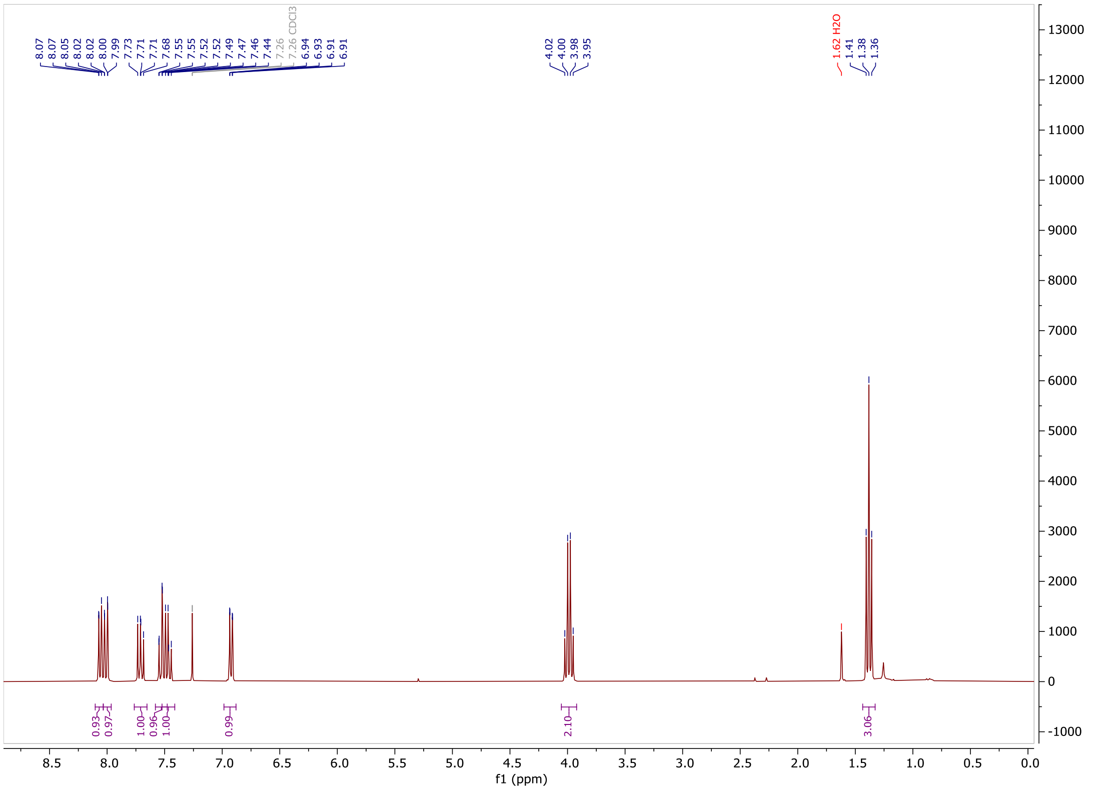

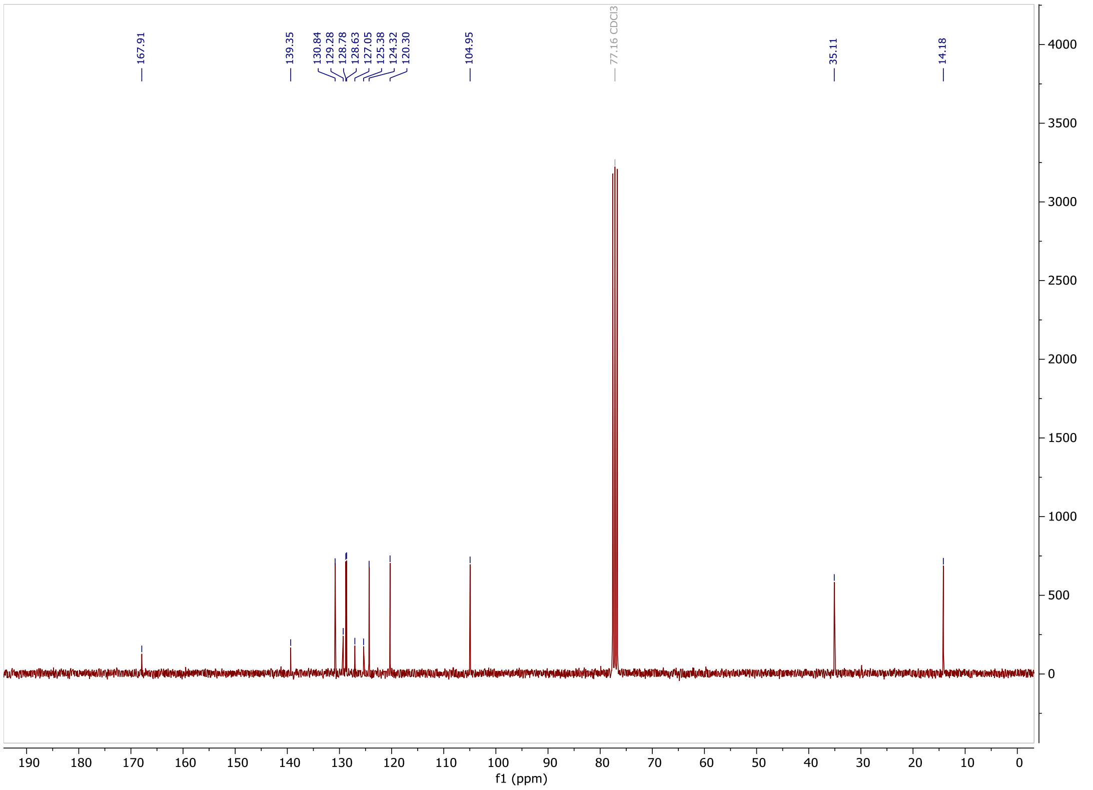

^1H NMR (300 MHz) ^{13}C NMR (75 MHz) spectra of **3** ($CDCl_3$)

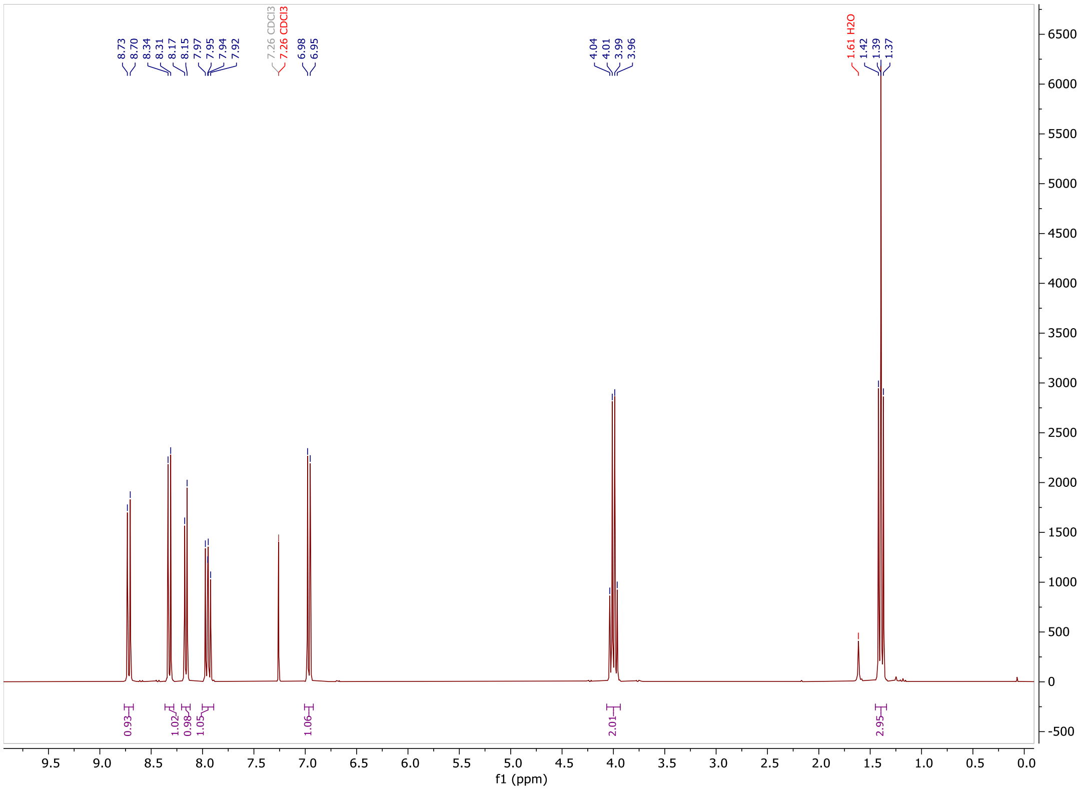

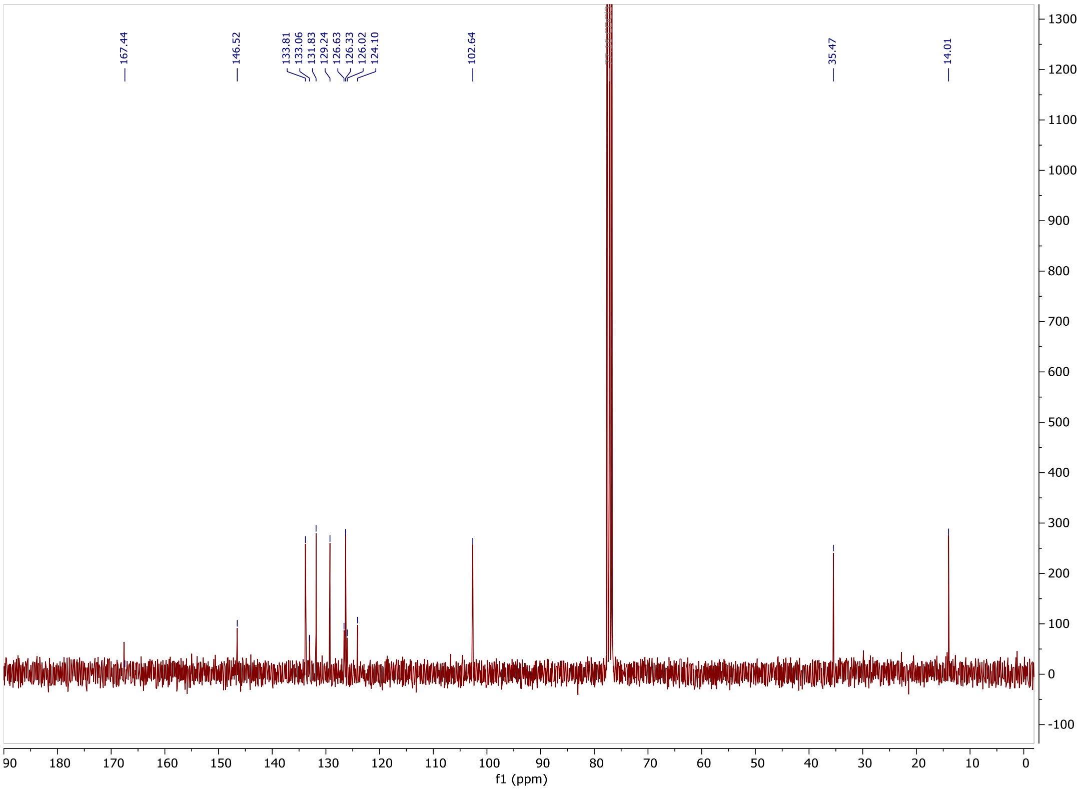

^{1}H NMR (400 MHz) ^{13}C NMR (101 MHz) spectra of **6** ($CDCl_3$)

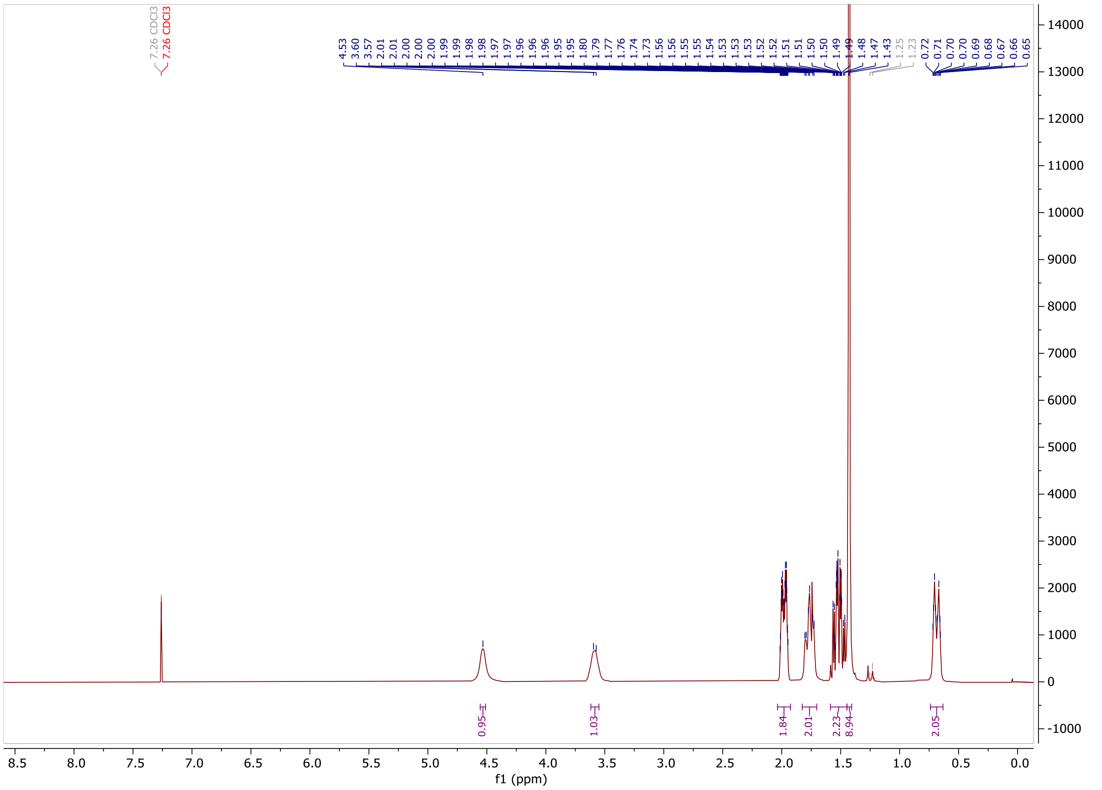

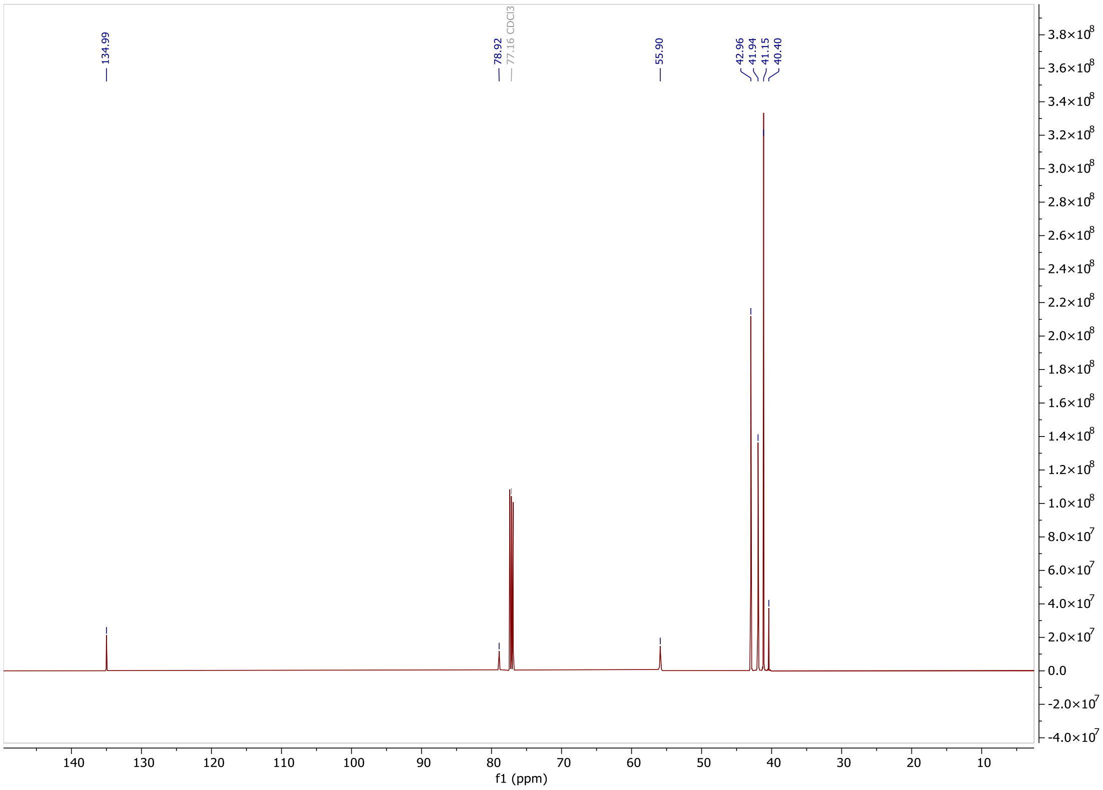

^{1}H NMR (300 MHz) ^{13}C NMR (75 MHz) spectra of **7** ($CDCl_3$)

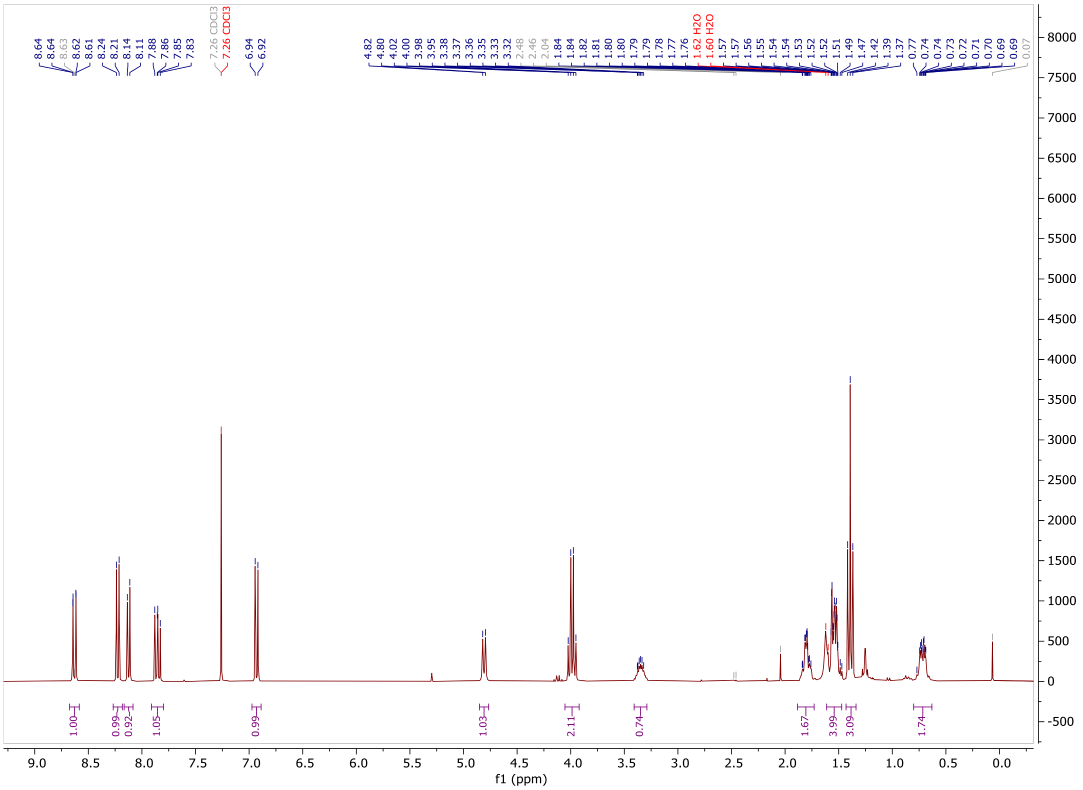

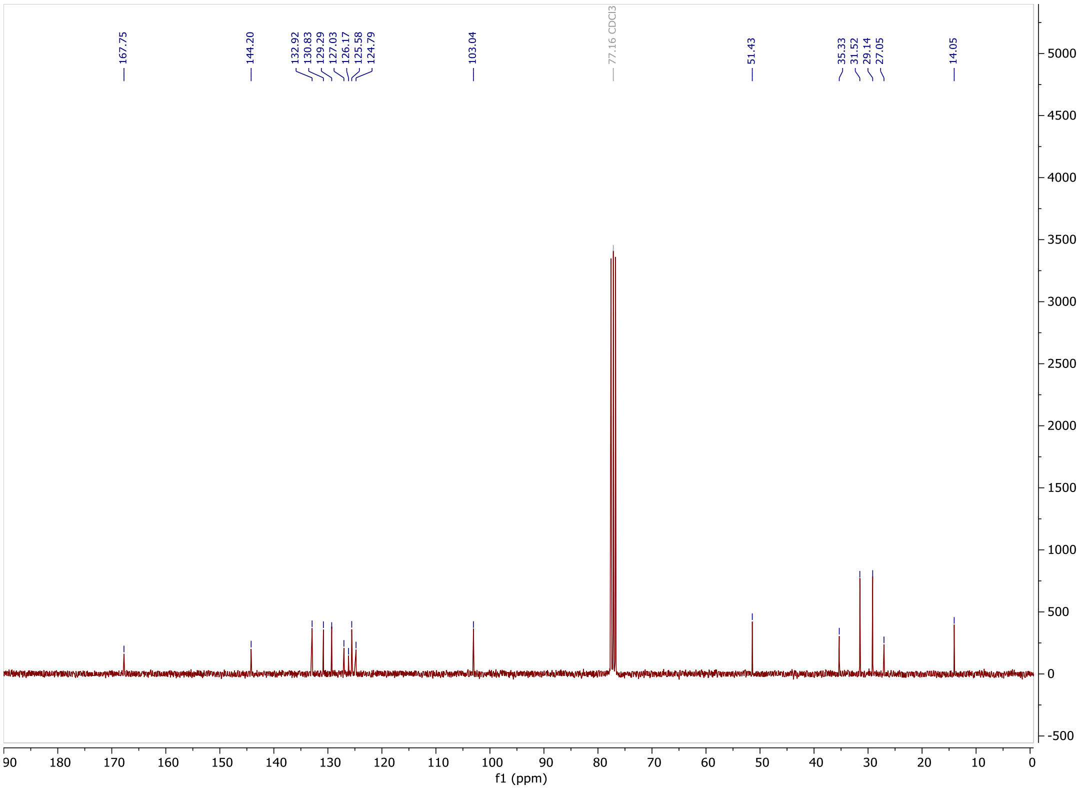

^{1}H NMR (300 MHz) ^{13}C NMR (75 MHz) spectra of **PAL-1** ($CDCl_3$)

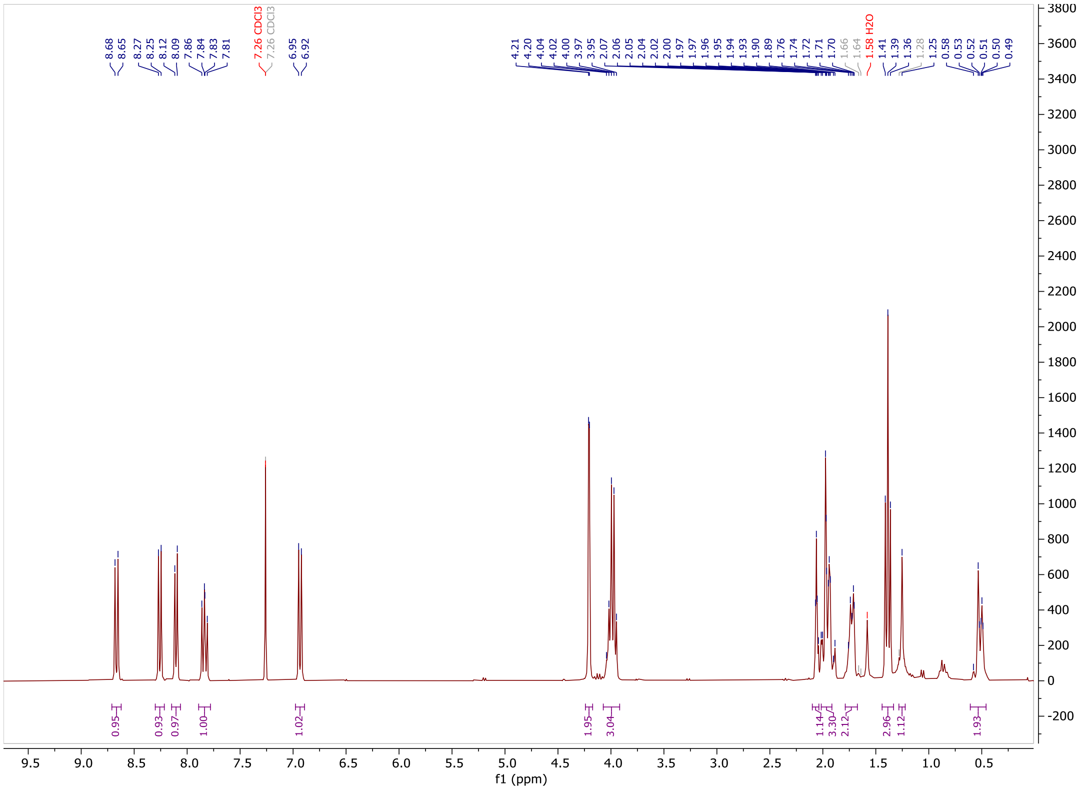

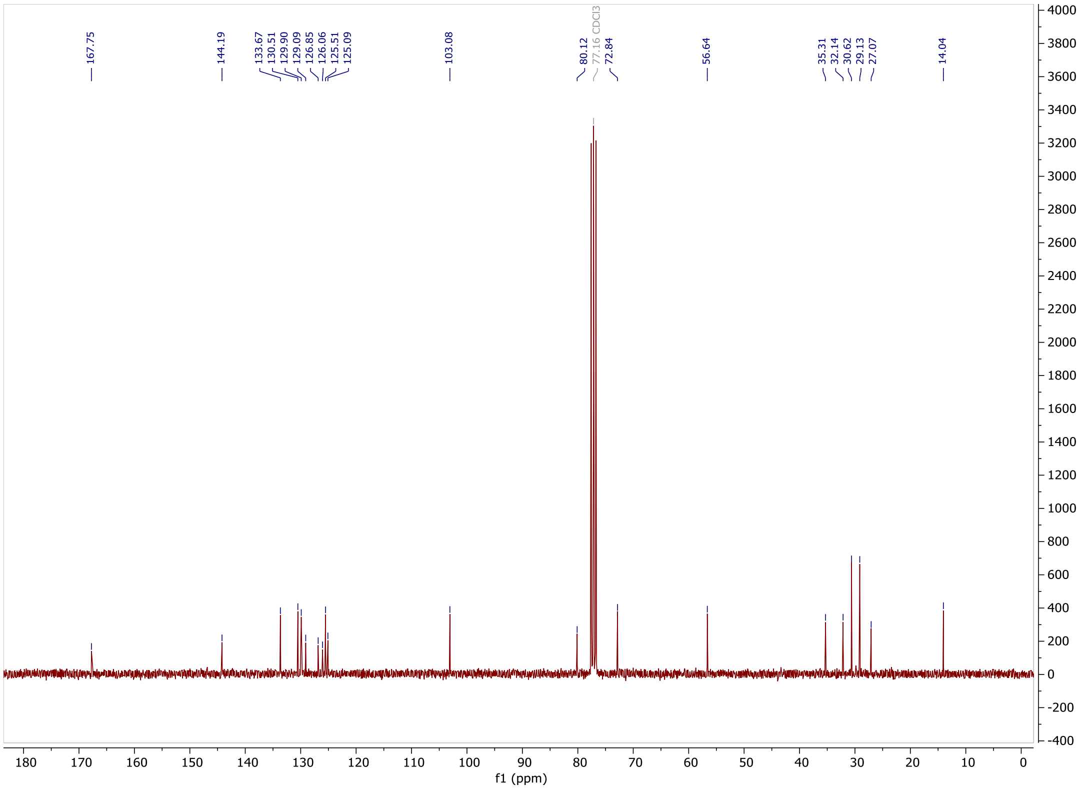

^{1}H NMR (300 MHz) ^{13}C NMR (75 MHz) spectra of **8a** ($CDCl_3$)

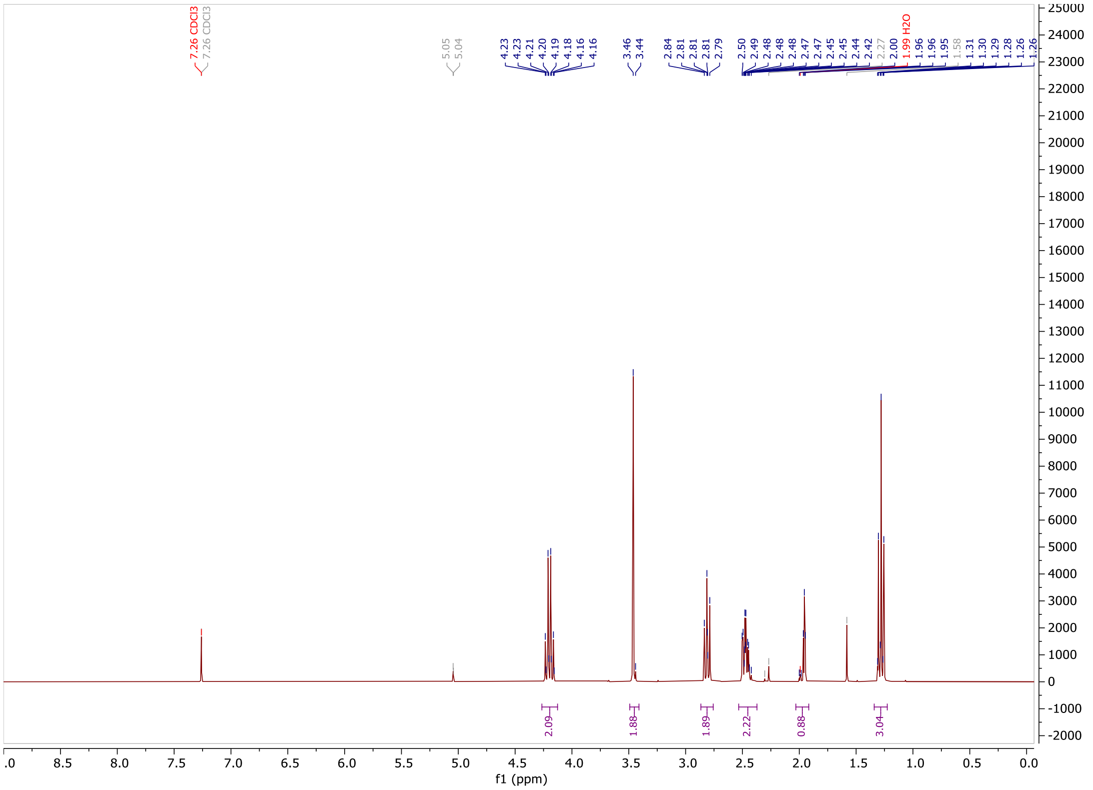

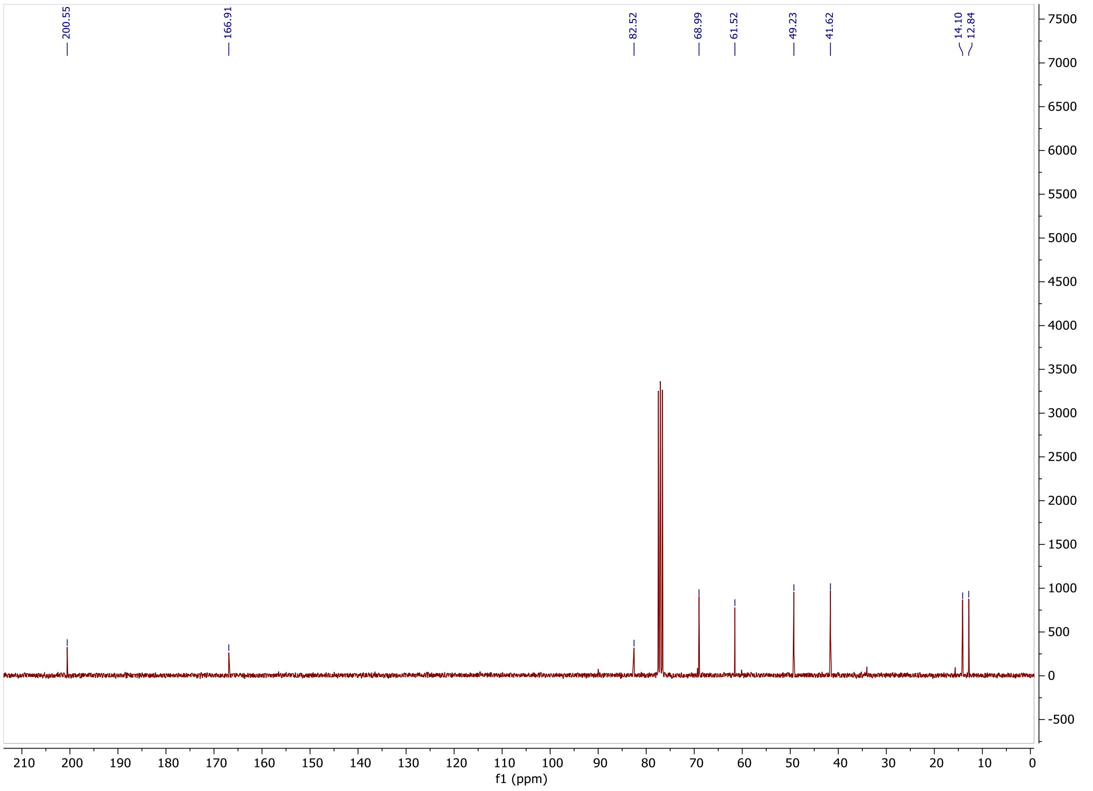

^1H NMR (300 MHz) ^{13}C NMR (75 MHz) spectra of **10a** ($CDCl_3$)

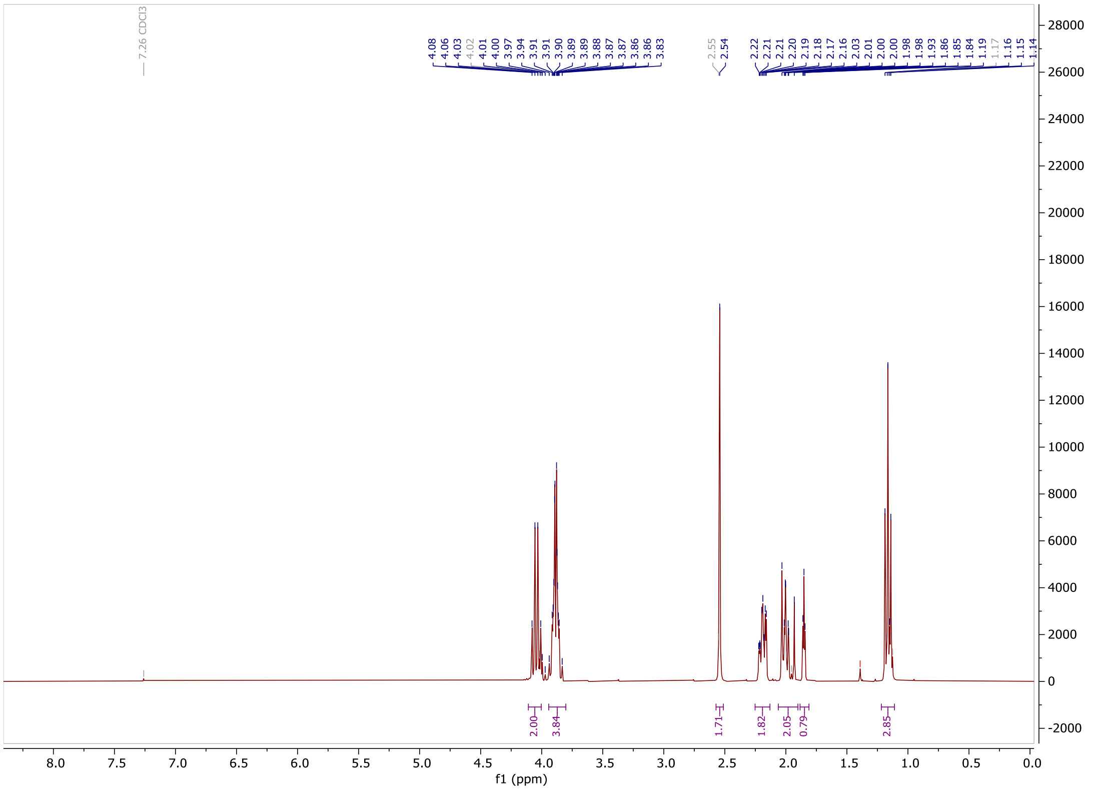

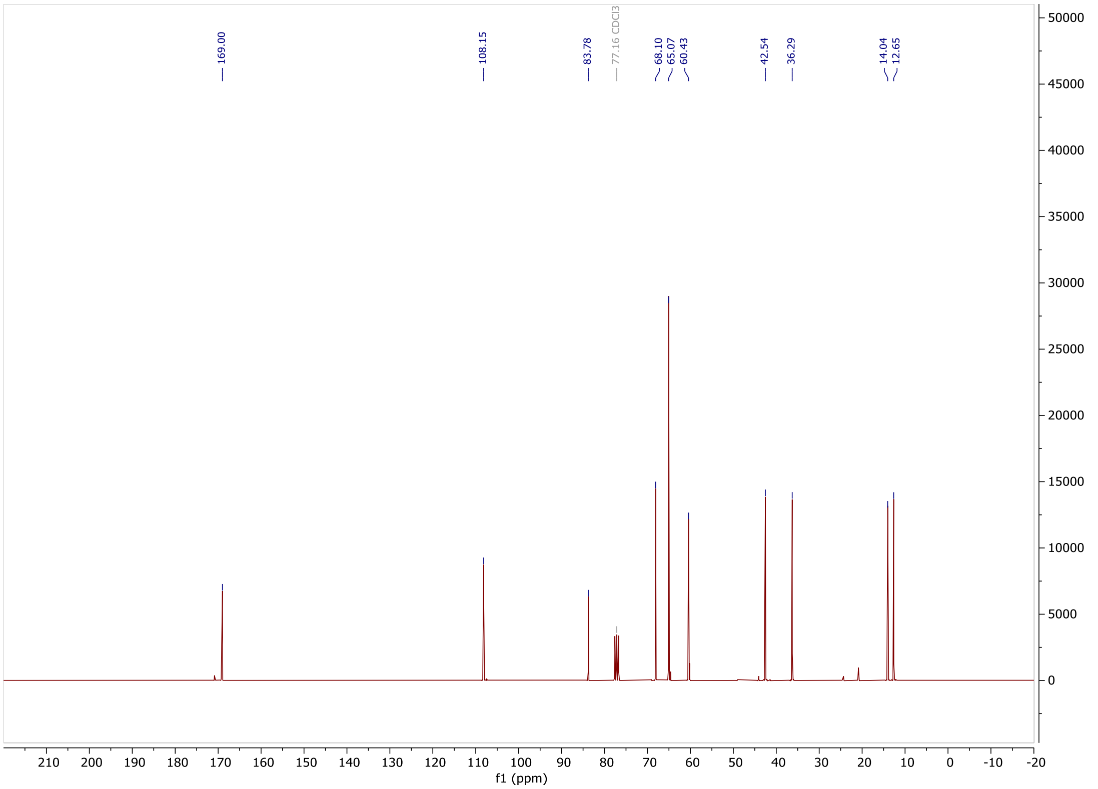

^{1}H NMR (400 MHz) ^{13}C NMR (101 MHz) spectra of **11a** ($CDCl_3$)

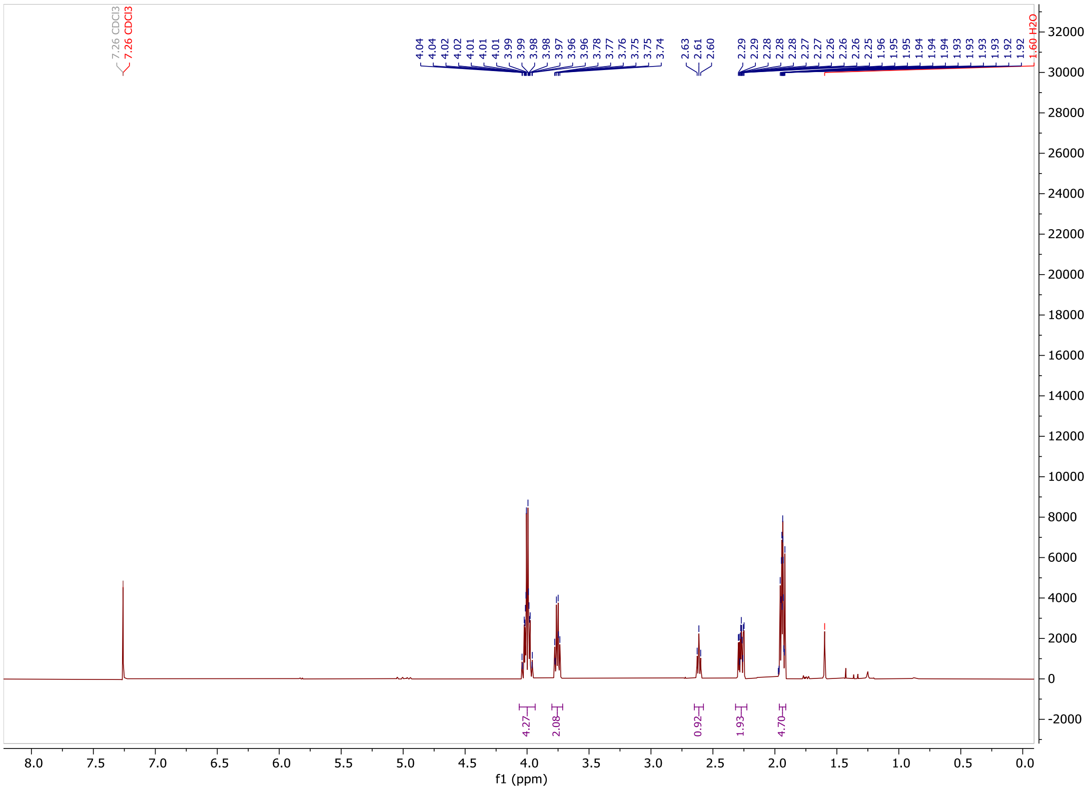

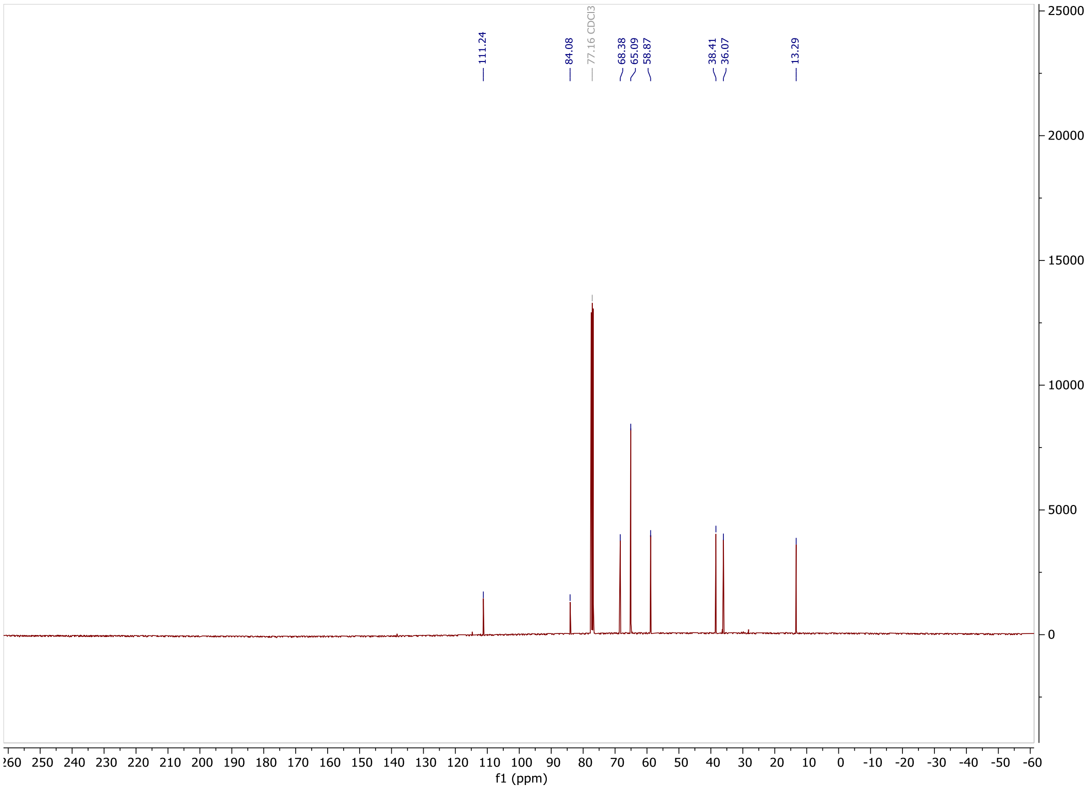

^{1}H NMR (300 MHz) ^{13}C NMR (75 MHz) spectra of **12a** ($CDCl_3$)

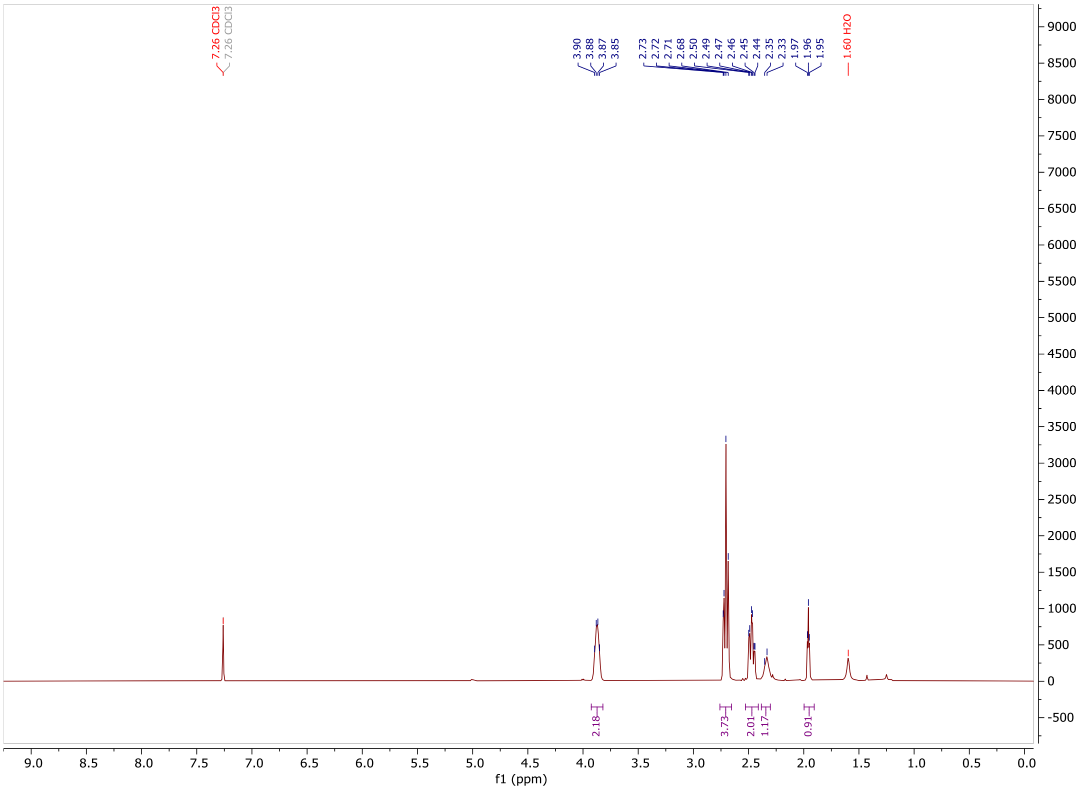

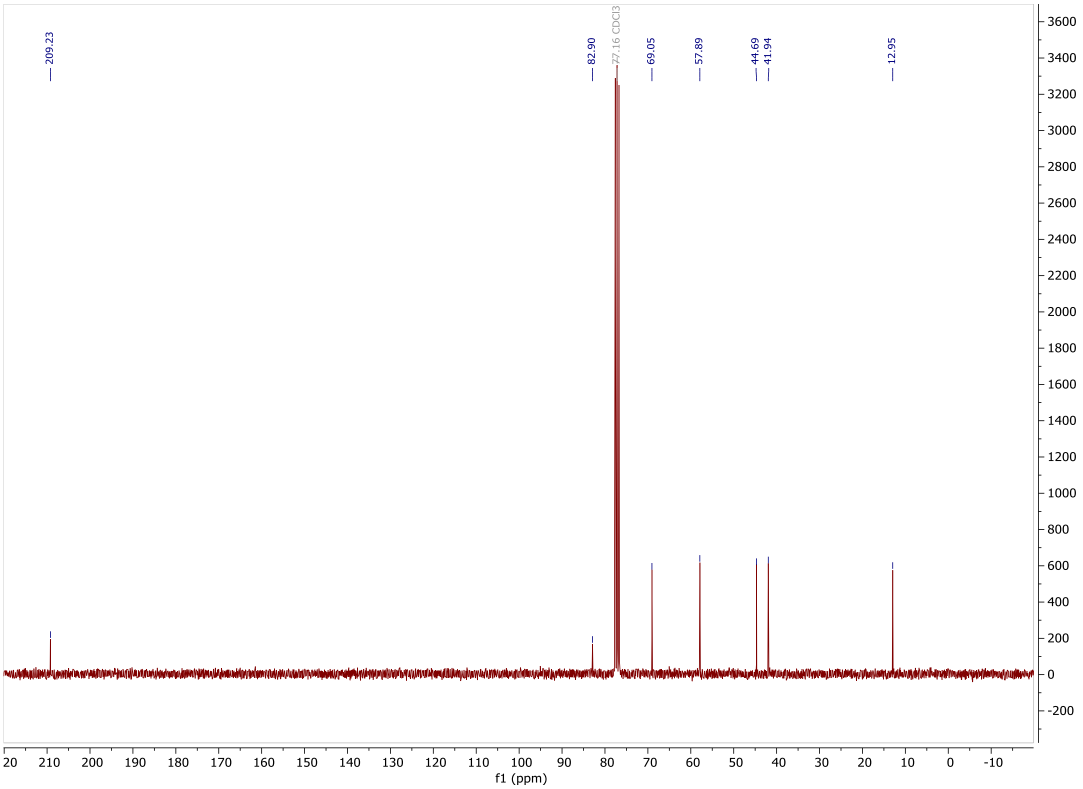

^{1}H NMR (300 MHz) ^{13}C NMR (75 MHz) spectra of **13a** ($CDCl_3$)

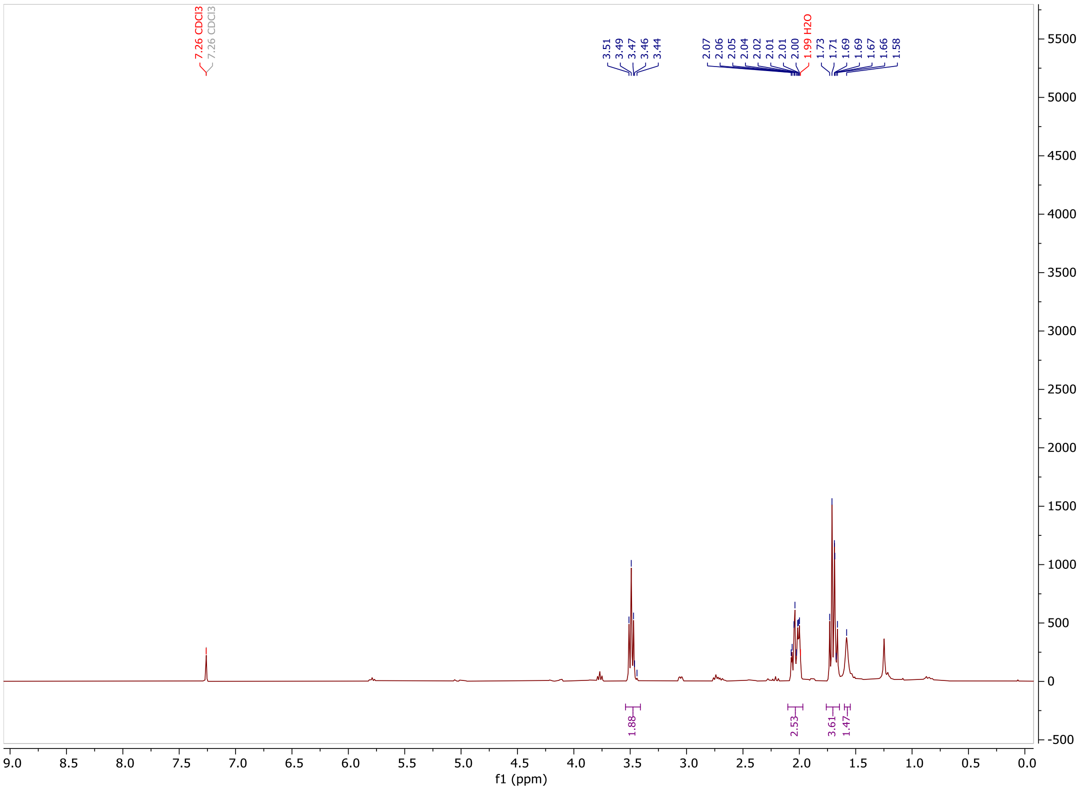

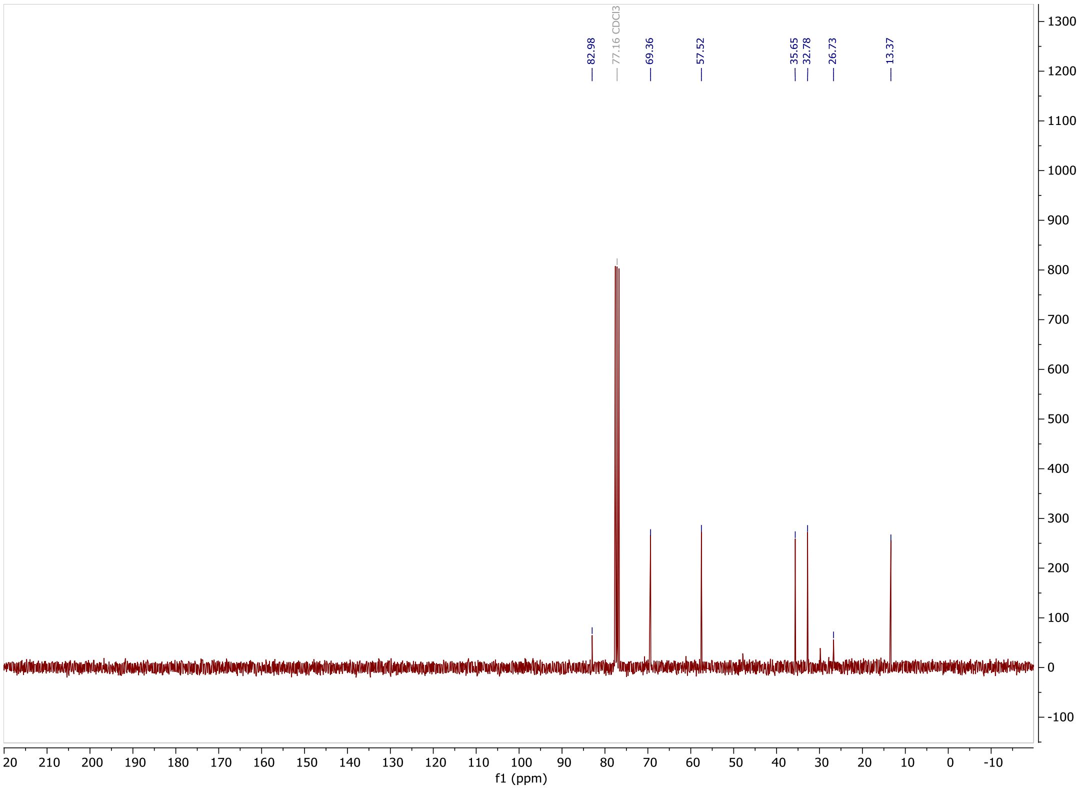

^{1}H NMR (300 MHz), ^{13}C NMR (75 MHz) spectra of **14a** ($CDCl_3$)

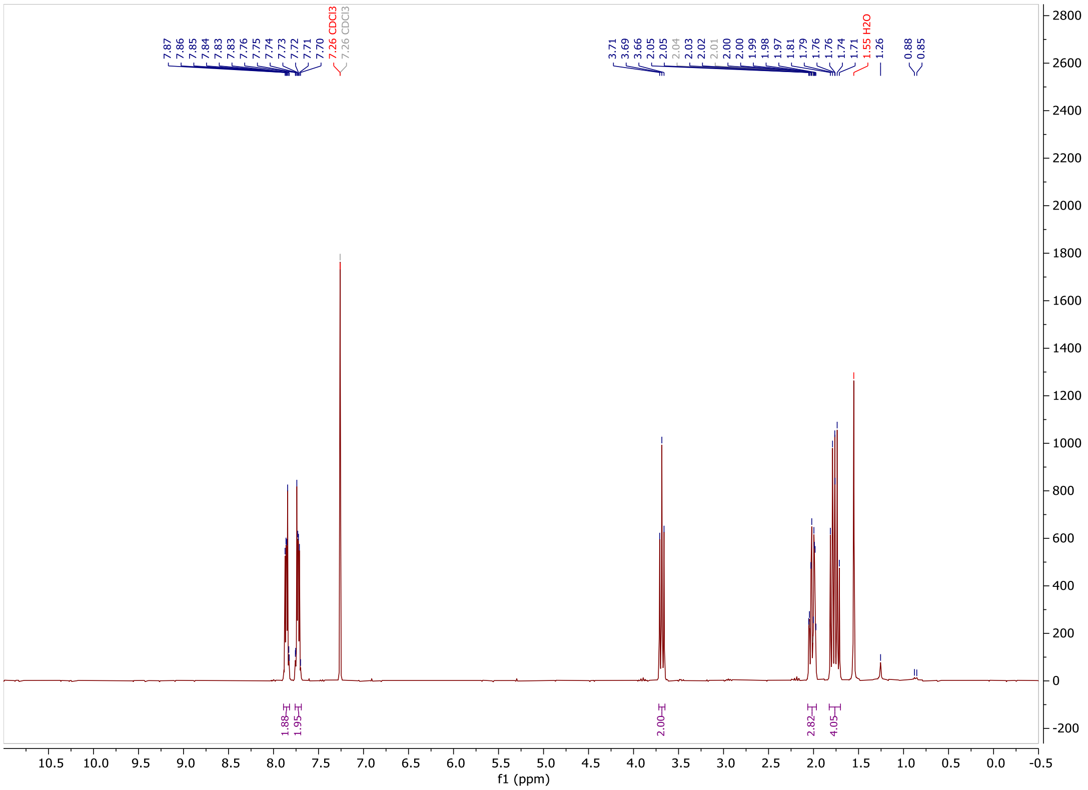

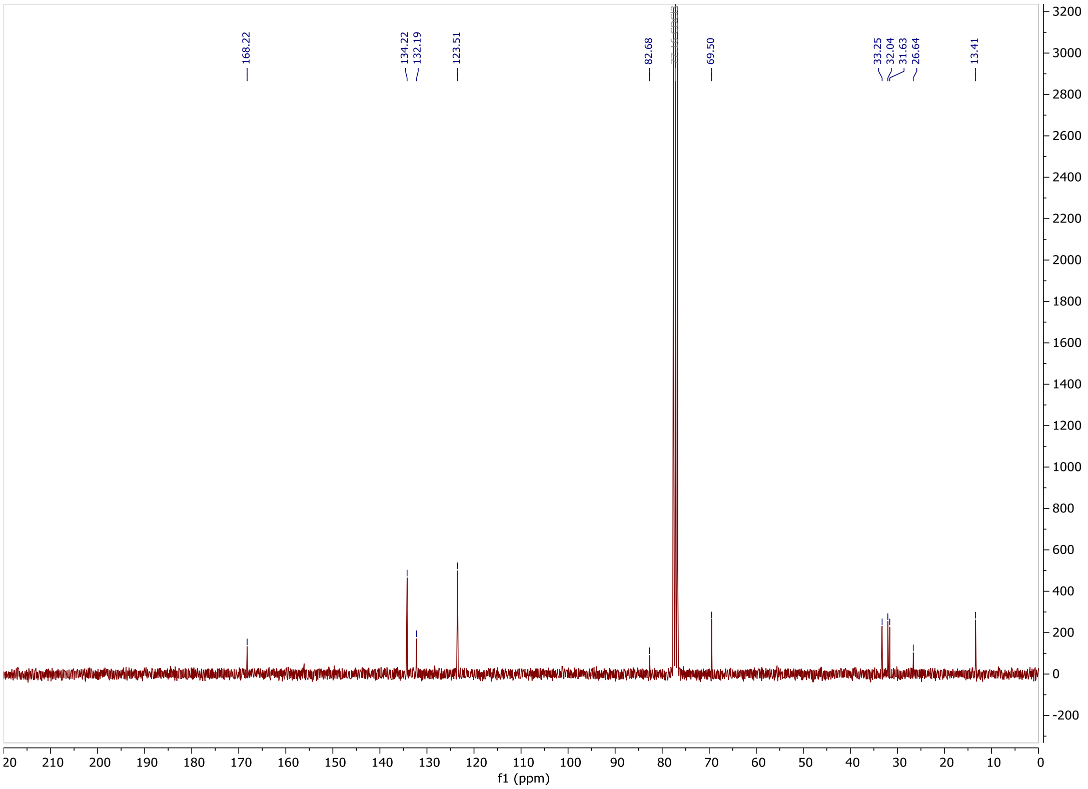

^{1}H NMR (300 MHz), ^{13}C NMR (75 MHz) spectra of **15a** ($CDCl_3$)

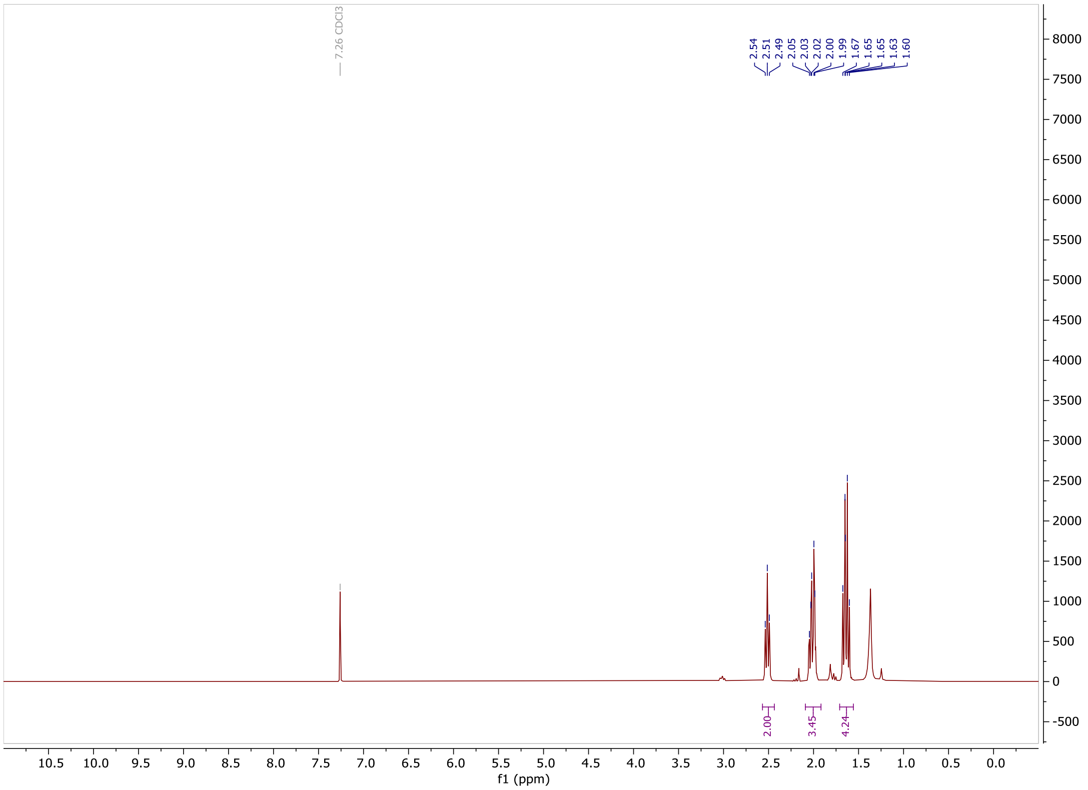

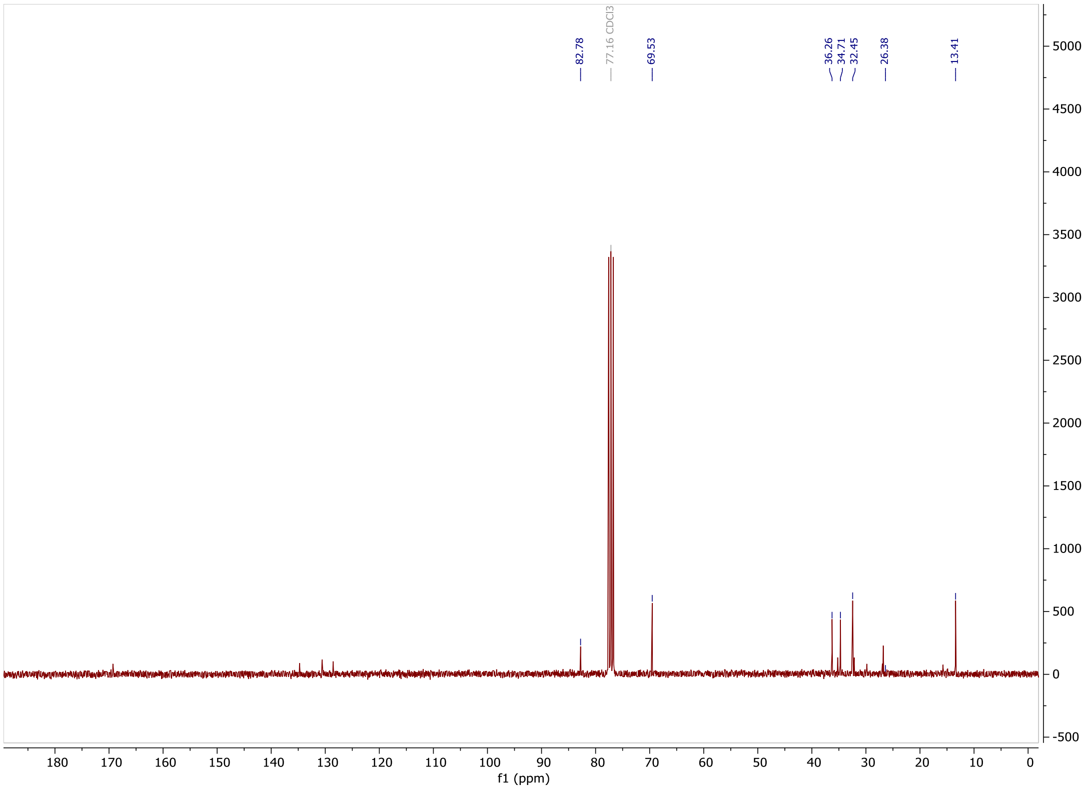

^{1}H NMR (400 MHz), ^{13}C NMR (101 MHz) spectra of **PAL-2** (CD_3OD)

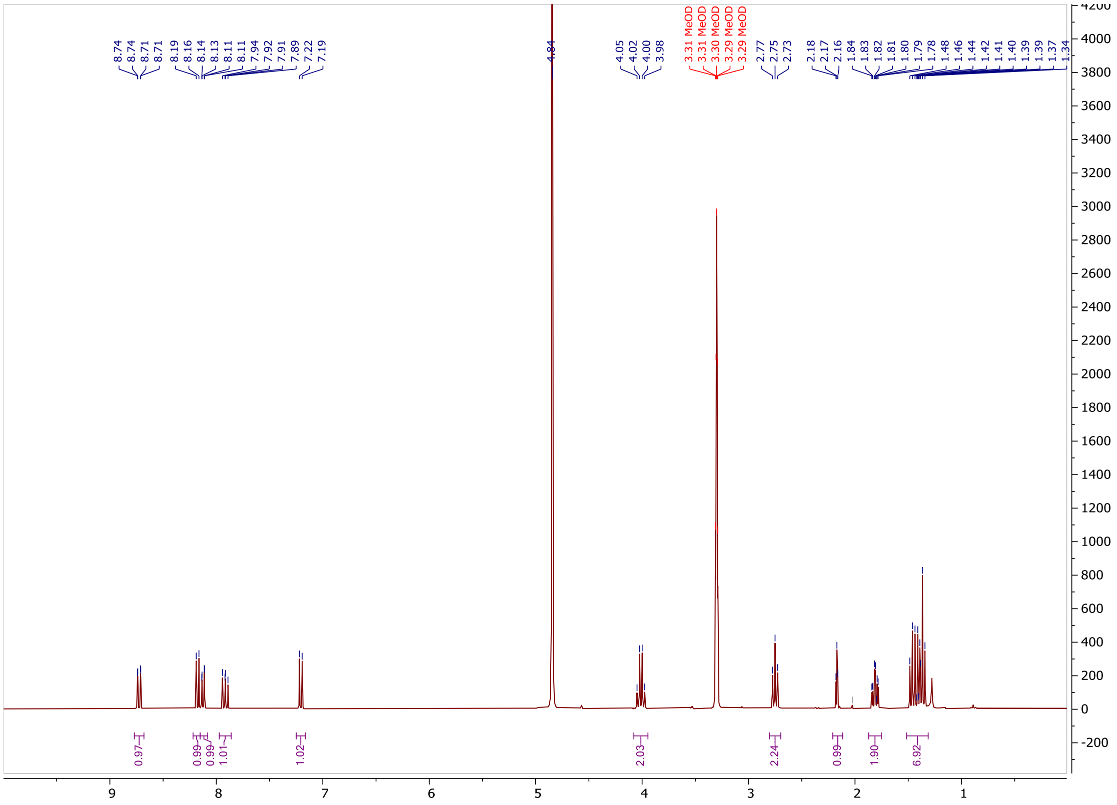

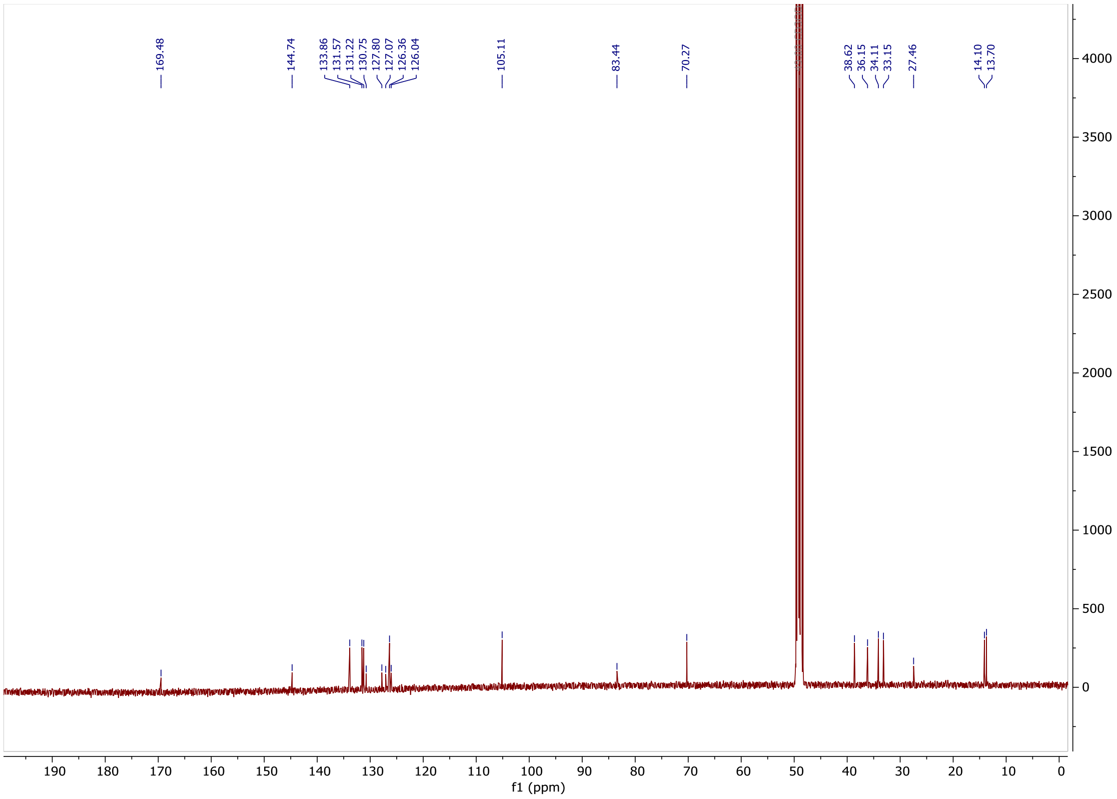

^{1}H NMR (300 MHz), ^{13}C NMR (75 MHz) spectra of **9** ($CDCl_3$)

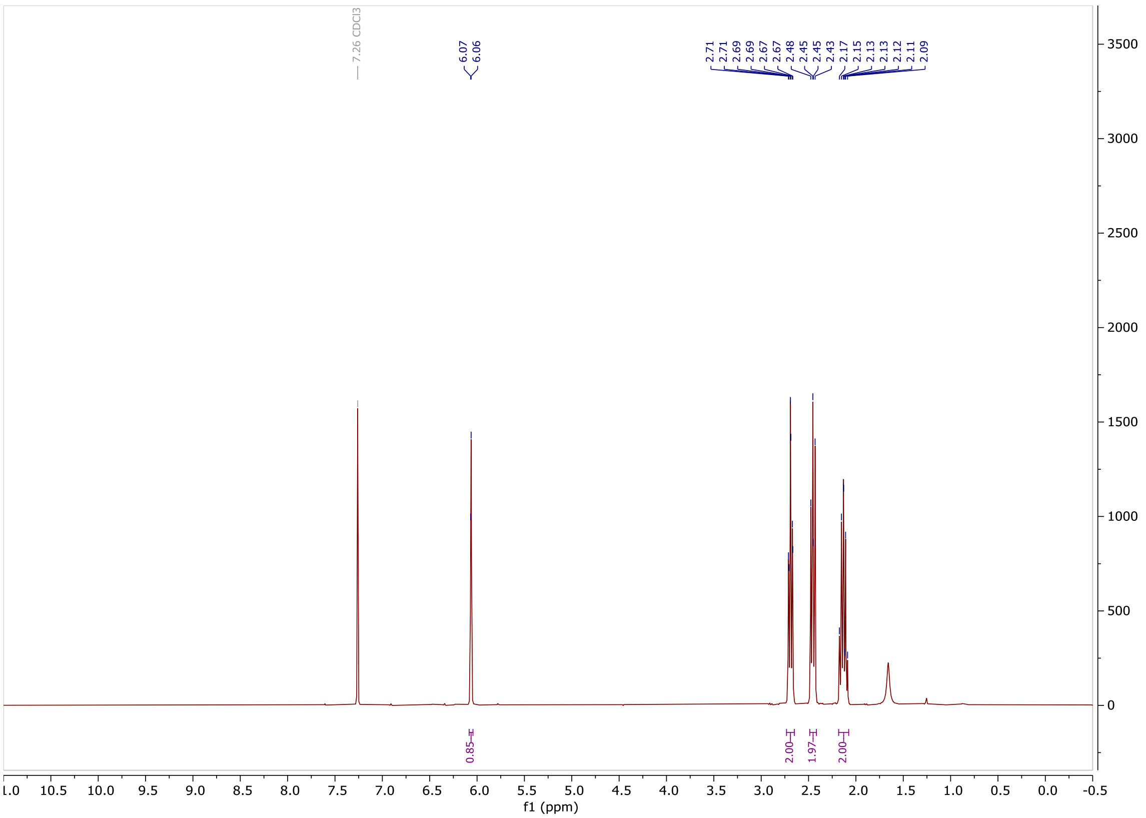

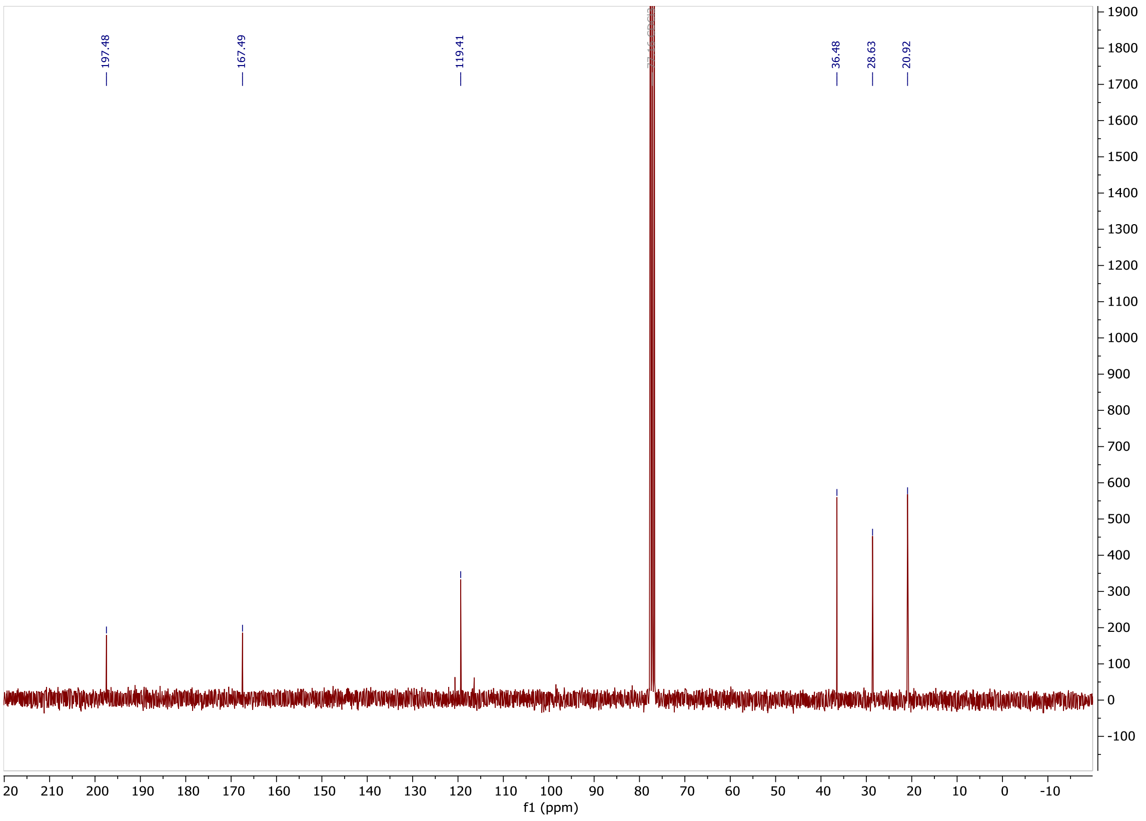

^{1}H NMR (300 MHz), ^{13}C NMR (75 MHz) spectra of **8b** ($CDCl_3$)

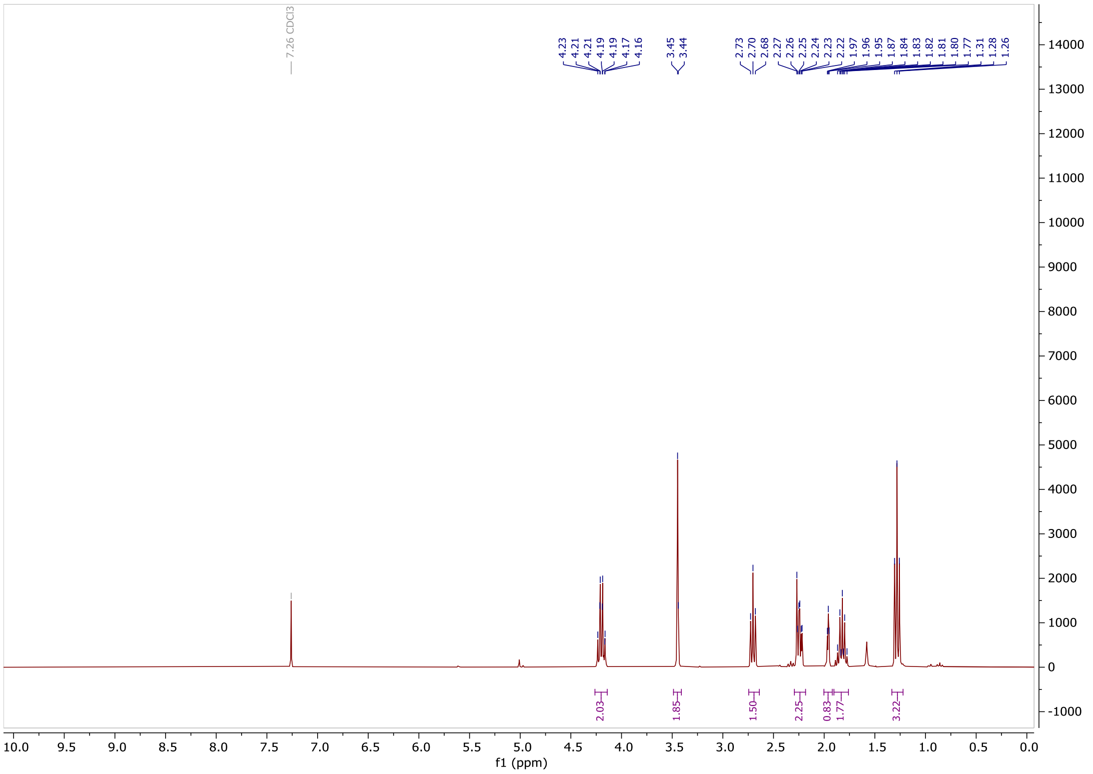

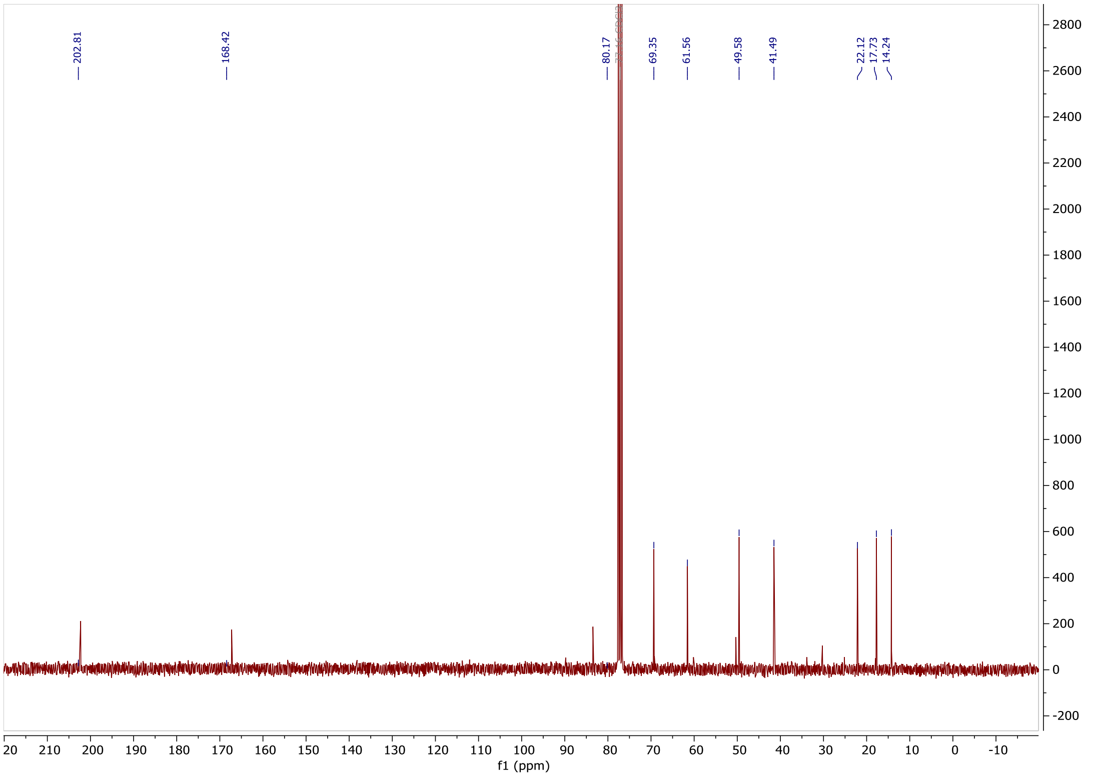

^{1}H NMR (300 MHz), ^{13}C NMR (75 MHz) spectra of **10b** ($CDCl_3$)

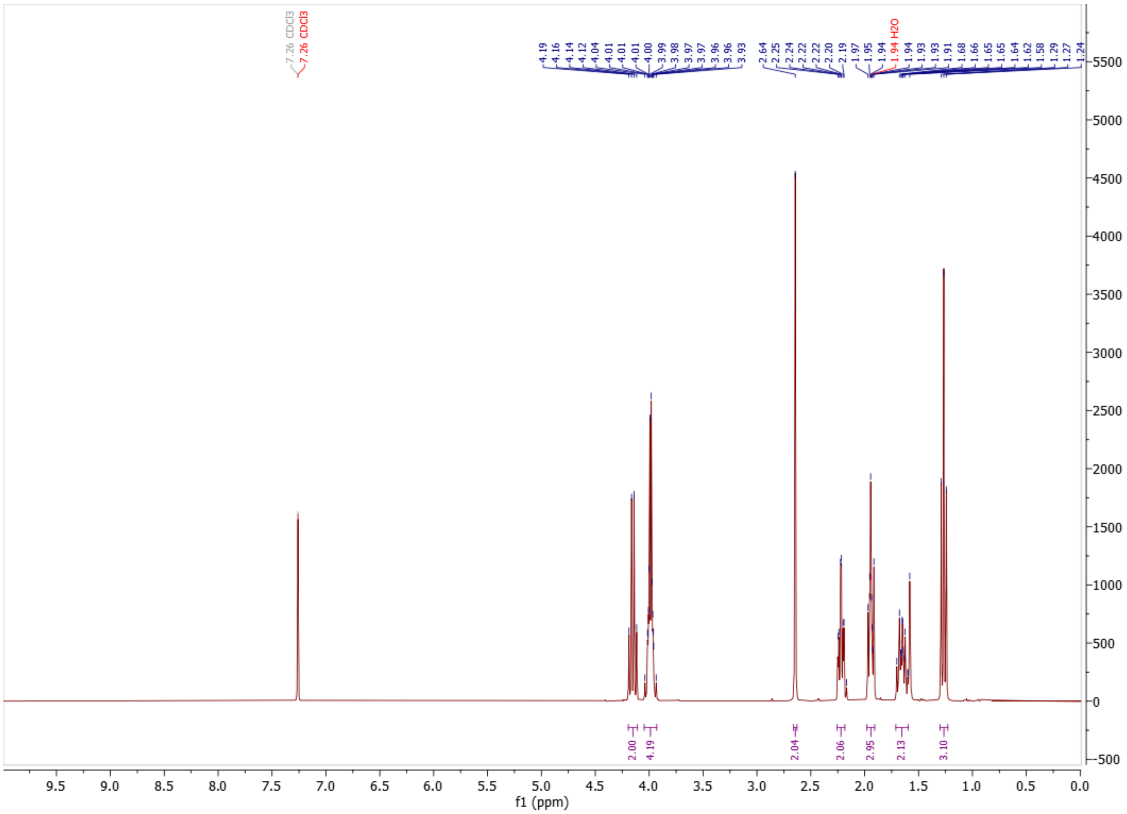

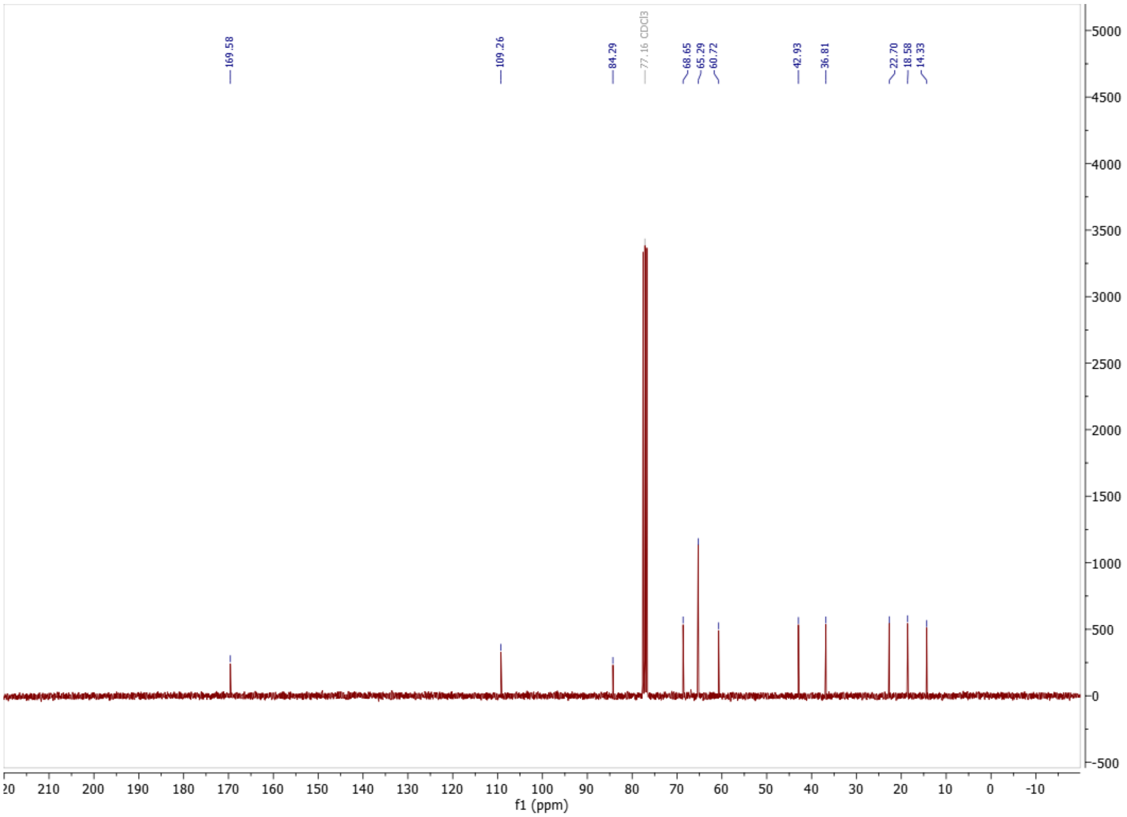

^{1}H NMR (300 MHz), ^{13}C NMR (75 MHz) spectra of **12b** ($CDCl_3$)

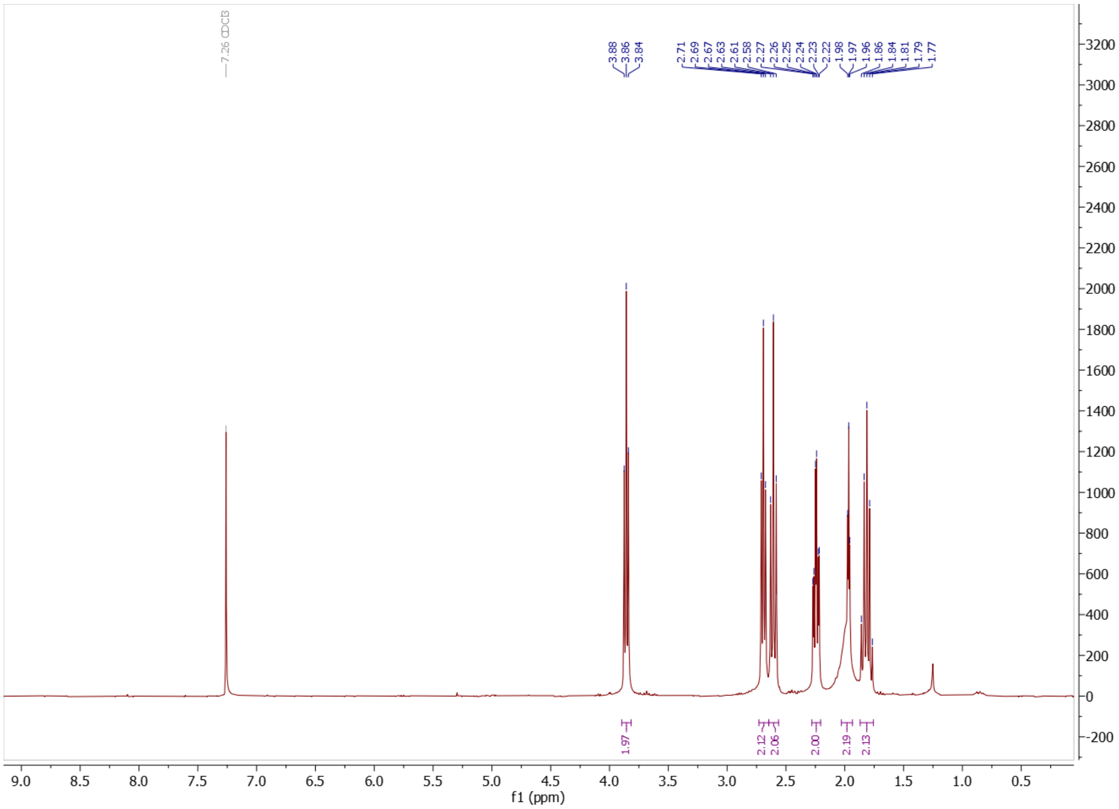

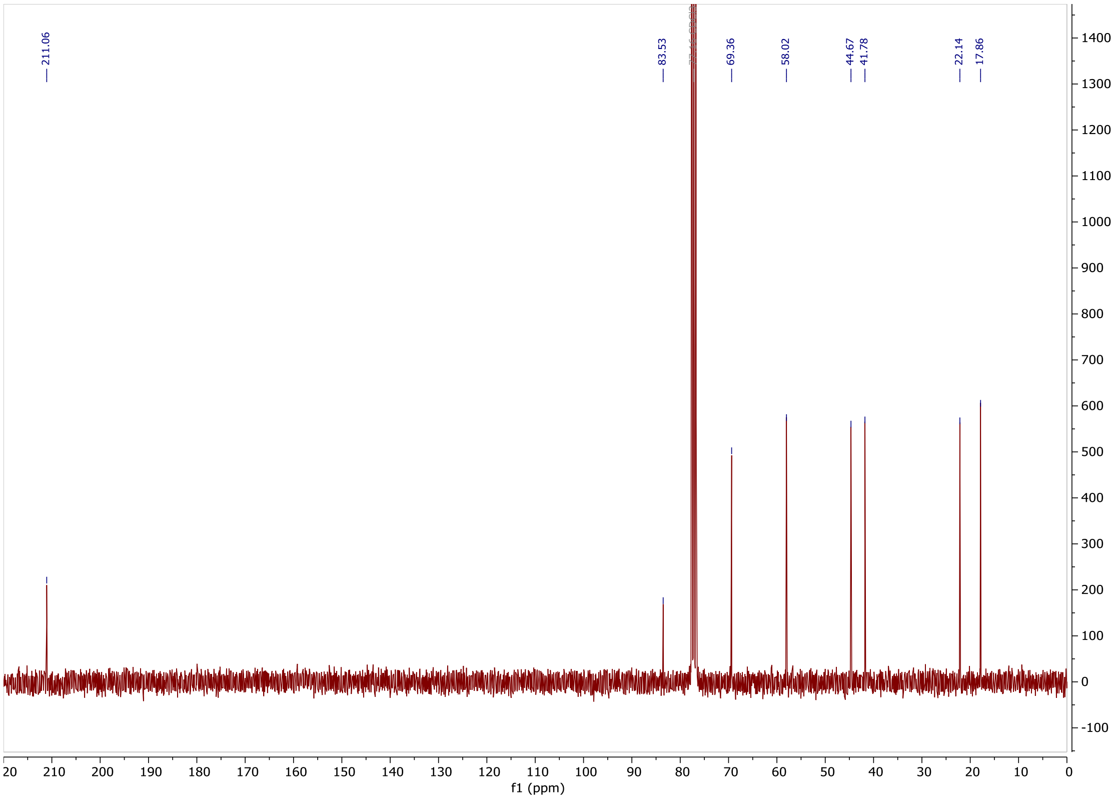

^{1}H NMR (400 MHz), ^{13}C NMR (101 MHz) spectra of **13b** ($CDCl_3$)

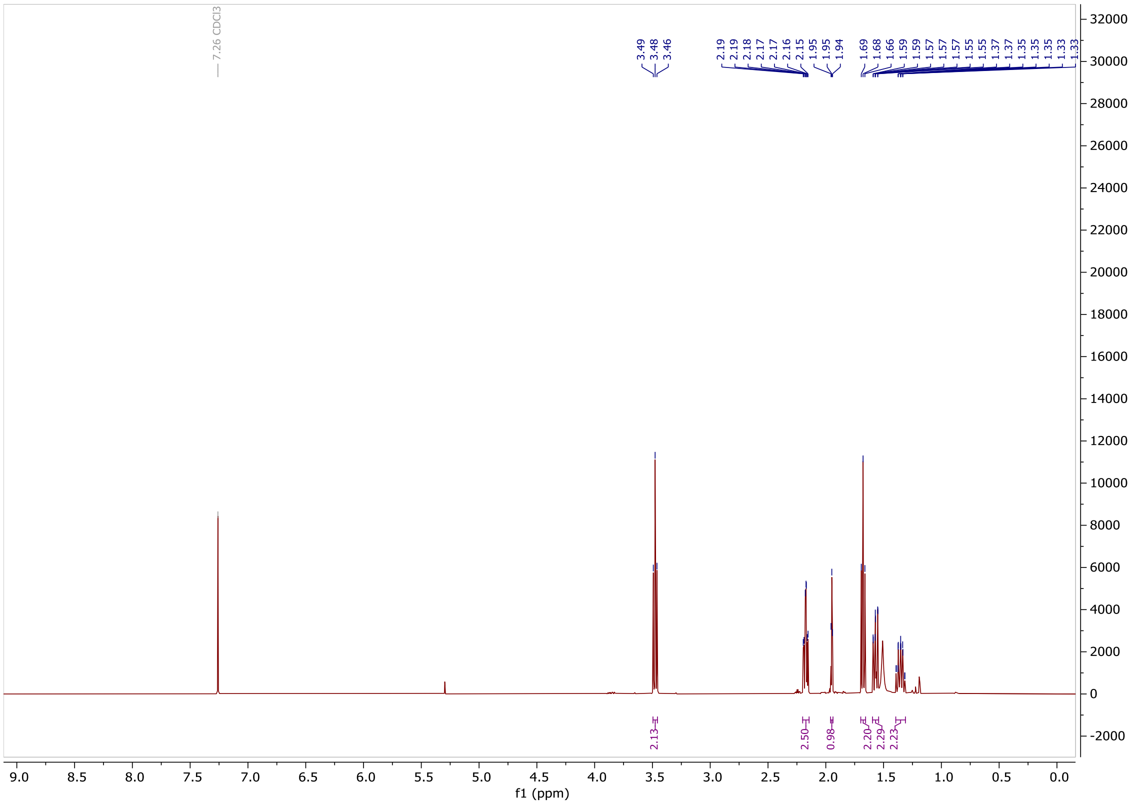

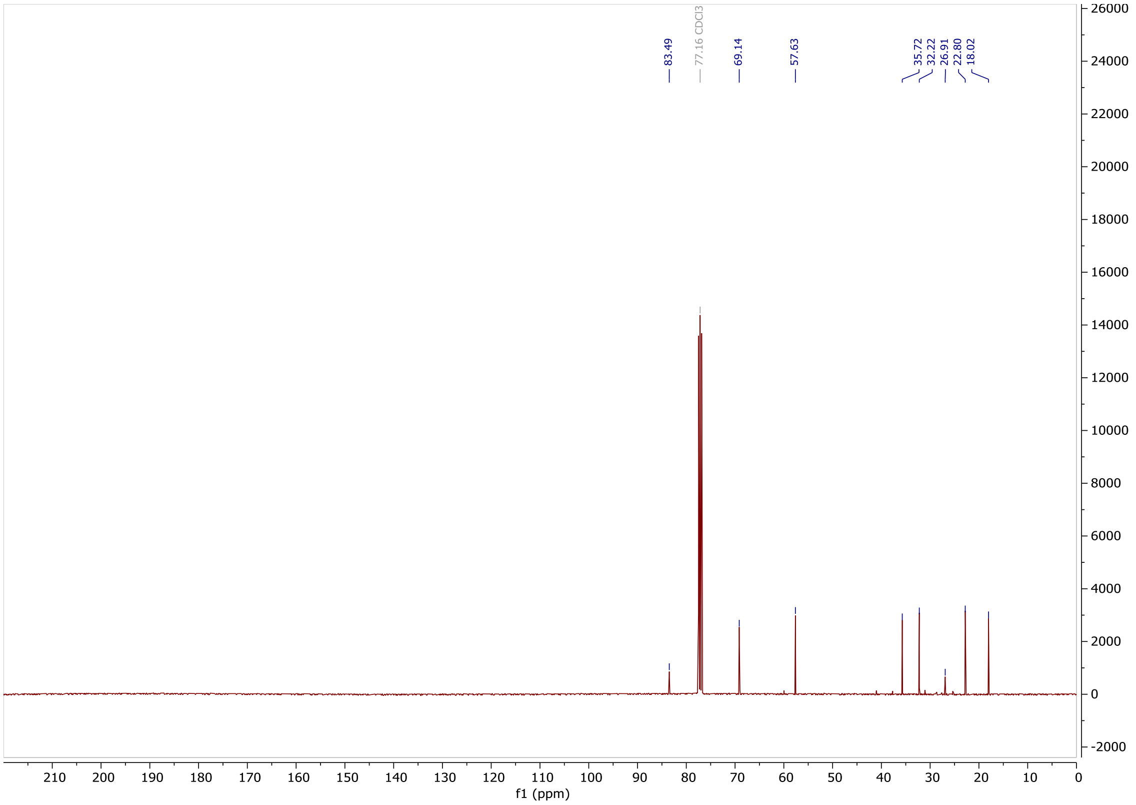

^{1}H NMR (400 MHz), ^{13}C NMR (101 MHz) spectra of **14b** ($CDCl_3$)

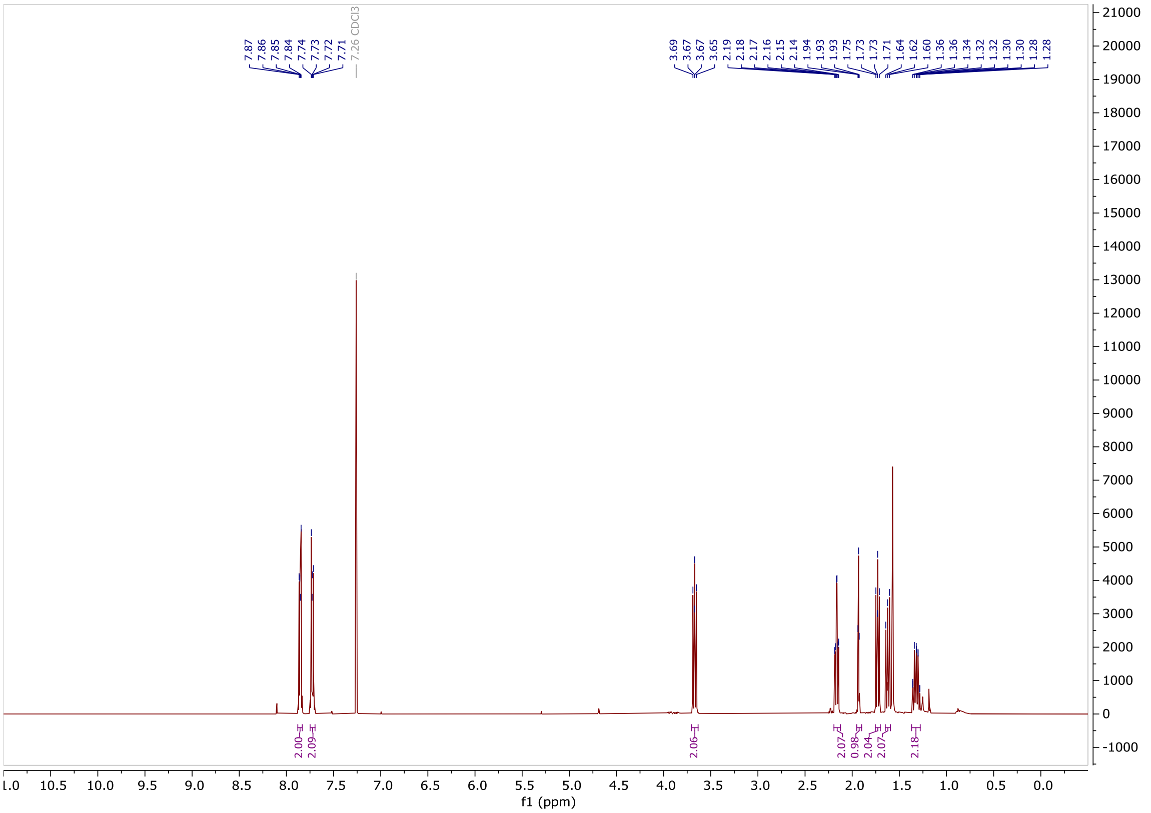

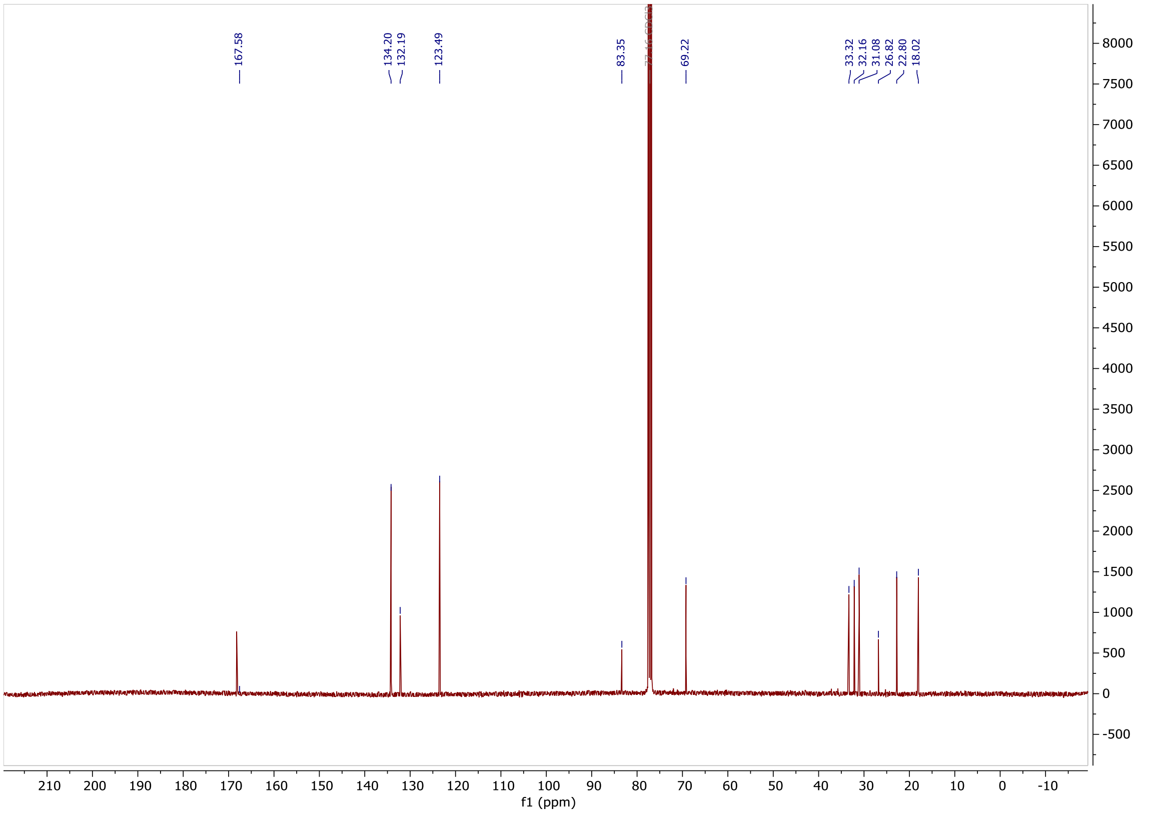

^{1}H NMR (400 MHz), ^{13}C NMR (101 MHz) spectra of **15b** ($CDCl_3$)

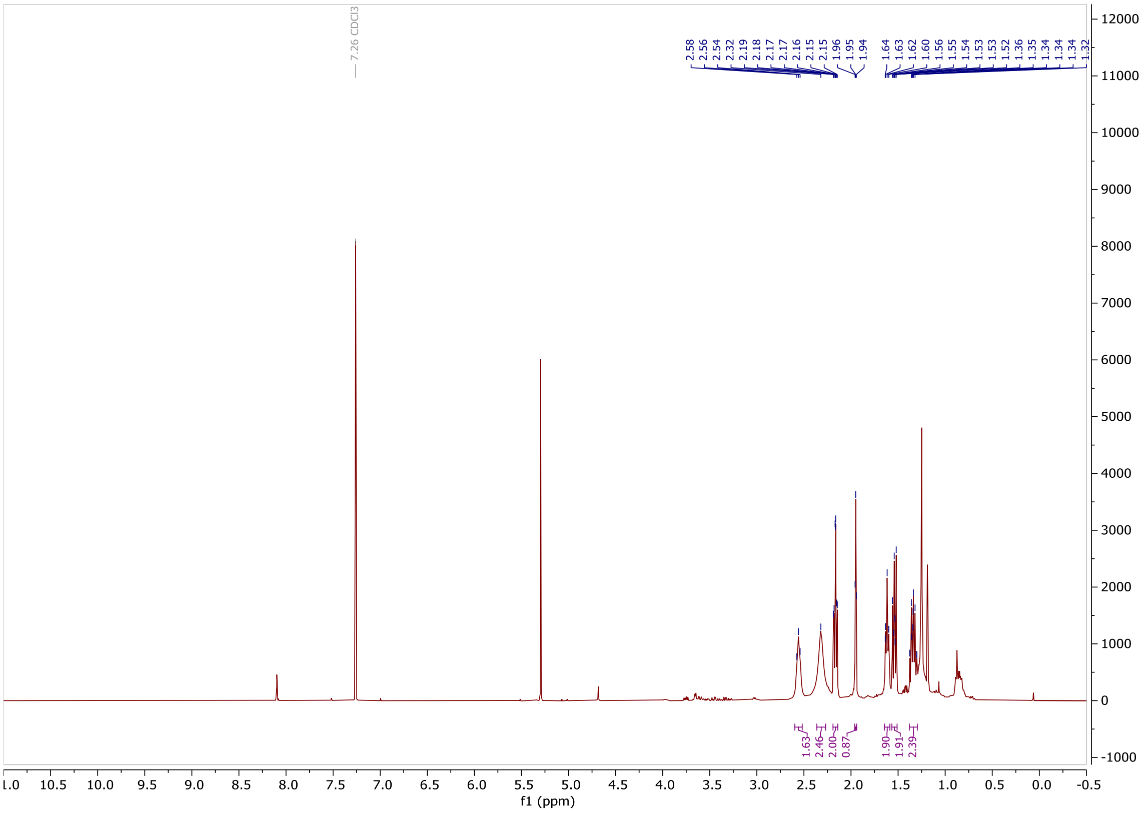

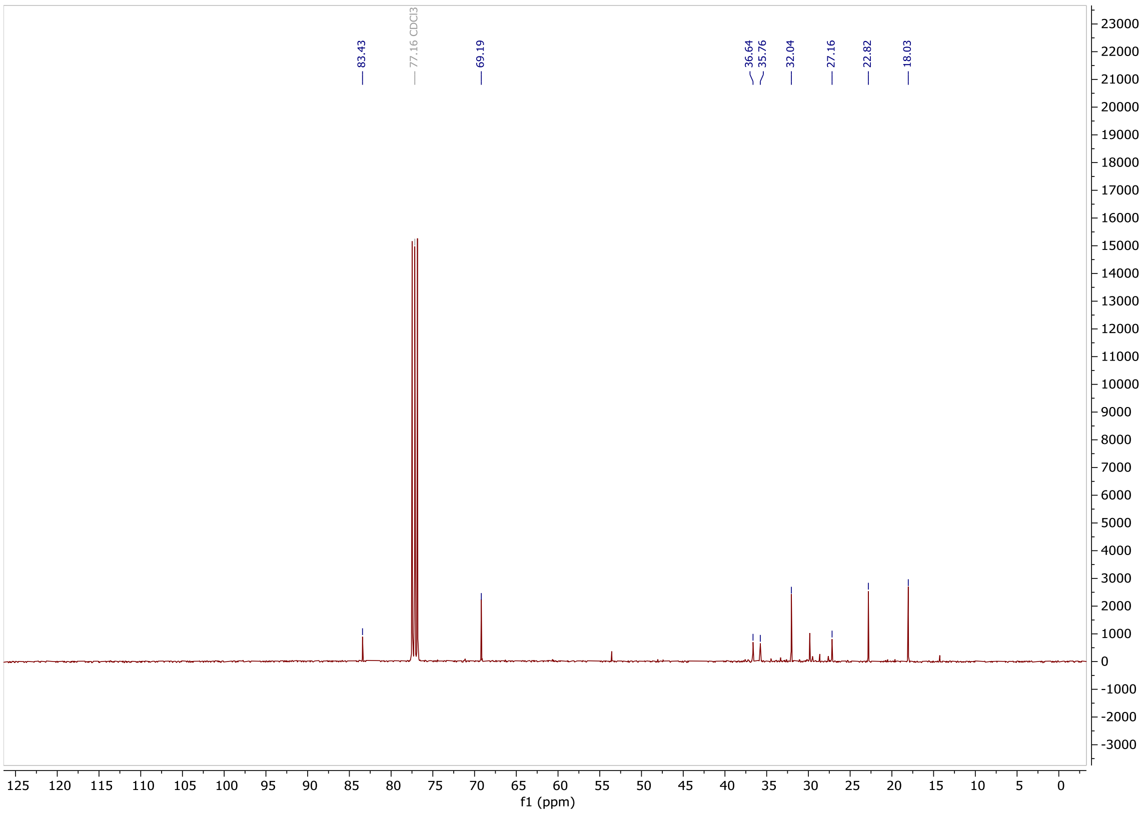

^{1}H NMR (400 MHz), ^{13}C NMR (101 MHz) spectra of **PAL-3** (CD_3OD)

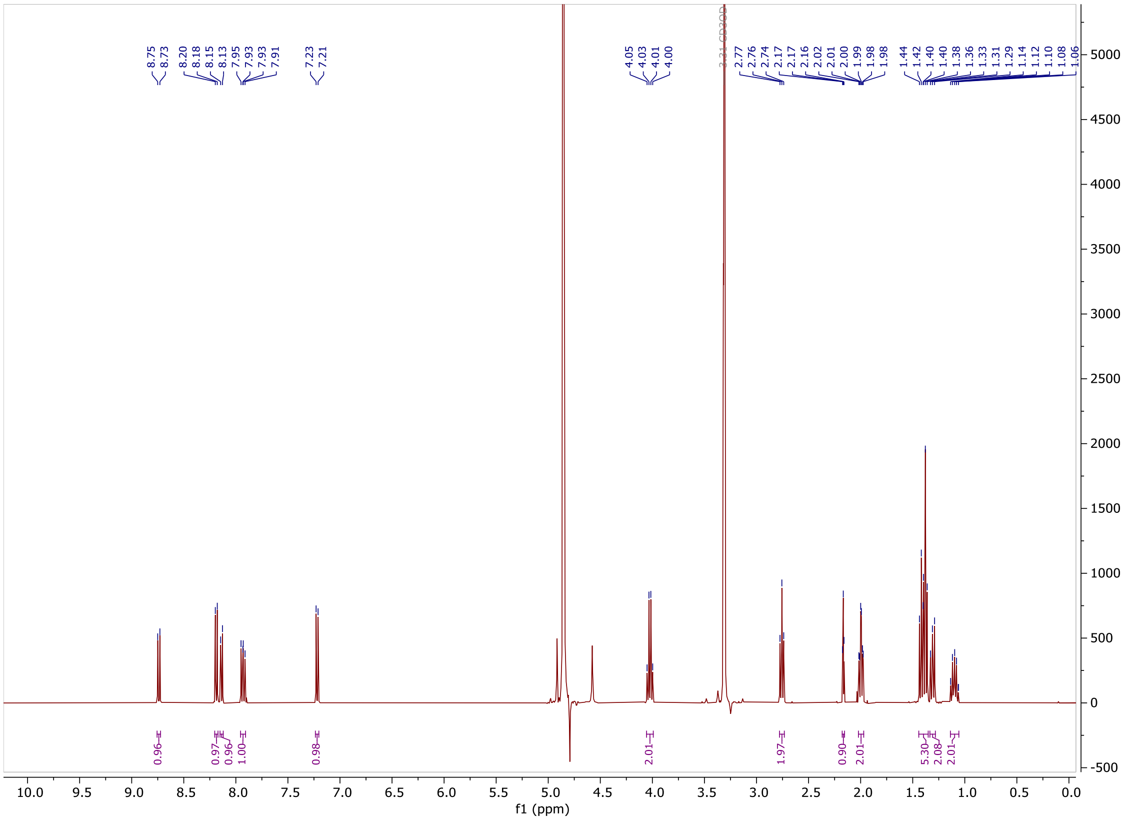

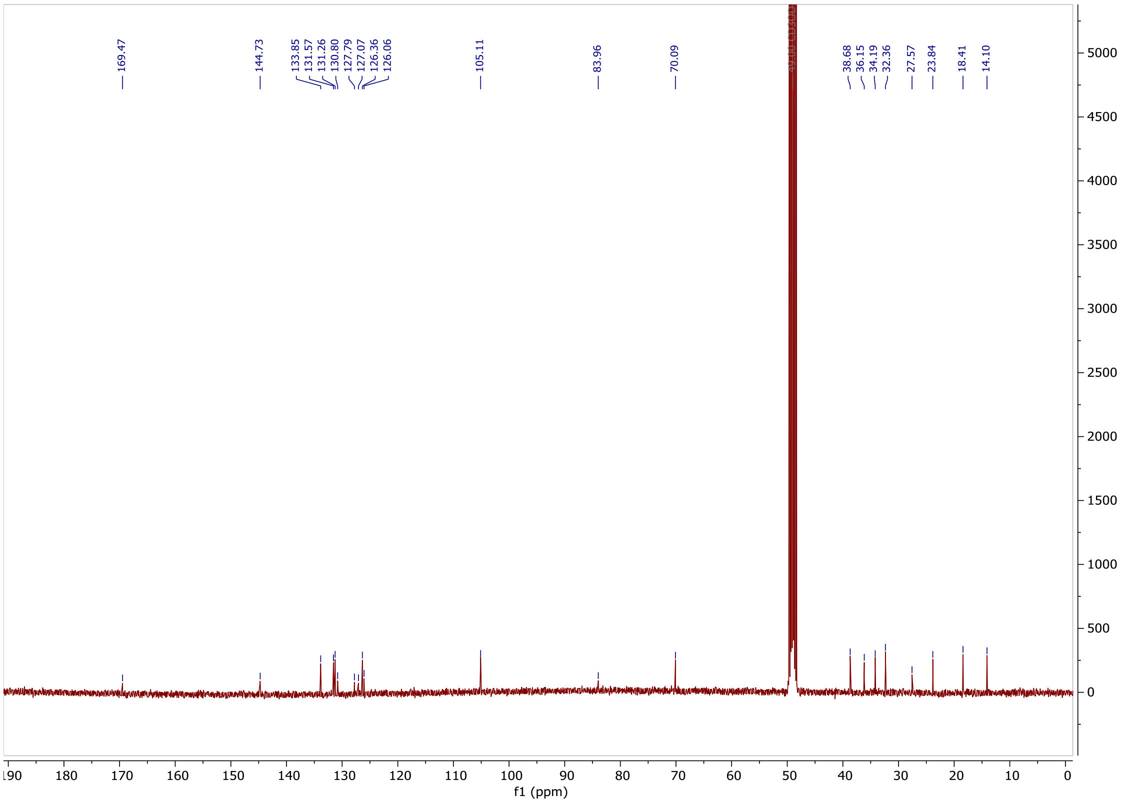

Supplement: Supplementary file 1 — Supporting Information [file OPEN-14-e202500119-s001.pdf]
